# PtoNF-YC9-SRMT-PtoRD26 module regulates the high saline tolerance of a triploid poplar

**DOI:** 10.1186/s13059-022-02718-7

**Published:** 2022-07-07

**Authors:** Shaofei Tong, Yubo Wang, Ningning Chen, Deyan Wang, Bao Liu, Weiwei Wang, Yang Chen, Jianquan Liu, Tao Ma, Yuanzhong Jiang

**Affiliations:** Key Laboratory for Bio-resources and Eco-environment of Ministry of Education, College of Life Science, Sichuan University, No. 24 South Section 1, Yihuan Road, Chengdu, 610065 China

**Keywords:** ABA, *Populus tomentosa*, PtoRD26, PtoNF-YC9, Salt stress, SRMT, Triploid Chinese white poplar

## Abstract

**Background:**

Sensing and responding to stresses determine the tolerance of plants to adverse environments. The triploid Chinese white poplar is widely cultivated in North China because of its adaptation to a wide range of habitats including highly saline ones. However, its triploid genome complicates any detailed investigation of the molecular mechanisms underlying its adaptations.

**Results:**

We report a haplotype-resolved genome of this triploid poplar and characterize, using reverse genetics and biochemical approaches, a *MYB* gene, *SALT RESPONSIVE MYB TRANSCRIPTION FACTOR* (*SRMT*), which combines *NUCLEAR FACTOR Y SUBUNIT C* 9 (*PtoNF-YC9*) and *RESPONSIVE TO DESICCATION 26* (*PtoRD26*), to regulate an ABA-dependent salt-stress response signaling. We reveal that the salt-inducible *PtoRD26* is dependent on ABA signaling. We demonstrate that ABA or salt drives PtoNF-YC9 shuttling into the nucleus where it interacts with SRMT, resulting in the rapid expression of *PtoRD26* which in turn directly regulates *SRMT*. This positive feedback loop of *SRMT*-*PtoRD26* can rapidly amplify salt-stress signaling. Interference with either component of this regulatory module reduces the salt tolerance of this triploid poplar.

**Conclusion:**

Our findings reveal a novel ABA-dependent salt-responsive mechanism, which is mediated by the PtoNF-YC9-SRMT-PtoRD26 module that confers salt tolerance to this triploid poplar. These genes may therefore also serve as potential and important modification targets in breeding programs.

**Supplementary Information:**

The online version contains supplementary material available at 10.1186/s13059-022-02718-7.

## Background

As sessile organisms, plants have to cope with various abiotic stresses. How plants sense and respond to these stress signals is one of the fundamental biological questions that needs answering in order to elucidate adaptive mechanisms to adverse environments [1]. High salinity is one of the main abiotic stress factors. It causes ionic, osmotic, oxidative, and other secondary stresses [2, 3] that inhibit photosynthesis, reduce vegetative growth, block respiration, and disrupt metabolism [4–6]. The cellular responses of plants to salt stress include an early sensing phase, a stop phase in which the plant growth rate decreases, a quiescent phase where the growth rate stays low, and a growth recovery phase in which the growth rate partly recovers [7]. These processes require changes of abscisic acid (ABA) concentrations and ABA-dependent transcription networks [8]. ABA signaling is therefore indispensable for plants to rapidly trans-shift from growth to defense. ABA molecules are perceived by co-receptors composed of PYRABACTIN RESISTANCE1 (PYR1)/PYR1-LIKE (PYL)/REGULATORY COMPONENTS OF ABA RECEPTORS (RCAR) proteins and PROTEIN PHOSPHATASE 2C (PP2C) family members [9–13]. Further activation of SNF1-RELATED PROTEIN KINASE 2 (SnRK2) mediates the direct phosphorylation and activation of downstream transcription factors [14].

A complex hierarchy among transcription factors and a comprehensive regulatory network of ABA pathways have been revealed and built into the model plant *Arabidopsis* [15]. A well-known NAC (NO APICAL MERISTEM, ARABIDOPSIS TRANSCRIPTION ACTIVATION FACTOR AND CUP-SHAPED COTYLEDON) gene, *RD26* (or else named *NAC072*) in *Arabidopsis*, and its homologs in various species participate in the metabolic reprogramming of plant senescence [16–18], and in early responses to series of environmental stresses, including drought, high salinity, low temperature, and hypoxia [19–25]. These *RD26* homologs have been shown to respond to ABA, jasmonic acid (JA), and brassinosteroid (BR) and act strongly depending on these phytohormone-mediated signaling pathways [21, 22, 26]. For example, this gene may mediate crosstalk between BR-dependent growth and the ABA-dependent drought response [22, 23] to drive the flow of matter and energy from growth to the stress response, resulting in a stunted growth phenotype of the gene-over-expressed plants [22]. The RD26 protein is stabilized through phosphorylation by BIN2 in the presence of ABA and dehydration, but this process is inhibited by ABI1 in the growth phase [23]. In *Populus* species (poplars), RD26 is regulated at both the transcript and protein level, regardless of species and drought conditions, and thus serves as a critical and universal drought marker [25]. In addition, a negative feedback loop caused by an E3 ubiquitin ligase gene *PUB79* and the transcription repressor WRKY77, can rapidly remove transcriptional inhibition of *RD26* to provoke an ABA-dependent salt-stress response in poplar [24, 27].

The Chinese white poplar (*P. × tomentosa* Carr. clone 741) is one of the most important fast-growing forestry trees used for timber, landscaping, and farmland shelter belting in northern China [28–30]. This artificial allotriploid is superior to diploid poplars in growth, wood quality, and stress resistance, especially where high saline tolerance is required [31, 32]. High efficiency of exogenous gene transformation and genome editing enables this triploid poplar to be ideal for creating superior tree cultivars [32–34] and as a model research tool. However, the complex and elusive triploid genome sequences of this poplar severely hamper investigations on underlying molecular mechanisms for special traits because it is difficult to exclude the effects of allelic sequence variations. In our present study, we firstly report haplotype-resolved genome sequences of this triploid poplar. Using a well-assembled genome sequence and the following transcriptome and functional analyses, we revealed that a novel salt-stress-responsive mechanism, which is composed of *PtoNF-YC9*, *SRMT*, and *PtoRD26* together provoke and amplify the ABA-dependent salt response in this poplar. In fact, NF-YC9 participates in the ABA pathway of *Arabidopsis* through interacting with RGL2 to regulate seed germination by integrating gibberellin (GA) and ABA signaling [35], ABF3 and ABF4 to mediate drought-accelerated flowering [36], and ABI5 to regulate salt and drought responses [37]. *SRMT* also encodes an R2R3 MYB transcription factor and is involved in acclimation by poplar to dehydration and cold during autumnal bud development [38]. Here we found that the ABA or salt drives the translocation of PtoNF-YC9 from cytoplasm to nucleus and then interacts with SRMT to activate *PtoRD26* expression. *SRMT* and *PtoRD26* are mutually and positively regulated to amplify salt-stress signaling, resulting in increased salt tolerance of this triploid poplar. We found high peptide sequence similarity and no variations of the critical regulatory elements among the alleles of each of three genes based on well-assembled genome sequences. Our findings therefore overcome the genomic limitation of the triploid poplar to an examination of any underlying molecular mechanisms of the special trait; they also provide a solid basis for creating other new cultivars with a broad range of adaptations.

## Results

### Haplotype-resolved genome sequence of the triploid poplar and the identification of the high PtoRD26 expression during salt stress

**Fig. 1:**
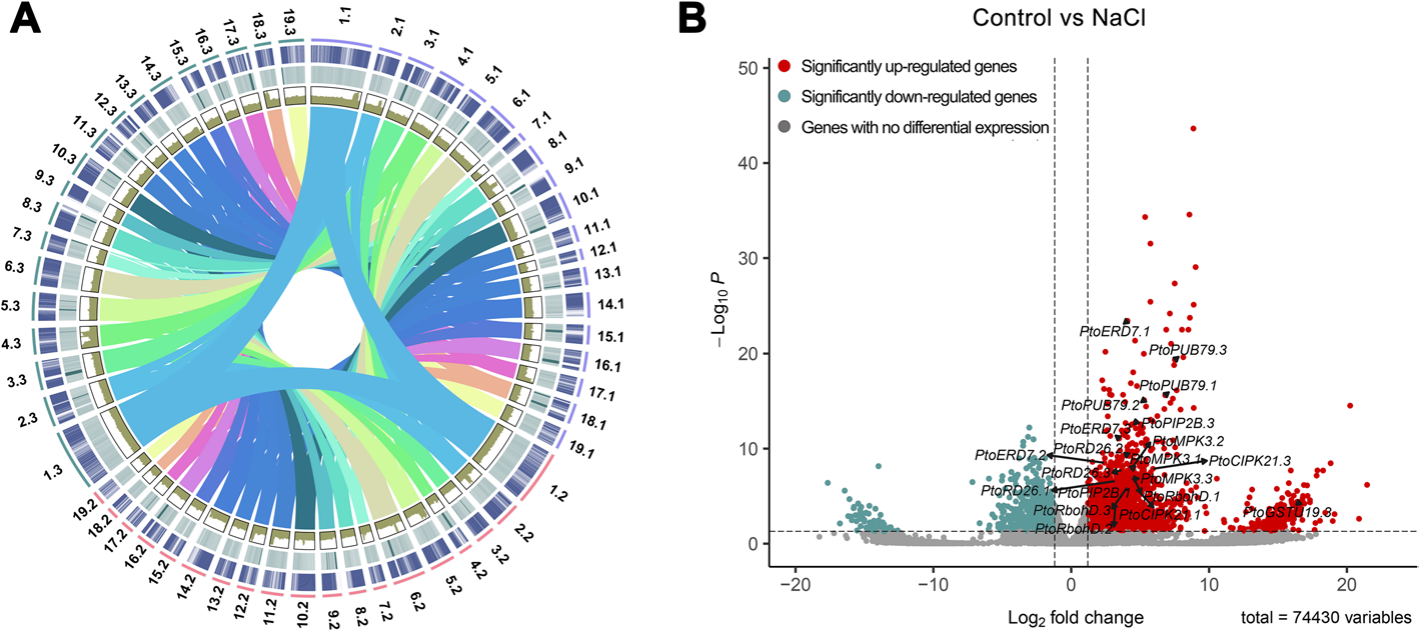
Chinese white poplar genome assembly, genomic features, and RNA-seq analysis after salt treatment. **A** The circo diagram of Chinese white poplar genome, which from outer to inner separately represented haplotype, chromosome, gene density, transposable element density, read mapping depth, and colinear links. **B** The volcano plot shows the differentially expressed genes (DEGs) in the RNA-seq analysis of triploid Chinese white poplar after 300 mM NaCl treatment compared to the double-distilled water control. The DEGs upregulated were represented by red dots, the downregulated ones were indicated by blue dots. The gray dots represented the genes without significant changes. The black dot represents typical salt-stress-responsive genes, including *PtoERD7.1* (*P.x_tomentosa28552*), *PtoERD7.2* (*P.x_tomentosa31148*), *PtoERD7.3* (*P.x_tomentosa29872*), *PtoPUB79.1* (*P.x_tomentosa72982*), *PtoPUB79.2* (*P.x_tomentosa70753*), *PtoPUB79.3* (*P.x_tomentosa71860*), *PtoPIP2B.1* (*P.x_tomentosa61890*), *PtoPIP2B.3* (*P.x_tomentosa59726*), *PtoMPK3.1* (*P.x_tomentosa13975*), *PtoMPK3.2* (*P.x_tomentosa17411*), *PtoMPK3.3* (*P.x_tomentosa10444*), *PtoRbohD.1* (*P.x_tomentosa56106*), *PtoRbohD.2* (*P.x_tomentosa57812*), *PtoRbohD.3* (*P.x_tomentosa54409*), *PtoRD26.1* (*P.x_tomentosa47952*), *PtoRD26.2* (*P.x_tomentosa46818*), *PtoRD26.3* (*P.x_tomentosa48940*), *PtoCIPK21.1* (*P.x_tomentosa44463*), *PtoCIPK21.3* (*P.x_tomentosa45540*), *PtoGSTU19.3* (*P.x_tomentosa09261*)

By combining 86.64 Gb high-quality PacBio HiFi reads and 198.65 Gb chromosome conformation capture (Hi-C) sequencing data, we generated a haplotype-resolved chromosome-level assembly of this triploid poplar with an estimated genome size of 1.58 Gb (Table 1, Additional file 1: Fig. S1 and Additional file 2: Table S1). The final assembly contains 1.07 Gb in 57 superscaffolds and 441.80 Mb of unplaced contigs, representing all 57 chromosomes comprising 19 homologous groups with three allelic chromosomes in each group (Fig. 1A). Since *P. alba* var. *pyramidalis* is one of the parents of this allotriploid poplar, we assigned the chromosomes of each homologous group into haplotypes I (“*.1”), III (“*.3”), and II (“*.2”) in descending order of similarity to the genome of *P. alba* var. *pyramidalis* [39]. The HiFi long reads and Illumina short reads were then mapped to the assembly; the results show that most genomic regions exhibited a similar pattern of the read depth distribution. Additionally, Benchmarking Universal Single-Copy Orthologs (BUSCO) analysis showed that 94.80%, 91.70%, and 91.70% of conserved genes can be completely covered by the three monoploid genomes, respectively, containing a total of 97.20% complete BUSCO genes (Table 1). Further analysis showed that the alleles from the three haplotypes had a high degree of sequence identity in the coding sequence (CDS), protein, and 1500 bp-length promoter sequence, respectively (Additional file 1: Fig. S2). Taken together, these results confirm that our assembly resolved the haplotypes of the triploid poplar very well.

**Table 1:** Assembly and annotation features of triploid Chinese white poplar genomes

**Genome features**	
Number of contigs	17,616
Total size of contigs	1,499,697,831 bp
Longest contig	8,084,294 bp
Mean contig size	85,132 bp
N50 contig length	1,209,510 bp
Contig GC content	36.85%
Number of superscaffolds	57
Total size of superscaffolds	1,064,994,437 bp
N50 superscaffold length	19,669,985 bp
Reads mapping ratio	99.35%
Reads coverage ratio	93.36%
Genome BUSCO (complete)	97.20%
**Gene models**	
Number of gene models	91,397
Mean coding sequence length	1,371.51 bp
Mean number of exon per gene	5.94
Mean exon length	309.89 bp
Mean intron length	385.55 bp
Protein BUSCO (complete)	98.80%
**Non-coding RNAs**	
Number of tRNAs	26,773
Number of rRNAs	23,652
Number of snRNAs	1,372
Number of miRNAs	2,336

Based on a combination of homology, de novo and transcriptome-based gene prediction, we identified a total of 91,397 protein-coding genes in the genome, 98.95% of which were functionally annotated *via* searches of publicly available databases. To examine transcriptome changes of this triploid poplar in early response to salt stress, the 45-day-old triploid poplars in soil were watered with a NaCl solution; a control received water only. After 4 h, the leaf at the 3rd node was detached for RNA sequencing [40]. There were in total 4809 differentially expressed genes (DEGs), including 3801 upregulated and 1008 downregulated genes (Fig. 1B, Additional file 1: Fig. S3 and Additional file 3: Table S2). We found that the *PtoRD26.1* (*P.x_tomentosa47952*) and its alleles *PtoRD26.2* (*P.x_tomentosa46818*) and *PtoRD26.3* (*P.x_tomentosa48940*) were significantly upregulated after salt treatment (Fig. 1B and Additional file 3: Table S2). This transcription factor was found to represent a critical and universal drought marker for poplars [25] and was therefore selected for further studies.

### PtoRD26 positively regulates poplar tolerance to salt stress

**Fig. 2:**
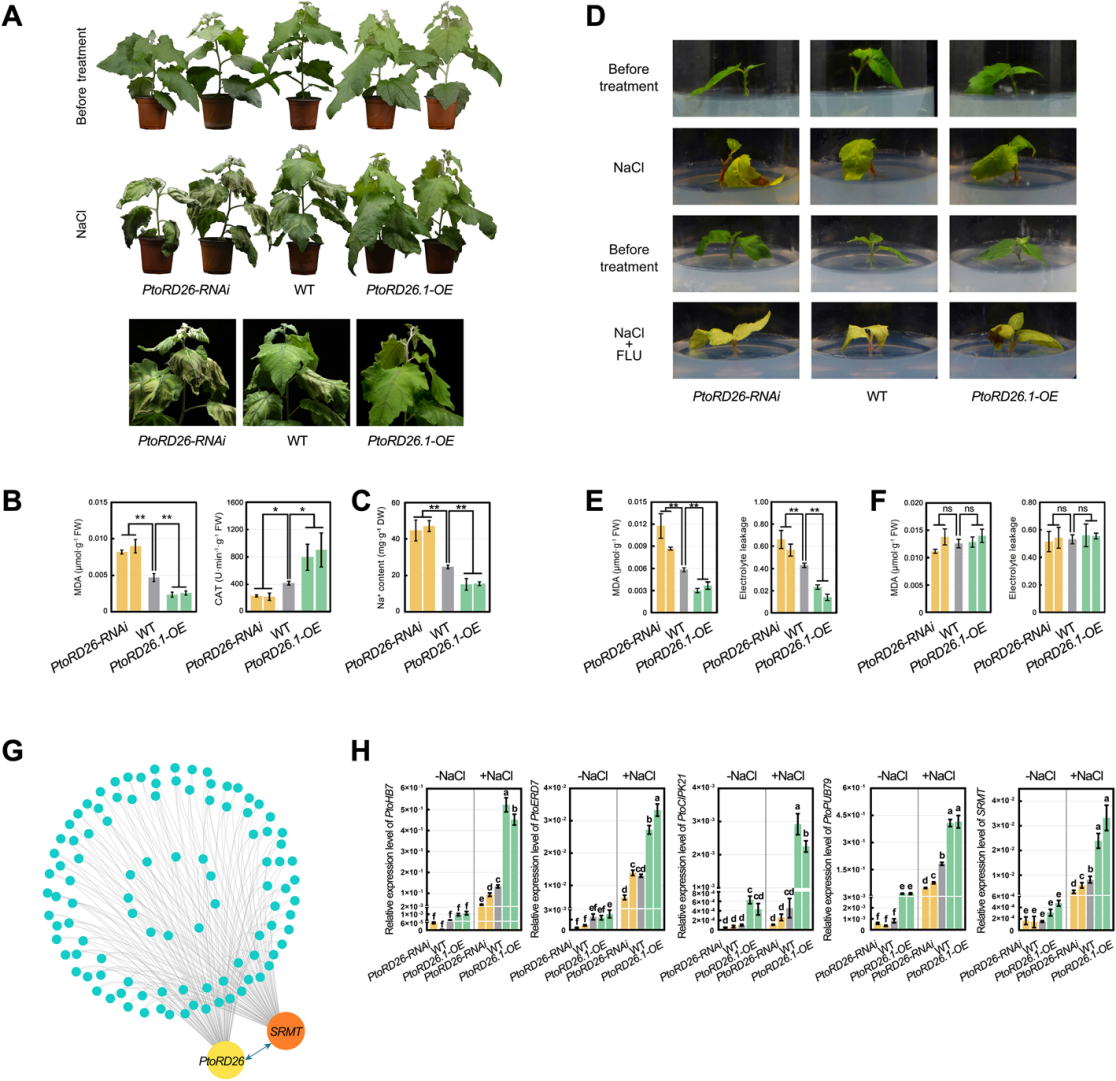
Positive regulation of poplar tolerance to salt stress by *PtoRD26* dependent on ABA signaling. **A** The salt tolerance of 45-day-old *PtoRD26-RNAi* (L2 and L6), WT and *PtoRD26.1-OE* (L1 and L17) poplars in the soil treated by irrigating with 300 mM NaCl solution. **B** The malondialdehyde (MDA) content and activity of catalase (CAT) in leaves of different poplar genotypes (**A**) after salt treatment. **C** The Na^+^ content of poplar leaves (**A**) after salt treatment. **D** The salt tolerance of *PtoRD26-RNAi*, WT, and *PtoRD26.1-OE* cuttings in the WPM solid medium supplemented with 150 mM NaCl or 150 mM NaCl plus 10 μM FLU. **E** The MDA content and EL of the poplar cuttings (**D**) grown in WPM medium with NaCl. **F** The MDA content and EL of the poplar cuttings (**D**) in WPM medium with both NaCl and FLU. The mean ± s.d. of all data from at least three biological replicates is shown. Asterisks indicate statistically significant differences (two-sided Student’s *t* test, *P < 0.05, **P < 0.01, ns = not significant). **G** Co-expression network of stress-related genes constructed from data of 62 poplar transcriptomes. We generated transcriptomic data of 17 samples from triploid poplar and 9 samples from *P. abla* var. *pyramidalis*, and collected 36 other transcriptomic data of *P. abla* var. *pyramidalis* that have been released in NCBI. *PtoRD26* and a R2R3 MYB transcription factor named *SRMT* showing co-expression relationships with stress-associated genes. **H** The qPCR analysis determined the expression level of *PtoHB7*, *PtoERD7*, *PtoCIPK21*, *PtoPUB79*, and *SRMT* in the *PtoRD26* overexpression, RNAi, and WT lines with or without salt treatment for 4 h, respectively. The qPCR primers of each gene can detect all alleles simultaneously. The expression level of *SRMT* was enhanced in the *PtoRD26.1* overexpression poplars but reduced in the RNAi lines compared to the WT. Letters above bars represent statistically significant differences between groups (*P* < 0.05) as determined by one-way ANOVA Duncan’s test

*PtoRD26* is predominantly expressed in mature leaf, petiole, and root and is induced by salt stress in these tissues (Additional file 1: Fig. S4). To determine the function of *PtoRD26* in response to salt stress, we generated the *PtoRD26.1* overexpression (*PtoRD26.1-OE*) and the RNAi knocking-down (*PtoRD26-RNAi*) poplars that showed no significant differences in plant height under normal conditions (Fig. 2A and Additional file 1: Fig. S5). After salt stress, the *PtoRD26-RNAi* plants showed severe necrosis in shoots and leaves, whereas the wild-type (WT) leaves were only mildly withered, and the leaves of *PtoRD26.1-OE* lines just drooped (Fig. 2A). Salt stress causes excessive Na^+^ accumulation in leaves resulting in cationic toxicity [41], and increased ROS (for example H_2_O_2_), which mediates cellular oxidative damage leading to enhanced malondialdehyde (MDA) in cells [42]. Na^+^ content in leaves, MDA content, and the capability of ROS scavenging by catalase (CAT) can therefore reflect the degree of a plant’s tolerance and stress to salt. As shown in Fig. 2B, C, the physiological indicators Na^+^ and MDA were highest in *PtoRD26*-*RNAi* poplars and lowest in the *PtoRD26.1-OE* lines. In addition, the enzymatic activity of CAT increased in *PtoRD26.1-OE* poplars but decreased in *PtoRD26*-*RNAi* plants. These results indicate that the *PtoRD26*-*RNAi* poplars were severely damaged by salt stress, while *PtoRD26.1-OE* poplars were only mildly stressed. *PtoRD26* thus positively regulates salt tolerance in the triploid poplar, which is consistent with previous investigations [24, 27]. In addition, we found one nonsynonymous variation among three *PtoRD26* alleles in the NAM domain region with respect to three sets of well-assembled genome sequences (Additional file 1: Fig. S6). These regions in fact share high sequence similarity between this triploid and *P. alba* var. *pyramidalis*.

In *Arabidopsis*, *RD26* is involved in ABA-dependent stress signaling [21] while its homolog in poplar is modulated by WRKY77, a repressor of ABA signaling [24]. To ascertain whether *PtoRD26* in this triploid poplar regulates salt tolerance through ABA signaling, we blocked the accumulation of ABA triggered by salt stress with fluridone (FLU), an ABA biosynthesis inhibitor. In the solid medium containing NaCl, the phenotypes of *PtoRD26-RNAi*, WT, and *PtoRD26.1-OE* cuttings were consistent with previous observations in soil (Fig. 2D). However, the degree of stress among these cuttings became similar when they were supplemented with FLU; even the enhanced salt tolerance of the *PtoRD26.1-OE* lines disappeared (Fig. 2D). Two physiological indicators of plasma membrane damage [42], the rates of electrolyte leakage (EL), and the MDA content also supported our observations (Fig. 2E,F). The salt tolerance of this triploid poplar *PtoRD26* is thus regulated through the accumulation of ABA.

To reveal the transcriptional regulation network of *PtoRD26* in response to salt stress, we analyzed the co-expression genes of *PtoRD26* based on the RNA-seq data from 17 and 45 cuttings for this triploid and *P. alba* var. *pyramidalis*, respectively (Additional file 4: Tables S3). In total, 2428 genes had similar patterns of expression, including 303 genes involved in drought, salt stress, and the ABA pathway (Additional files 5, 6 and 7: Tables S4-S6). We found that an R2R3 MYB transcription factor encoding gene was induced by drought, ABA, and salt stress (Additional file 1: Figs. S7 and S8) and co-expressed with *PtoRD26*, we thus named it *SALT RESPONSE MYB TRANSCRIPTION FACTOR* (*SRMT*) and only two alleles were identified in the triploid genome (*SRMT.2*, *P.x_tomentosa70538*; *SRMT.3*, *P.x_tomentosa71644*). Moreover, 103 of the 303 stress-associated genes had a co-expression relationship both with *PtoRD26* and *SRMT* (Fig. 2G). The expression levels of *SRMT* and four representative NaCl-inducible genes from our RNA-seq data, including *PtoHB7*, *PtoERD7*, *PtoCIPK21*, and *PtoPUB79*, were upregulated in *PtoRD26.1-OE* poplars but downregulated in *PtoRD26-RNAi* poplars under highly saline condition (Fig. 2H). There might therefore be a transcriptional regulation on *SRMT* mediated by *PtoRD26* in responses of this triploid poplar to salt stress.

### PtoRD26 directly and positively regulates SRMT transcription

**Fig. 3:**
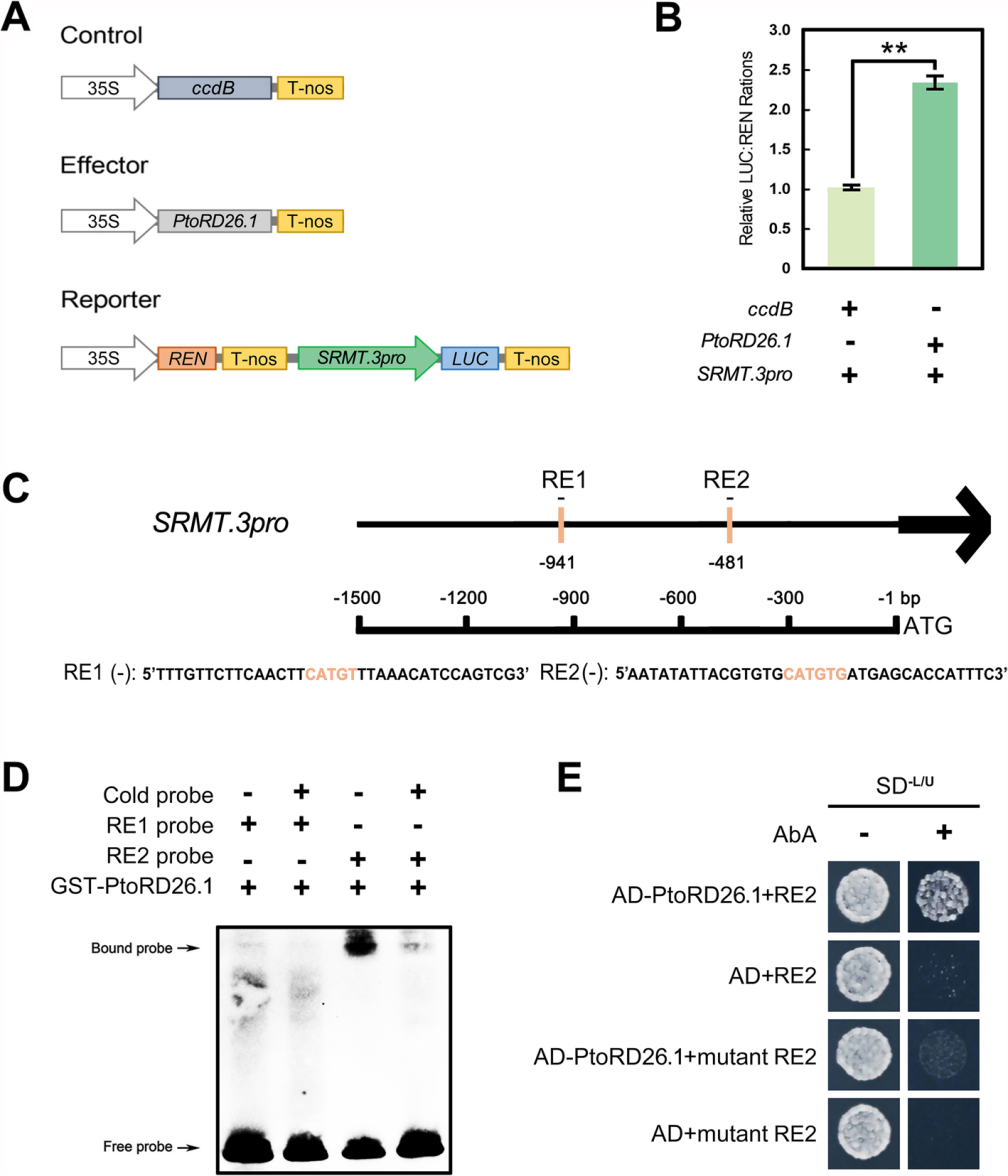
*PtoRD26* directly and positively regulates *SRMT* expression. **A** The constructs of the effector and reporter in the dual-luciferase assay. The pCXSN empty vector contains a *ccdB* gene that is a negative selection marker to eliminate the self-ligation background after transformation. **B***PtoRD26.1* enhanced fluorescence intensity of *LUC* driven by the *SRMT.3* promoter (*SRMT.3pro*) compared to the control. The mean ± s.d. of three biological replicates is shown. Asterisks indicate statistically significant differences (two-sided Student’s *t* test, **P < 0.01). **C** The distribution of the PtoRD26 binding element (RE1 and RE2) in the *SRMT.3* promoter. **D** EMSA indicating that PtoRD26.1 can bind competitively by cold probes to RE2, but not to RE1. **E** The Y1H assay verifying the binding of PtoRD26.1 to RE2 in the *SRMT.3pro*

To prove whether PtoRD26 targets to *SRMT*, a dual-luciferase (LUC) assay showed that PtoRD26.1, PtoRD26.2, and PtoRD26.3 obviously elevated the transcriptional activity of the *SRMT.3*’s promoter (Fig. 3A,B, Additional file 1: Fig. S9). In addition, there are two potential binding elements (RE) of PtoRD26 [22] in the negative strand of the *SRMT* promoter, located at −941 bp (RE1) and −481 bp (RE2), respectively (Fig. 3C). An electrophoretic mobility shift assay (EMSA) and a yeast one-hybrid (Y1H) assay demonstrated that PtoRD26 only binds to the RE2, but not the RE1 (Fig. 3D,E). These results indicate that PtoRD26 directly and positively regulates *SRMT* expression *via* binding to RE2 in the *SRMT* promoter. Notably, we found no nonsynonymous variations in the R2R3 domain of the two *SRMT* alleles in the triploid genome (Additional file 1: Fig. S10), nor variations within the critical binding elements of PtoRD26 in their promoters and *P. alba* var. *pyramidalis* [39] (Additional file 1: Fig. S11).

### SRMT positively regulates salt tolerance dependent on ABA

**Fig. 4:**
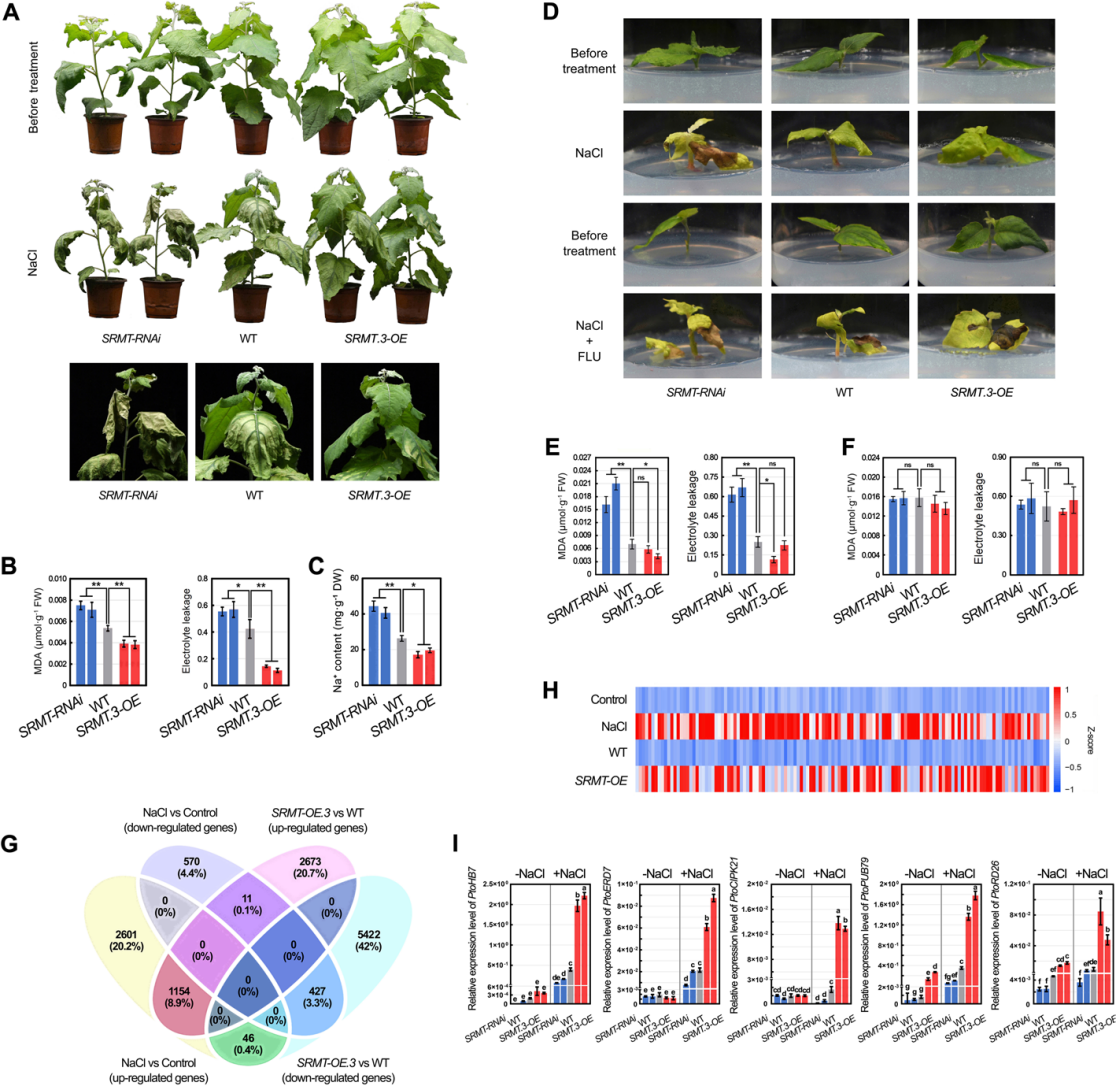
Positive regulation of poplar tolerance to salt stress by *SRMT* is dependent on the ABA signaling pathway. **A** The phenotype of *SRMT-RNAi* (L4 and L29), WT, and *SRMT-OE* (L11 and L13) poplars in soil after salt treatment through watering with 300 mM NaCl solution, indicating increased tolerance to salt stress in *SRMT.3* overexpressing lines, but decreased salt tolerance in *SRMT*-RNAi lines. **B** The MDA content and EL in leaves of three poplar genotypes after salt treatment. **C** The Na^+^ content in the leaves detached from the *SRMT-RNAi*, WT, and *SRMT.3-OE* poplars after salt treatment. **D** The salt tolerance of *SRMT-RNAi*, WT, and *SRMT.3-OE* planted in the WPM solid medium supplemented with 150 mM NaCl, or both 150 mM NaCl and 10 μM fluridone (FLU). The enhanced salt tolerance of transgenic poplars by overexpressing *SRMT.3* was eliminated by adding FLU to the medium. **E** The MDA content and EL in the RNAi, overexpression, and WT lines planted in the WPM solid medium containing 150 mM NaCl indicates results consistent with observations of phenotypes. **F** The MDA content and EL in different poplar genotypes planted in the WPM solid medium containing 150 mM NaCl and 10 μM FLU were not significantly different. The mean ± s.d. of all data from at least three biological replicates is shown. Asterisks indicate statistically significant differences (two-sided Student’s *t* test, *P < 0.05, **P < 0.01, ns = not significant). **G** Venn diagram indicating the DEGs in the *SMRT.3* overexpression poplar compared to WT poplars under identical normal condition, and WT treated with 300 mM NaCl compared to the controls (WT treated by ddH_2_O). **H** Heatmap indicating the expression levels of 131 genes of the top 700 upregulated DEGs associated with ABA, drought, and salt stress, etc. in the *SRMT.3* overexpression poplar compared to WT. **I** The qPCR detected expression levels of *PtoHB7*, *PtoERD7*, *PtoCIPK21*, *PtoPUB79*, and *PtoRD26*, which were upregulated in *SRMT.3-OE* lines and downregulated in *SRMT-RNAi* lines with or without salt treatment for 4 h, respectively. Letters above bars represent statistically significant differences between groups (*P* < 0.05) as determined by one-way ANOVA Duncan’s test

To clarify the function of *SRMT* in the salt stress response, we generated the transgenic poplars: overexpressing *SRMT.3* (*SRMT.3-OE*) and knocking-down (*SRMT-RNAi*) that did not differ significantly in growth under normal condition (Fig. 4A and Additional file 1: Fig. S12C). However, the leaves, especially the mature and old leaves, of *SRMT-RNAi* poplars were crimped below the 5th node, but *SRMT.3-OE* leaves were not (Additional file 1: Fig. S12D). After watering the 45-day-old poplars in soil with 300 mM NaCl solution, the leaf edge of *SRMT-RNAi* poplars showed some withered and dehydration after just 1 day. Five days post treatment, *SRMT-RNAi* poplars suffered a serious withering of leaves compared to WT. In comparison, the leaves of the *SRMT.3-OE* plants just drooped and were only slightly desiccated along their edges (Fig. 4A). These observations were corroborated by measurements of Na^+^, MDA, and EL in these stressed materials (Fig. 4B,C). These results demonstrate the positive regulation of *SRMT* on poplar regarding their tolerance to salt stress. This regulatory process might be involved in ABA because *SRMT* can be significantly induced by ABA. To test this, cuttings of *SRMT-RNAi*, WT, and *SRMT.3-OE* poplars were transplanted into the medium with NaCl, where their different tolerances to salt could be eliminated by FLU supplements (Fig. 4D–F), which is consistent with *PtoRD26.1-OE* poplar. We also found that *SRMT.3-OE* cuttings in the medium containing ABA, which is known to inhibit plant growth [24], were indeed severely inhibited in their growth, whereas the growth of *SRMT-RNAi* cuttings did not differ significantly from WT in their level of inhibition (Additional file 1: Fig. S13A). The dry weights of these ABA-treated poplar cuttings also indicated that the *SRMT-RNAi* lines had accumulated more biomass than the *SRMT.3-OE* (Additional file 1: Fig. S13B). The *SRMT*’s positive regulation of salt tolerance in poplar is thus dependent on ABA.

We subsequently used RNA-seq analysis to examine the transcriptome changes caused by *SRMT*. Compared to WT, there were 3838 upregulated and 5895 downregulated DEGs in the *SRMT.3*-*OE* poplar (Fig. 4G and Additional file 8: Table S7). Among these genes, there were 1154 upregulated and 427 downregulated genes that were also induced and repressed by NaCl treatment (Fig. 4G, Additional files 9 and 10: Table S8 and S9), respectively. Of the first 700 upregulated genes, there were in total 131 genes associated with ABA, salt, drought, and osmotic stresses (Fig. 4H and Additional file 11: Table S10), including *PtoHB7*, *PtoERD7*, *PtoCIPK21*, *PtoPUB79*, and *PtoRD26.* These representative genes exhibited increased levels of expression in the *SRMT.3-OE* poplars but decreased levels in the *SRMT-RNAi* after salt treatment (Fig. 4I). These results indicate that *SRMT* mediates transcriptional reprogramming in response to salt stress.

### SRMT regulates PtoRD26 response to salt stress and ABA

**Fig. 5:**
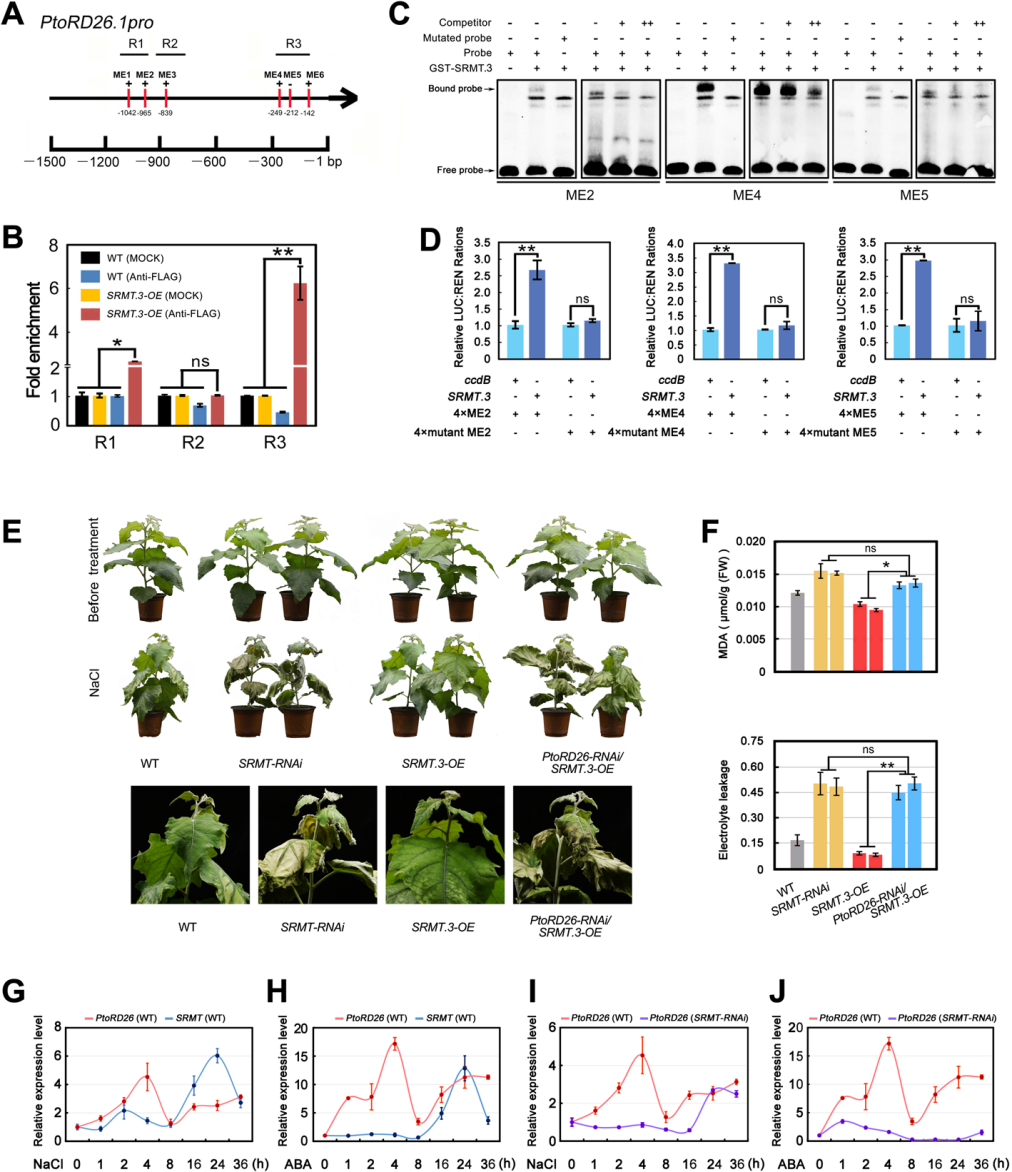
*SRMT* positively and directly regulates *PtoRD26* which is epistatic to *SRMT*. Temporal expression patterns of *PtoRD26* and *SRMT* in response to salt stress and ABA treatment. **A** The distribution of SRMT binding elements (ME) in the 1.5 kb promoter region of *PtoRD26.1*, named ME1-ME6 (red lines). The promoter regions (R1–R3) show the amplified fragments of ChIP-qPCR. **B** ChIP-qPCR detection of SRMT binding regions in the *PtoRD26.1* promoter in vivo. There were significant enrichments in the R1 and R3 regions. **C** EMSA shows the GST-SRMT.3-fused protein binds to ME2, ME4, and ME5 in vitro. These bindings could be competitive by cold probes; the mutant probe could not be bound by the GST-SRMT.3-fused protein. **D** Dual-luciferase assay indicating that SRMT.3 bound to fourfold repeats of ME2, ME4 and ME5 to activate the expression of *LUC* gene in tobacco leaves. However, the mutated ME2, ME4 and ME5 could not be bound by SRMT.3 and thus *LUC* could not be activated. **E***PtoRD26* was epistatic to *SRMT*. This genetic relationship was determined by assessing the salt tolerance of the double transgenic poplars (L3 and L5 of *PtoRD26-RNAi/SRMT.3-OE*) in which *PtoRD26* was knocked down in the background of *SRMT.3-OE*-L11, in comparison to *PtoRD26-RNAi* and *SRMT.3* overexpression poplars. **F** The MDA content and EL in *PtoRD26-RNAi* and *SRMT.3-OE* and *PtoRD26-RNAi/SRMT.3-OE* poplar leaves after salt treatment. The means ± s.d. of all data from at least three biological replicates are shown. Asterisks indicate statistically significant differences (two-sided Student’s *t* test, **P < 0.01, *P < 0.05, ns = not significant). **G** The temporal expression levels of *PtoRD26* and *SRMT* at 0, 1, 2, 4, 8, 16, 24, and 36 h after NaCl treatment. **H** The temporal expression levels of *PtoRD26* and *SRMT* at 0, 1, 2, 4, 8, 16, 24, and 36 h after ABA treatment. **I** The comparison of the temporal expression pattern of *PtoRD26* in responsive to NaCl treatment in the background of WT and *SRMT-RNAi* poplars, respectively. **J** The comparison of the temporal expression pattern of *PtoRD26* in response to ABA treatment in the background of WT and *SRMT-RNAi* poplars

That the expression level of *PtoRD26* could be upregulated by SRMT, as demonstrated by qPCR analysis (Fig. 4I) and a dual-LUC assay (Additional file 1: Fig. S14), suggests that SRMT might directly regulate *PtoRD26*. AtMYB112, a homolog of SRMT in *Arabidopsis*, binds to an 8-bp DNA fragment containing the core sequence (A/T/G)(A/C)CC(A/T)(A/G/T)(A/C)(T/C) [43] that might be also bound by SRMT. There were six potential SRMT binding elements (ME1-ME6) in the *PtoRD26.1* promoter (Fig. 5A), and the ChIP-qPCR analysis indicated that the promoter region R1 harboring ME1 and ME2, and the region R3 containing ME4, ME5, and ME6 could be enriched, but there was no significant enrichment of the region R2 (Fig. 5B). A subsequent EMSA demonstrated that the GST-SRMT.3 recombination protein bound to ME2, ME5, and especially to ME4 (Fig. 5C and Additional file 1: Fig. S15). We also constructed three vectors composed of fourfold repeats of the ME2, ME4, and ME5 and a 35S minimal promoter to drive the *LUC* reporter gene, for another dual-LUC assay. This assay indicated that while SRMT.3 could significantly increase *LUC* expression compared to the controls, the mutant MEs could not (Fig. 5D). These results demonstrate that *SRMT* positively regulates *PtoRD26* expression through binding to ME2, ME4, and ME5 in its promoter. It should be noted that we also examined allelic variations of *PtoRD26* genes at promoter regions on three sets of the genome sequences, and we found no variation in these critical *cis*-elements within this triploid, nor between it and *P. alba* var. *pyramidalis* [39] (Additional file 1: Fig. S16).

To investigate the genetic relationship between *PtoRD26* and *SRMT* in poplar’s tolerance to salt stress, we knocked down *PtoRD26* by RNAi in the *SRMT.3-OE-*L11 background poplar (*PtoRD26-RNAi/SRMT.3-OE*) (Additional file 1: Fig. S17). The results show that salt tolerance in the phenotype of *SRMT.3-OE-*L11 poplars was removed by knocking down *PtoRD26*, and became similar to that of *PtoRD26-RNAi* poplars (Fig. 5E,F), indicating that *SRMT* functioning requires *PtoRD26* and that *PtoRD26* is epistatic to *SRMT.* To reveal the regulatory mechanism of *SRMT* to *PtoRD26* transcription in poplar’s response to salt stress, we examined the temporal expression patterns of *PtoRD26* and *SRMT* in WT poplars under NaCl treatment. NaCl treatment rapidly increased *PtoRD26* expression to a maximum at 4 h post treatment (Fig. 5G). *SRMT* was slightly upregulated at 2 h after treatment but decreased, and it was significantly induced by 8 h post treatment and reached a maximum after 24 h (Fig. 5G). Because *PtoRD26* and *SRMT* regulation of salt tolerance is dependent on ABA, the temporal expression patterns of these two genes on exposure to ABA were also constructed and gave similar results to the NaCl treatment (Fig. 5H). These results suggest that *PtoRD26* appears to be upstream of *SRMT*, but overexpressing *SRMT* could not rescue the salt sensitivity of *PtoRD26*-*RNAi* plants (Fig. 5E). To explore this relationship, we examined the expression of *PtoRD26* in the *SRMT-RNAi* plants under NaCl and ABA treatments. This inducible *PtoRD26* vanished in *SRMT*-*RNAi* poplars in the first 16 h post NaCl treatment and then recovered (Fig. 5I), whereas the ABA-inducible *PtoRD26* totally disappeared within the 36 h of the treatment (Fig. 5J). *PtoRD26* transcription in response to salt stress (within the first 16 h) therefore requires *SRMT*-mediated ABA signaling. In addition, we noted that the expression of *PtoRD26* in *SRMT*-*RNAi* poplars recovered after 24 h of NaCl treatment (Fig. 5I). This recovery seemed not to rely on ABA signaling because it was not consistent with the results of ABA treatment (Fig. 5J). A previous investigation reported that a homolog of *SRMT* positively regulates JA signaling [44] and that JA levels rise in the recovery stage of the cellular response to salt stress [7]. We therefore wondered whether JA mediated this recovery of *PtoRD26* expression by *SRMT*. To explore this, we examined the expression of *PtoRD26* exposure to methyl jasmonate (MeJA), a JA derivative. The *PtoRD26* expression started to increase significantly from 24 h post MeJA treatment, both in WT and *SRMT-RNAi* poplars (Additional file 1: Fig. S18). These findings indicate that *PtoRD26* responsiveness to MeJA is not dependent on *SRMT* and suggests that the recovered *PtoRD26* expression might be mediated by JA signaling.

### ABA and salt drive PtoNF-YC9 to be transferred from the cytoplasm to the nucleus where it physically interacts with SRMT

**Fig. 6.**
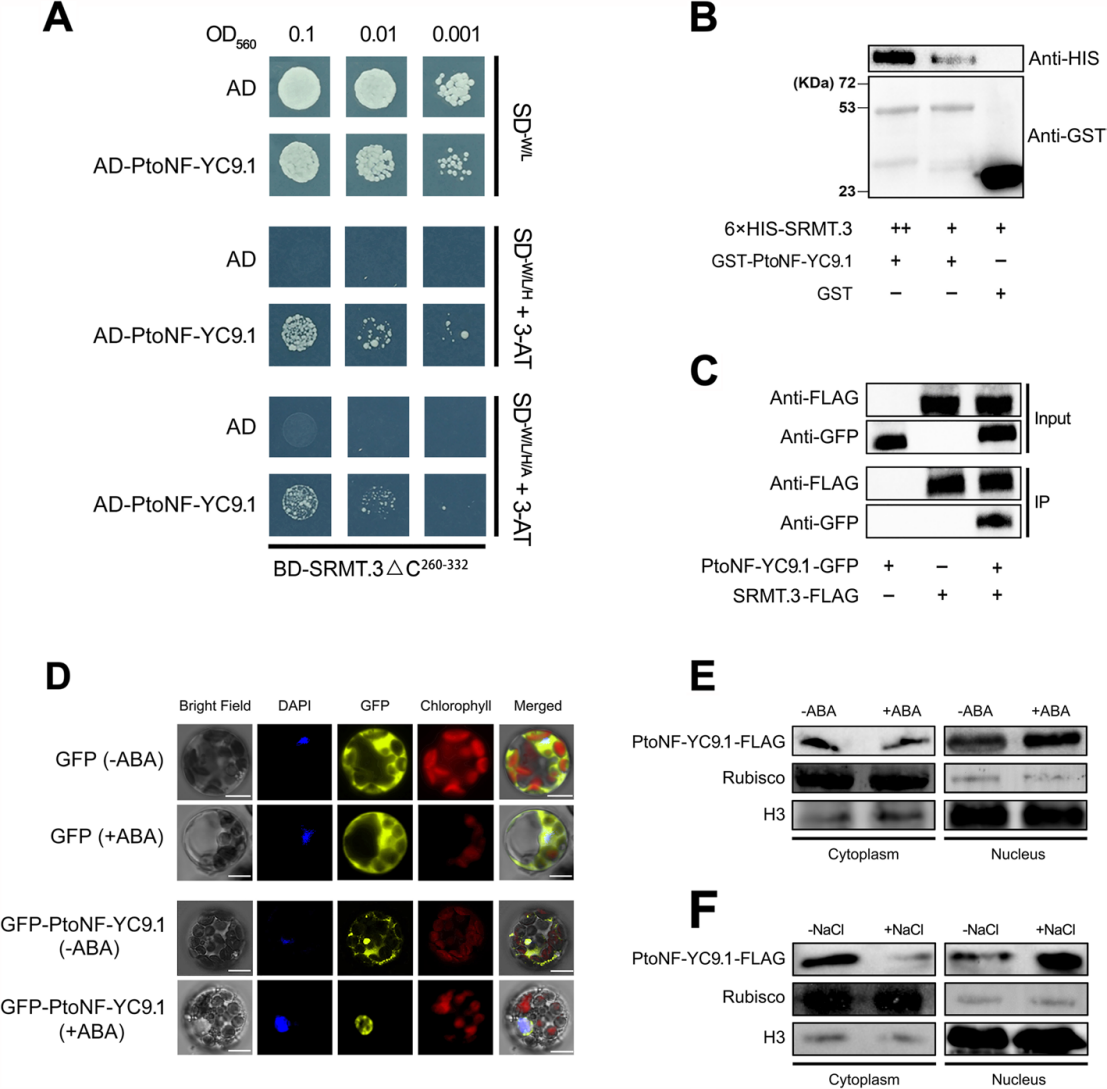
SRMT interacts with PtoNF-YC9 in vitro and in vivo, and ABA signaling mediates shuttling PtoNF-YC9 from cytoplasm to nucleus. **A** The physical interaction between SRMT.3 and PtoNF-YC9.1 was investigated by Y2H assay. **B** The pull-down assay showed GST-PtoNF-YC9.1 to interact with the 6 × HIS tagged SRMT.3 (6 × HIS-SRMT.3) in vitro, and the pulled-down content of SRMT.3 increased with the abundance of SRMT.3 during incubation, whereas the GST protein as the control could not pull-down the SRMT.3 at all. **C** The Co-IP assay displayed that the FLAG tagged SRMT.3 (SRMT.3-FLAG) could interact with GFP-tagged PtoNF-YC9.1 (PtoNF-YC9.1-GFP) in vivo. **D** The transient transformation of poplar mesophyll protoplasts with GFP-tagged PtoNF-YC9.1 indicates that the GFP-PtoNF-YC9.1 localized both in the cytoplasm and nucleus without ABA treatment, while this recombination protein was more abundant in the nucleus than in cytoplasm after ABA treatment. However, the subcellular localization of free GFP was not changed after ABA treatment. **E** Western blotting indicates that the FLAG tagged PtoNF-YC9.1 (PtoNF-YC9.1-FLAG) to be more abundant in the nucleus after ABA treatment than without ABA treatment. **F** The abundance of PtoNF-YC9.1-FLAG in nucleus and cytoplasm with or without salt stress

The transcription of *PtoRD26* in response to salt and ABA requires *SRMT*, but salt- and ABA-inducible *SRMT* lags behind *PtoRD26*, suggesting that this process may rely on regulation at the protein level. To verify this assumption, a yeast two-hybrid (Y2H) screening was performed with an SRMT.3 truncated C-terminal of 72 residues (SRMTΔ^261-332^) as the bait (Additional file 1: Fig. S19). We found that a NF-YC (*P.x_tomentosa23609*) had the most hits (Additional file 12: Table S11) and shares a high sequence similarity in the HAD domain of homologs (like AtNF-YC9) from various species (Additional file 1: Fig. S20A); we therefore named it here as PtoNF-YC9.1. We then verified the physical interaction between PtoNF-YC9.1 and SRMT.3 in vitro and in vivo through a point-by-point Y2H assay, a pull-down assay, and a co-immunoprecipitation (Co-IP) assay (Fig. 6A-C). Similar to *SRMT*, *PtoNF-YC9* is predominantly expressed in mature leaves and petioles (Additional file 1: Fig. S20B); moreover, ABA and salt stress have no significant influence upon the expression of *PtoNF-YC9* (Additional file 1: Fig. S20C-E), suggesting that PtoNF-YC9 functions at the protein level rather than the transcription level. We therefore speculate that SRMT regulation of *PtoRD26* expression in response to ABA and salt may rely on the PtoNF-YC9 proteins.

The NF-YC protein was found to be transferred into the nucleus when stress occurs and where plants can integrate endogenous and environmental signals effectively [45]. To determine whether PtoNF-YC9 acts like this in an ABA-mediated salt response in this triploid poplar, we observed the subcellular localization of the GFP-fused PtoNF-YC9.1 (GFP-PtoNF-YC9.1) in the mesophyll cell protoplasts, with or without ABA treatment. We found GFP-tagged PtoNF-YC9.1 to be localized both in cytoplasm and nucleus without ABA treatment; however, some protoplasts exhibited strong GFP signals in the nucleus rather than cytoplasm under ABA treatment (Fig. 6D). In addition, we overexpressed the PtoNF-YC9.1 which was tagged by FLAG (PtoNF-YC9.1-FLAG) and examined the protein abundance in both the cytoplasm and nucleus. In comparison to the samples without treatments, more PtoNF-YC9.1-FLAG accumulated in the nucleus than in the cytoplasm after ABA or salt treatment (Fig. 6E-F). Together with the localization of SRMT only in the nucleus, we propose that ABA and salt could drive PtoNF-YC9 to be transferred into the nucleus and then to interact with SRMT.

### PtoNF-YC9 enhances the effect of SRMT on the positive regulation of PtoRD26 expression and so confers greater salt tolerance in the triploid poplar

**Fig. 7:**
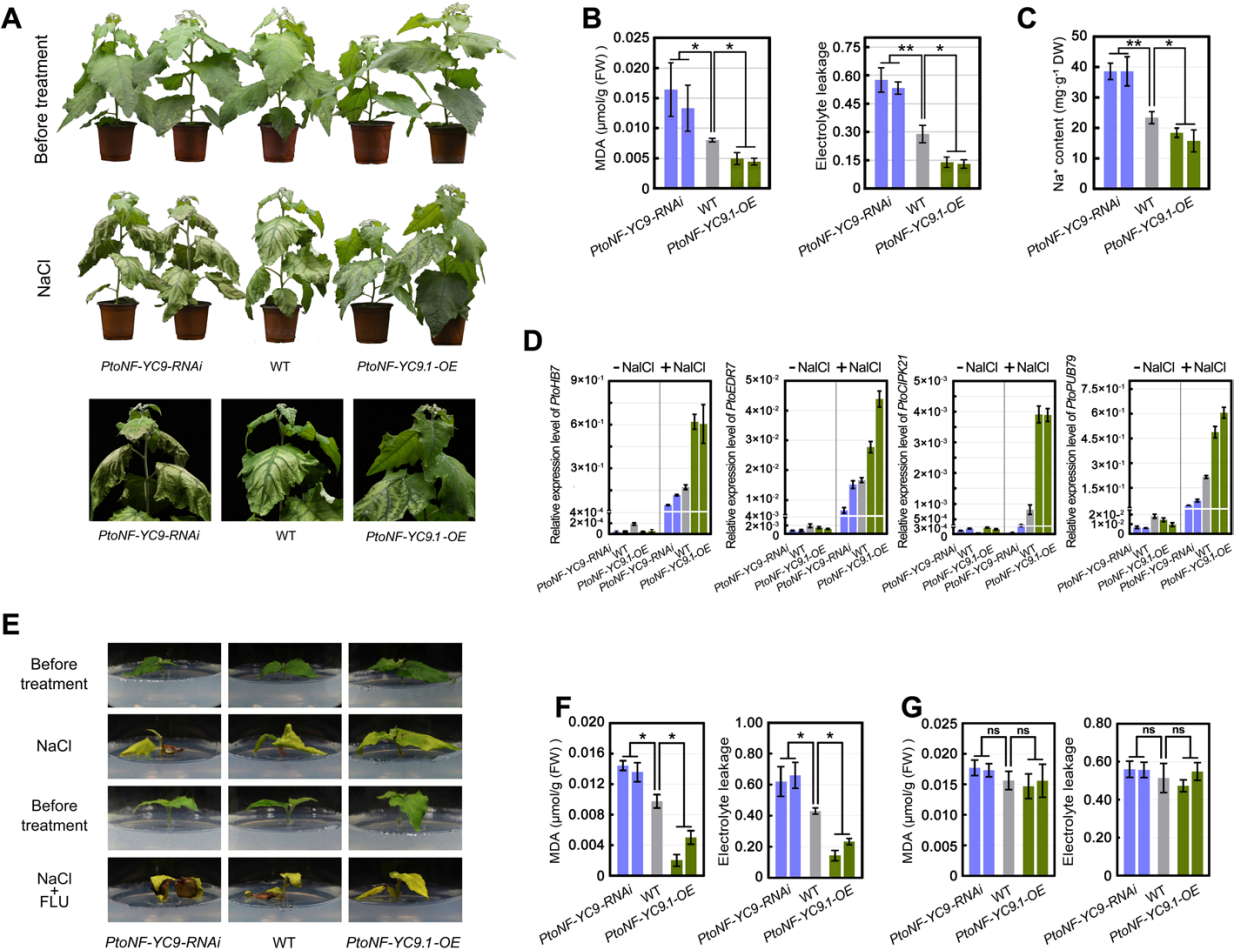
*PtoNF-YC9* positively regulated poplar tolerance to salt stress is dependent on the ABA signaling pathway. **A** The salt tolerance of 45-day-old *PtoNF-YC9-RNAi*, WT, and *PtoNF-YC9.1-OE* poplars in soil treated with 300 mM NaCl solution. **B** The MDA content and EL in the poplar leaves (**A**) after salt treatment. **C** The Na^+^ content of the leaves of poplars (**A**) after salt treatment. **D** The expression levels of *PtoHB7*, *PtoERD7*, *PtoCIPK21*, and *PtoPUB79* in the *PtoNF-YC9* overexpression, RNAi, and WT poplars with or without salt stress by qPCR, respectively. Letters above bars represent statistically significant differences between groups (*P* < 0.05) as determined by one-way ANOVA Duncan’s test. **E** The salt tolerance of *PtoNF-YC9-RNAi*, WT, and *PtoNF-YC9.1-OE* in the WPM solid medium supplemented with 150 mM NaCl or 150 mM NaCl plus 10 μM FLU. **F** The MDA content and EL of the poplar leaves in WPM solid medium with 150 mM NaCl. **G** The MDA content and EL of the poplar leaves in WPM medium with NaCl and FLU. The means ± s.d. of all data from at least three biological replicates are shown. Asterisks indicate statistically significant differences (two-sided Student’s *t* test, **P < 0.01, *P < 0.05, ns = not significant)

To determine the function of *PtoNF-YC9* in response to salt stress, we generated the overexpressing (*PtoNF-YC9.1-OE*) and the RNAi knocking-down (*PtoNF-YC9-RNAi*) poplars (Additional file 1: Fig. S21). Compared to the WT poplar, the *PtoNF-YC9.1-OE* and *PtoNF-YC9-RNAi* poplars exhibited no significant differences in plant height, but increased and reduced tolerance to salt stress, respectively (Fig. 7A). The measurement of Na^+^ content, MDA content, and EL ratio in these lines supported these observations (Fig. 7B, C). The expression levels of *PtoHB7*, *PtoEDR7*, *PtoCIPK21*, and *PtoPUB79* were enhanced in the *PtoNF-YC9.1-OE* poplars, but reduced in the *PtoNF-YC9-RNAi* ones after salt treatment (Fig. 7D). However, salt tolerance in the phenotype as conferred by overexpressing *PtoNF-YC9.1* could be removed in the presence of FLU (Fig. 7E–G). These results indicated that the *PtoNF-YC9-*positive regulation of salt tolerance depends on ABA.

**Fig. 8:**
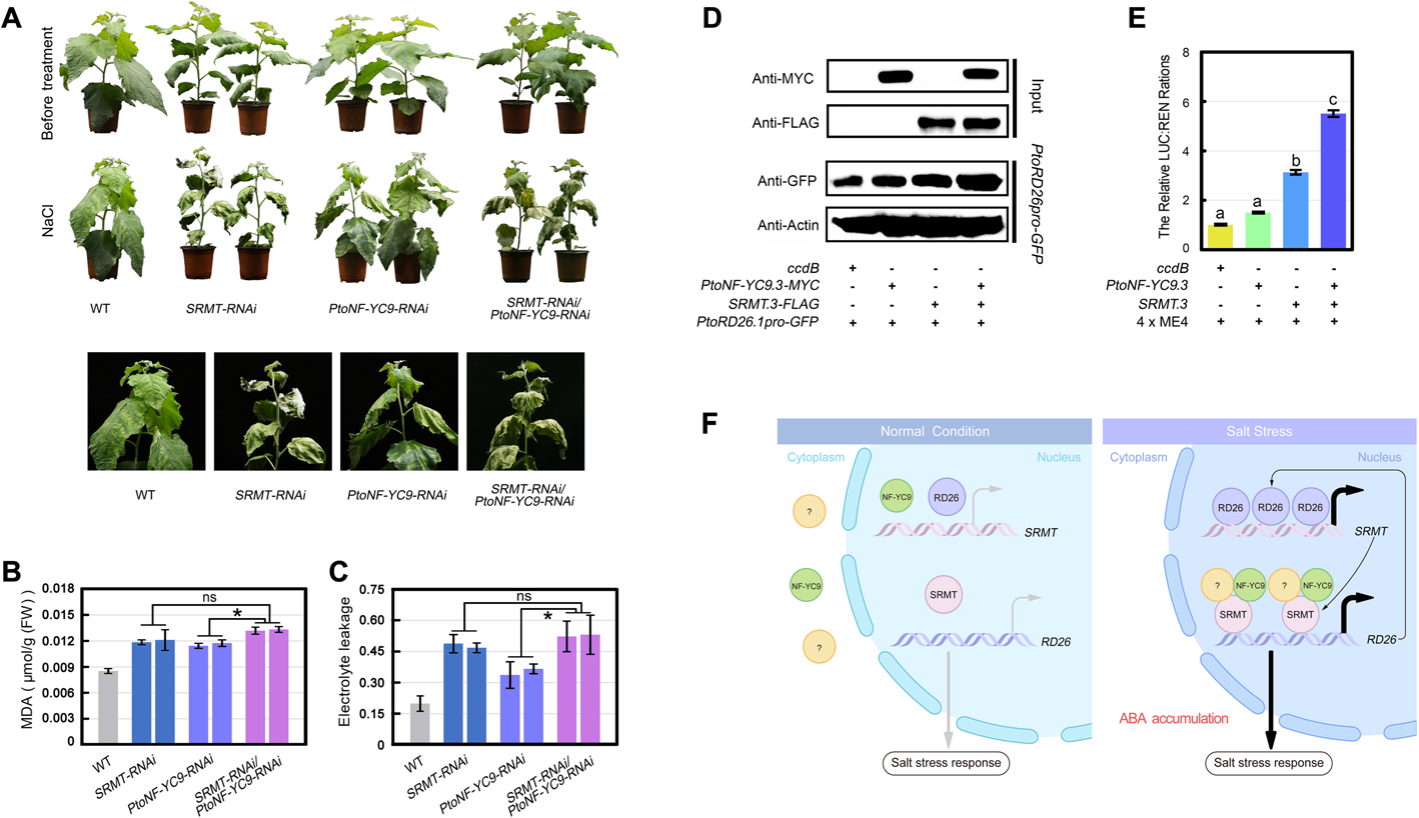
SRMT and PtoNF-YC9 co-regulates the expression of *PtoRD26* in response to salt stress. **A** The salt tolerance of *SMRT-RNAi/PtoNF-YC9-RNAi*, the double transgenic poplars (L18 and L31) in which the *SRMT* was knocked down in the background of *PtoNF-YC9-RNAi-*L17 compared to the *SRMT-RNAi*, *PtoNF-YC9-RNAi*, and WT poplars. **B** The MDA content of different lines after salt treatment. **C** The EL of different lines after salt treatment. The means ± s.d. of all data from at least three biological replicates are shown. Asterisks indicate statistically significant differences (two-sided Student’s *t* test, *P < 0.05, ns = not significant). **D** Western blotting indicates that the GFP abundance, driven by *PtoRD26* promoter, was enhanced by expressing PtoNF-YC9.1-MYC, SRMT.3-FLAG, or co-expressing these two fused proteins. **E** The dual-luciferase assay indicates that *LUC*, driven by the artificial promoter constructed by fourfold ME4 repeats fused with the 35S minimal promoter, was significantly enhanced by co-expressing *SRMT.3* and *PtoNF-YC9.1* rather than expressing these two genes separately. **F** The working model of *SRMT*, *PtoNF-YC9* and *PtoRD26* in the ABA-dependent salt-stress response. The question marks indicate the unknown proteins. The gray arrows indicate the low expression levels of *SRMT* and *PtoRD26*, and the back bold arrows indicate the significantly enhanced expression levels of *SRMT* and *PtoRD26*

To reveal the genetic relationship of *PtoNF-YC9* and *SRMT*, we knocked down the *SRMT* by RNAi under a *PtoNF-YC9-RNAi* background (*SRMT-RNAi/PtoNF-YC9-RNAi*) (Additional file 1: Fig. S22). The *SRMT-RNAi/PtoNF-YC9-RNAi* poplars exhibited a clear sensitivity to salt compared with both the WT and *PtoNF-YC9-RNAi* poplars (Fig. 8A–C). However, they were similar to the *SRMT-RNAi* lines (Fig. 8A–C), indicating that *SRMT* is epistatic to *PtoNF-YC9* and that both genes are on the same salt-responsive signaling pathway. To determine how the interaction between SRMT and PtoNF-YC9 influences *PtoRD26* expression, a *GFP* reporter gene driven by the promoter of *PtoRD26.1* was transiently transformed into the leaves of *Nicotiana benthamiana.* We found that co-expressing *PtoNF-YC9.1* and *SRMT.3* significantly increased the GFP abundance than when each was expressed alone (Fig. 8D). We also used an artificial promoter constructed by fusing ME4 with 35S minimal promoter to drive the *LUC* in a dual-LUC assay. The results indicated that SRMT.3 could significantly upregulate the LUC fluorescence intensity, whereas PtoNF-YC9.1 could not (Fig. 8E). However, co-expressing *SRMT.3* and *PtoNF-YC9.1* gave the most intense fluorescence (Fig. 8E). These results indicate that PtoNF-YC9 can enhance the transcriptional activity of SRMT to *PtoRD26*. It should be noted that *PtoNF-YC9.1* and *PtoNF-YC9.2* share identical CDS, while PtoNF-YC9.3 has four nonsynonymous variations (Additional file 1: Fig. S23). Moreover, the *PtoNF-YC9.3* with *SRMT* displayed similar transcriptional activate activity to target promoter compared to *PtoNF-YC9.1* (Additional file 1: Fig. S24).

## Discussion

ABA is recognized as having an important role for a plant’s ability to sense stress, especially in salt-susceptible *Populus* species [46]. Our previous studies suggested that a transcriptional inhibition of *RD26* mediated by the *WRKY77-PUB79* module provoked an ABA-dependent salt response in poplar [24, 27]. However, the molecular mechanism underlying any putative interaction between ABA and *RD26* has remained unclear. In our present study, we clarified this molecular mechanism and revealed that ABA drives PtoNF-YC9 to be shuttled into the nucleus where it interacts with and upregulates SRMT. The upregulation of *SRMT* in turn triggers the rapid expression of *PtoRD26*. This positive feedback loop of *SRMT*-*PtoRD26* can quickly amplify salt-stress signaling. We therefore proposed a working model for the PtoNF-YC9-SRMT-PtoRD26 module in signaling transduction of the salt response (Fig. 8F): Under normal conditions with low ABA content, PtoNF-YC9 is localized in the cytoplasm where, therefore, low abundance of SRMT cannot significantly activate the transcription of *PtoRD26*. When salt stress occurs, it triggers an accumulation of ABA, and a mass of PtoNF-YC9 proteins are translocated from the cytoplasm to the nucleus, where it interacts with SRMT to dramatically elevate the transcription level of *PtoRD26*. The increased PtoRD26 in turn positively regulates *SRMT*, which forms a positive feedback loop to amplify the signaling.

The NF-YC proteins are structurally characterized by a histone-fold domain (HFD) and closely related to the core histone H2A. They functionally act as one subunit of the NF-YA-YB-YC complex (including NF-YA, NF-YB, and NF-YC) that specifically recognizes the CCAAT-box in eukaryotes, or the NF-YB-YC-TF (the NF-YA is replaced by another transcription factor (TF)), to regulate plant development and environmental adaptation [35, 36, 45, 47, 48]. NF-YC proteins localized both in cytoplasm and nucleus comprise an essential prerequisite for the translocation of NF-YBs into the nucleus through a piggyback mechanism [48], suggesting that the NF-YB-YC heterodimer acts like a messenger to trigger transcriptional reprogramming. This may explain the regulatory scheme of the ABA response through hierarchical clustering analyses in *Arabidopsis*: i.e., that NF-YB2 and NF-YC2 are of fundamental importance in mediating ABA responses and function upstream of most responsive elements [15]. In our study, we found that ABA or salt treatment can drive PtoNF-YC9.1 to be translocated from the cytoplasm to the nucleus (Fig. 6D–F). However, the interacting NF-YBs for such a translocation remain unknown. In poplar, 21 *NF-YB* genes were identified, and *NF-YB1*, *NF-YB10*, and *NF-YB12* are known to be significantly induced by ABA, PEG, and drought [49]. Further evidence is needed to confirm whether these NF-YBs dimerized with PtoNF-YC9 in response to ABA signaling. Unless post-translational modifications mediate the interaction between NF-YB and PtoNF-YC9 to translocate the latter from the cytoplasm to nucleus, some other regulators must interact with PtoNF-YC9 to trigger its localization in the nucleus. Otherwise, the de novo transcription of stress-induced *NF-YB* may not explain well the rapid cytoplasm-nucleus shuttling of PtoNF-YC9.1 when stress occurs (Fig. 6D–F). The expression level of *PtoNF-YC9* was not obviously affected by ABA treatment and salt stress (Additional file 1: Fig. S20C-E), suggesting that its transcription may not be ruled by the canonical PYR/PYL/RCAR-PP2Cs-SnRK2s-ABFs module of the ABA signaling pathway. In the Y2H screening, we found a heavy metal-associated isoprenylated plant protein 26 (HIPP26) to interact with SRMT (Additional file 12: Table S11). HIPP26 predominantly occurs in mature leaves [50], which is similar to *PtoNF-YC9* (Additional file 1: Fig. S20B), and its subcellular localization at the plasma membrane and plasmodesmata is mediated by lipidation (*S*-acylation and prenylation), while the nucleus-localized, nonlipidated NbHIPP26 is predominantly responsible for the response to drought [50]. Whether there is an elusive plasma membrane-cytoplasm-nucleus ABA signal transduction mediated by HIPP26-PtoNF-YC9-SRMT-PtoRD26 in the triploid poplar demands further elucidation of the relationships between HIPP26 and PtoNF-YC9, as well as SRMT.

Feedback loops in plants can be an economical and efficient way to amplify a critical response or to avoid a detrimental over-activation of signaling, especially of defense responses [27, 51–54]. In poplar, the role of the *WRKY77*-*PUB79* feedback loop seems to be limited to the rapid amplification of the ABA-dependent stress signals [27], but this can be enhanced by the *SRMT*-*PtoRD26* positive feedback loop. These two mechanisms ensure a fast and sufficient signal intensity to orchestrate the necessary interactions among the comprehensive set of stress-associated genes and metabolites. Feedback loops also enable the complex integration of developmental, environmental, and other defense-associated signals [51]. Knocking-down *SRMT* in poplar, where it is predominantly expressed in mature leaves (Additional file 1: Fig. S8A-B), caused a crimped phenotype of the mature and old leaves (Additional file 1: Fig. S12D). This suggests that *SRMT* has indispensable functions in the cross-talk between leaf development and salt response and requires further investigation.

In conclusion, we have revealed a novel ABA-dependent salt-responsive mechanism, which is mediated by the PtoNF-YC9-SRMT-PtoRD26 module and confers salt tolerance to a triploid poplar. Although based on well-assembled genomes, we found some allelic variations of three genes among three sets of sequences, the critical regulatory elements are conserved in this triploid poplar. The haplotype-resolved genome assembly presented here enables this triploid to be used as appropriate transgenic material for further studies of other molecular mechanisms and to examine how allelic variations may affect such mechanisms. In addition, such genome sequences are useful for genetically modifying this triploid to create more poplar cultivars with widespread adaptability. In fact, our overexpressing *PtoNF-YC9.1*, *SRMT.3*, and *PtoRD26.1* genes, while increasing the salt tolerances of the resulting poplars, did not exhibit any obvious detrimental influence on other aspects of poplar growth (including plant height and stem diameter) (Figs. 2A, 4A, 7A and Additional file 1: Fig. S25). These genes may therefore also serve as potential and important modification targets in breeding programs.

## Conclusions

A haplotype-resolved genome sequence of *P. × tomentosa* Carr. clone 741 has been generated and its three haplotypes have been distinguished according to the descending order of similarity to the genome of *P. alba* var. *pyramidalis*. Based on this high quality of genome information, we have revealed a novel ABA-dependent salt-responsive mechanism, which is mediated by the PtoNF-YC9-SRMT-PtoRD26 module that confers salt tolerance to this triploid poplar. These findings widen and deepen our understanding how perennial woody plants respond to salt stress.

## Methods

### Plant materials and treatments

The triploid Chinese white poplar cuttings were propagated by tissue culture using woody plant medium (WPM) supplemented with 0.1 mg/L naphthylacetic acid (NAA) at 25 °C with a 14 h / 10 h light / dark photoperiod (4500 lux). The rooted plantlets were transplanted into soil in the greenhouse at 25 °C and 16 h / 8 h light / dark cycle. For NaCl treatment, the 3-week-old plantlets were transplanted into soil for 45 days, cultured in the greenhouse, and then watered with 300 mM NaCl solution for 5 to 7 days. Meanwhile, the 3-week-old sterile cuttings were transferred to WPM medium with either 150 mM NaCl (with or without 10 μM fluridone) for 20 days or with 5 μM ABA for 40 days. To investigate the tissue expression patterns of *SRMT*, *PtoNF-YC9*, and *PtoRD26* in poplar, the 2-month-old triploid Chinese white poplar plantlets were divided into various tissues, including first expansion leaf (young leaf, YL), 5th node leaf (mature leaf, ML), primary root (PR), lateral root (LR), stem (S), and petiole (P).

### RNA isolation and quantitative RT-PCR (qPCR)

Total RNA was extracted using TRIzol reagent (TaKaRa, Otsu, Japan). Quantity and quality were measured by a NanoDrop 2000 Spectrophotometer (Thermo Fisher Scientific, Waltham, MA). The reverse transcription of 2 μg total RNA was performed using a PrimeScript^TM^ RT reagent kit with gDNA Eraser according to the manufacturer’s protocol (TaKaRa, Shiga, Japan). The qRT-PCR analysis was performed using Real-Time EasyTM-SYBR Green I (FORE GENE Bio Inc., Chengdu, China) with a CFX384 Touch™ Real-Time PCR Detection System (BIO-RAD, USA). The qRT-PCR program parameters were 95 °C for 5 min, followed by 95 °C for 5 s and 60 °C for 30 s within 39 cycles. The relative expression levels were calculated using the (Et)_△CTt_/(Er)_△CTr_ ratio through normalizing ubiquitin gene (*UBQ*) of poplar [55]. All the specific primers used in qRT-PCR can be found in Additional file 13: Table S12.

### Genome assembly and RNA-seq

To estimate the genome size, we collected fresh tender leaves from the sequenced individual and analyzed them by flow cytometry (FCM). The sample was washed and chopped in ice-cold mG lysis buffer (45 mmol/L MgCl_2_, 30 mmol/L sodium citrate, 20 mmol/L MOPS,1% (w/v) PVP-40, 10 mmol/L EDTA·Na_2_, 0.2% (w/v) TritonX-100, 20 μL/mL β-mercaptoethanol) and finally dipped in 0.4 mg/mL propidium iodide (PI) for 10 min under dark conditions. On the basis of FCM, the genome size was 1.58 Gb, with *P. alba* var. *pyramidalis* [56] used as a standard. For genome assembly of the triploid Chinese white poplar, PacBio HiFi reads were assembled de novo using the Hifiasm assembler (v0.15.3-r339) [57] with default parameters where Hi-C pair-end reads were applied to generate haplotype-resolved contigs. To evaluate completeness of the assembly result, BUSCO2 (v3.0.2b) analyses were performed under the genome mode using the embryophyta_odb10 database [58]. RNA-seq pair-end reads and PacBio HiFi reads were mapped onto genome contigs using Hisat2 (v2.1.0; --dta) [59] and Minimap2 [60] (v2.21-r1071; --secondary=no -ax map-hifi) and the respective mapping rates were calculated.

Our RNA-seq data were generated from the Novo gene Bioinformatics Technology Co. Ltd. (Beijing, China) with the Illumina HiSeq 2000 system (Illumina, San Diego, CA). The clean reads were mapped to the triploid Chinese white poplar reference genome by the Hisat2 (v2.1.0) and only the best alignment for each read was retained according to the methods mentioned in the published tea plant haplotype genome [61]. Stringtie (v2.1.4) was used to measure gene expression levels, and to generate the fragments per kilobase of exon model per million mapped fragments (FPKM) [62]. We used DEseq2 [63] to identify differentially expressed genes (DEGs). Venny2.1 (https://bioinfogp.cnb.csic.es/tools/venny/index.html) was used to create the Venn diagram; the Volcano plot and heatmap of the DEGs were generated by EHBIO gene technology resources (http://www.ehbio.com/Cloud_Platform/front).

### Annotation of repetitive contigs

Repetitive sequences of the triploid Chinese white poplar genome were identified by both reference-based and de novo approaches. RepeatMasker (v4-0-7), Dfam_Consensus (v20170127), and Repbase5 (v20170127) databases of published repetitive elements were used to search for transposable elements and generate repetitive contigs [64], which were subsequently applied for de novo prediction using RepeatModeler (v1.0.11) with default parameters set to generate a repetitive contigs library. Results identified by both methods were merged into the final repeat dataset.

### Protein-coding gene prediction and functional annotation

The protein-coding genes of the triploid Chinese white poplar genome were identified by three methods: transcriptome-based, homology-based, and ab initio prediction. Firstly, protein sequences from six published *Populus* genomes, including *P. alba* var*. pyramidalis* [65], *P. trichocarpa* [66], *P. alba L.* [67], *P. tremula L*. [68], *P. tremuloides* [68], and the diploid *P. tomentosa* [69], were combined as homologous evidence for protein-coding genes predicted using the GeneWise6 (v2.4.1; --coverage_ratio 0.4 --evalue 1e-9) [70]. Secondly, genes were subsequently predicted by Transdecoder (https://github.com/TransDecoder; LongOrfs: -m 100 -G universal; Predict: --retain_long_orfs_mode dynamic) in transcriptome-based methods. In addition, ab initio prediction was performed by Augustus [71] (v3.2.3; --gff3=on --allow_hinted_splicesites=gcag,atac --alternatives-from-evidence=true --min_intron_len=30 --softmasking=1) where the training model was obtained based on the transcriptome and homology-prediction results using scripts of the GETA (https://github.com/daizao/geta) software. Finally, all the three results were combined using GETA to generate high-quality gene models which were further validated by the Pfam9 (v3.0) database [72].

The protein sequences translated from the final gene dataset were evaluated using BUSCO [58] under protein mode with the embryophyta_odb10 database. The Gene functions were annotated using BLASTP (v2.7.1+; 1e−5) against the Swiss-Prot (v20190725) database, Clusters of orthologous groups for eukaryotic complete genomes (COG, v2.2.26) database, and Non-Redundant Protein Sequence Database (NR, v20190123). Gene Ontology (GO) and Kyoto Encyclopedia of Genes and Genomes (KEGG) pathways were annotated using InterproScan10 (v5.44-79.0) [73] and KOBAS11 (v3.0) [74], respectively.

To annotate non-coding RNAs, tRNA genes were predicted using tRNAscan-SE (v2.0) [75], rRNA fragments were identified by RNAmmer (v1.2; -S euk -m lsu,ssu,tsu) [76] and microRNA/snRNA genes were detected using INFERNAL [77] (v1.1.2; -Z 4393.667106 --cut_ga --rfam --nohmmonly --fmt 2) against the Rfam (v14.0) database [78].

### Co-expression analysis

The total gene set was filtered according to the expression level in each sample to improve the accuracy of network construction, and 44,939 genes were selected. A weighted gene co-expression network was subsequently constructed using the R packages WGCNA (v.147) [79] with the soft power of 12. The module eigengene expression, adjacency matrix heatmap, Module-Trait relationships, and co-expression gene modules were calculated based on Pearson correlation. Finally, the module “lightpink1” consisting of *RD26* and *SRMT* was selected and their co-expressed genes corresponding to salt tolerance were visualized by using the website (http://www.ehbio.com/test/venn/#/) [80].

### Vector construction

To construct the vectors, specific primers were used to clone *SRMT*, *PtoNF-YC9*, and *PtoRD26* from the poplar cDNA library (Additional file 13: Table S12) by PCR with Phanta^TM^ Super-Fidelity DNA Polymerase (Vazyme, China). The PCR products were then ligated into the pCXSN vector or pBIB-BASTA-35S-GWR-GFP/FLAG to drive genes by the cauliflower mosaic virus 35S promoter (35S). The RNAi vectors were constructed based on the pCSXN skeleton [81]. The promoter fragments of *SRMT* were amplified from the genomic DNA of triploid Chinese white poplar and cloned into the vector pCXGUS-P [81].

### Plant transformation

The transformation of triploid Chinese white poplar was performed using *Agrobacterium*-mediated methods with the media components as previously described [33, 82]. In the process of transformation, discs were cut from healthy leaves at the second or third internode from the lower terminal bud. The leaf discs were immersed in *Agrobacterium* suspension and incubated on a constant temperature shaker at 28 °C for 10 min; any excess solution was removed with sterile filter paper; the leaf discs were cultured for 48 h. The discs were then transferred to the selective medium for inducing callus. The callus was transferred to the shoot medium, which was changed for fresh medium every 2 weeks until the height of the shoot was about 2 cm when they were cut and placed into rooting medium.

### GUS staining

As described previously [83], various tissues of transgenic poplar expressing the β-glucuronidase (GUS) reporter gene driven by the promoter of *SRMT* were selected for histochemical staining. In order to verify whether the *SRMT* promoter responds to ABA, mannitol, and salt treatments, the leaves were detached and immersed in MS liquid medium with or without 10 μM ABA, 150 mM NaCl, or 200 mM mannitol for 6 h, respectively as described previously [84]. GUS staining was then performed using β-Glucuronidase Reporter Gene Staining Kit (Scientific Phygene, Chendu, China) according to the manufacturer’s instructions. In order to verify the transcriptional activity of the *SRMT* promoter in different tissues of poplar, well-grown transgenic poplar from the greenhouse was divided into various tissues, including first expansion leaf (young leaf, YL), 5th node leaf (mature leaf, ML), root (R), stem (S), and petiole (P), to performed the GUS staining.

### *Yeast library screening*, *two-hybrid (Y2H) and one-hybrid (Y1H) assays*

For the Y2H screening, we constructed pGBKT7 (BD) vectors containing the SRMT.3 gene fragments, SRMT.3-△N^1-22^, SRMT.3-R2R3, SRMT.3-△C^261-332^, and SRMT.3-△C^229-332^ by enzyme digestion (EcoRI) and ligating (T4 DNA ligase). These respective plasmids were then introduced into the AH109 yeast strain for self-activating identification. Finally, we selected BD-SRMT.3-△C^261-332^ as the bait to perform the yeast library screening according to the Matchmaker^TM^ GAL4 Two-Hybrid System 3 & Libraries User Manual.

For the Y2H assay, the CDS of *PtoNF-YC9.1* was cloned and inserted into the pGADT7 (AD) vector to generate the AD-PtoNF-YC9.1 plasmid. We transformed BD-SRMT.3-△C^261-332^, combined AD or AD-PtoNF-YC9.1 plasmids into the AH109 yeast strain, and screened the transformants on synthetic dropout (SD) medium without tryptophan (W) and leucine (L) at 30 °C for 2 days. The positive transformants were cultured in SD liquid medium without W and L in a shaker at 30 °C for 2 days. The yeast suspension was then adjusted to OD_560_ = 1.0, 0.1, 0.01, and 0.001 using TE buffer. The diluted suspension was dropped on SD solid medium without W, L, adenine (A) or histidine (H) (SD-W/L/A/H), and supplemented with 50 mM 3-amino-1,2,4-triazole (3-AT) to identify the interaction.

For the Y1H assay, the RE2 motif and its mutant formed respective fragments with their flanking sequences in the *SRMT.3* promoter. These two type-fragments were replicated three times and fused to the pAbAi vector, while CDS of *PtoRD26.1* gene was cloned to the pGADT7 vector to create the AD-PtoRD26.1 vector. The AD and AD-PtoRD26.1 vectors with the RE2 motif or its mutant were then transformed into the Y1H gold yeast strain by the PEG/LiAc method [85]. Finally, all the transformants were screened on SD solid medium without L and uracil (U) (SD-L/U) and supplemented with aureobasidin A (AbA) to identify the interaction.

### Pull-down assay

The CDS of *SRMT.3* and *PtoNF-YC9.1* were ligated to the pET-30a and pGEX4T-1 vectors, respectively. The recombinant 6×His-SRMT.3 and GST-PtoNF-YC9.1 proteins were purified using Ni-NTA agarose (Qiagen) and Glutathione-Sepharose beads (GE), respectively. The GST-PtoNF-YC9.1 or GST protein were immobilized with Glutathione-Sepharose beads and incubated with 6×His-SRMT.3 in the incubation buffer (50 mM Tris-Cl, 100 mM NaCl, 0.25% TritonX 100, 35 mM β-mercaptoethanol; pH 7.8) in a shaker with 80 rpm at 4 °C for 3 h. Unbound proteins were then removed by rinsing six times in the incubation buffer with 5 × SDS loading buffer added into the mixture, which was then heated to 100 °C for 10 min. After centrifugation at 12,000 rpm for 1 min, the supernatant was separated by 12% SDS-PAGE and visualized by immunoblot analysis with anti-GST or anti-His antibodies (Merck Millipore, Billerica, USA).

### Immunoprecipitation assay

For Co-IP assay, we expressed SRMT.3-FLAG or PtoNF-YC9.1-GFP-fused proteins alone or together in the leaves of 3-week-old *N. benthamiana* mediated by *Agrobacterium*. The leaves were harvested and thoroughly ground in liquid nitrogen. As previously reported [86], the nuclear extraction buffer (20 mM HEPES, pH 7.5, 1%, TritonX-100, 1 mM EDTA, 40 mM KCl, 10 mM MgCl_2_, 5 mM DTT, 10% glycerin, 1 × cocktail, 4 mM PMSF, 40 μM MG132) was used to extract plant nuclear proteins. The crude protein extractions were centrifuged at 12,000 rpm, at 4 °C for 10 min, then re-suspended with 80 μl nuclear lysis buffer (10 mM EDTA, 5 mM DTT, 0.1% SDS, 50 mM Tris-HCl, pH 8.0) and placed on ice for 15 min, and finally, 600 μl dilution buffer was added (16.7 mM NaCl, 1.2 mM EDTA, 0.01% SDS, 16.7 mM Tris-HCl, pH 8.0). The ultrasound instrument was used to release nucleoprotein with a cycle of 5 s for sonication, 20 s for rest, repeated 20 times, at 4 °C. The nucleoprotein extraction was centrifuged at 12,000 rpm for 5 min and 600 μl of supernatant was placed in new 1.5-ml centrifuge tubes, 20 μl Anti-FLAG beads (Abcam) added and incubated for 2 h on a shaker with 80 rpm at 4 °C. After the reaction, the samples were centrifuged at 500 rpm and 4 °C for 1 min. The supernatant was discarded and the beads washed six times with 5× SDS loading buffer added and heated to 100 °C for 10 min. The washed samples were separated by 12% SDS-PAGE and visualized by immunoblot with anti-FLAG or anti-GFP antibodies (Abcam).

### Dual-luciferase assay

The ME2, ME4, and ME5 binding sites of SRMT in the promoter of *PtoRD26.1*, and their mutated forms were each repeated four times and fused with 35S minimal promoter to drive the *LUC* expression as the reporters. The effectors included recombinant plasmids of pBIB-BASTA-35S-SRMT.3-FLAG, pBIB-BASTA-35S-PtoRD26.1-FLAG, pBIB-BASTA-35S-PtoNF-YC9.1-FLAG, and pBIB-BASTA-35S-FLAG empty vectors. The reporters and effectors were transiently transformed into the leaves of 3-week-old *N. benthamiana* mediated by *Agrobacterium*. The infected tobacco was grown in the greenhouse for 2 days in darkness and 1 day in light. The infected leaves were then harvested, thoroughly ground in liquid nitrogen, and the enzyme mixture prepared in a Dual-Luciferase Reporter Assay kit (Promega) according to the manufacturer’s instructions. *Firefly* (*LUC*) and *Renilla* (internal control, *REN*) luciferase signals were detected by a multi-mode reader (Synergy H1; BioTek, Winooski, VT).

### The activation of PtoRD26 expression by SRMT and PtoNF-YC9

The 1.5-kb promoter region of *PtoRD26.1* was cloned to drive GFP expression as the reporter. The effectors included recombinant plasmids expressing SRMT.3-FLAG- and PtoNF-YC9.1-MYC-fused proteins. The reporters and effectors were transiently introduced into the leaves of 3-week-old *N. benthamiana* mediated by *Agrobacterium*. The *N. benthamiana* was grown in the greenhouse for 2 days in darkness and 1 day in light, and the infected leaves were harvested to extracted total protein. The extracted proteins were separated by 12% SDS-PAGE, and the GFP tag visualized by immunoblot with anti-GFP antibody.

### Subcellular localization and detecting PtoNF-YC9 translocation

To explore the subcellular localization of SRMT.3 and PtoNF-YC9.1, the CDS of these two genes were ligated to the pBI221 vector to express GFP-fused protein. The leaves of 3-week-old poplars were cut into 1-mm filaments and digested by submersion in 10 ml enzyme solution (0.4 M mannitol, 20 mM KCl, 1.5% Cellulase R10, 0.4% Macerozyme R10, 20 mM MES, pH 5.7) for 3 h. The mesophyll protoplasts in the enzyme solution were filtered through a 60-mesh sieve, and an equal volume of W5 solution (154 mM NaCl, 125 mM CaCl_2_, 5 mM KCl, 20 mM MES, pH 5.7) was added before centrifuging at 100×*g* for 2 min. The protoplasts were obtained by precipitation and re-suspension with 3 ml W5 solution. After setting for 30 min on ice, the protoplasts were re-suspended with 3 ml MMG solution (0.4 M mannitol, 15 mM MgCl_2_, 4 mM MES, pH 5.7) for transformation. For each group of samples, 100-μl protoplasts were taken and 15-μg recombinant plasmids (plasmid concentration ranged from 1 μg/μl to 2 μg/μl) were added for transformation by PEG method [87]. The pBI221 empty vector was used as a negative control. The GFP fluorescence was detected by laser-scanning under a confocal microscope (Leica TCS SP5) after being placed for about 16 h under dim light.

For PtoNF-YC9.1 translocation, protoplasts were transformed with 15 μg plasmids of pBI221-PtoNF-YC9.1 and pBI221. Every sample was prepared in two tubes: one had 5 μM ABA added; the other had W5 solution added as the control. After reacting for 1 h, a GFP fluorescence signal was detected. A further method to validate the translocation of PtoNF-YC9.1 in the presence of ABA was also performed. The leaves of a transgenic poplar expressing PtoNF-YC9.1-FLAG were sprayed with ABA solution (20 μM ABA, 0.01% TritonX-100, in PBS buffer, pH 5.8), while the control group was sprayed with pure water (plus 0.01% TritonX-100, in PBS buffer, pH 5.8). After 2 h, the leaves at the same internode were harvested and thoroughly ground in liquid nitrogen. The freeze-dried powder was suspended with the NEB buffer (20 mM HEPES, pH 7.5, 2%, TritonX-100, 1 mM EDTA, 40 mM KCl, 10% glycerin, 10 mM MgCl_2_, 5 mM DTT, 1× cocktail, 4 mM PMSF,40 μM MG132) and mixed by vortex. The mixture was left to settle at room temperature for 5 min. The mixture was then filtered through three layers of Miracloth (END Millipore, Billerica, USA), and the filtrate centrifuged at 15,000 rpm at 4 °C for 20 min. The supernatant can be used as a cytoplasmic protein extract. Meanwhile, 80 μl nuclear lysis buffer (10 mM EDTA, 5 mM DTT, 0.1% SDS, 50 mM Tris-HCl, pH 8.0) and 600 μl dilution buffer (16.7 mM NaCl, 1.2 mM EDTA, 0.01% SDS, 16.7 mM Tris-HCl, pH 8.0) was added to the precipitate which was then ultrasonicated and centrifuged at 12,000 rpm for 5 min. The supernatants containing nuclear protein and cytoplasmic protein then had 20 μl anti-FLAG beads (Abcam) added and were incubated in a shaker at 80 rpm and 4 °C for 2 h. The beads were then washed six times with 5× SDS loading buffer added and heated to 100 °C for 10 min. The proteins were separated by 12% SDS-PAGE and visualized by immunoblot with anti-FLAG antibody. The cytoplasmic and nuclear protein internal references were Rubisco and H3, respectively, the latter visualized by immunoblot with anti-H3 antibody.

### *The measurement of malondialdehyde (MDA) concentration*, *electrolyte leakage*, *and Na*^*+*^*content*

For MDA content [88], the leaves of poplar subjected to salt stress were ground to powder in liquid nitrogen and suspended in 5% trichloroacetic acid buffer. The suspension was centrifuged at 3000 rpm for 15 min, the supernatant was taken and an equal volume of TBA buffer added (6.7% thiobarbituric acid with 5% trichloroacetic acid). The mixture was incubated at 100 °C for 30 min, and then cooled to room temperature. The absorbance values of 100 μl mixture were measured at 532, 450, and 600 nm in a multi-mode reader (Synergy H1; BioTek, Winooski, VT).

For the electrolyte leakage (EL) [89], treated leaves were submerged in ultrapure water for 10 min; the EL values (EL1) were then measured with a DDS-307A Conductivity Meter (Leici, Shanghai, China). The samples were then incubated at 100 °C for 15 min and allowed to cool to room temperature before the EL values (EL2) were measured again. The ratio of EL1/EL2 indicates the degree of tissue damage.

To measure the Na^+^ content of poplars [90], leaves and stems of transgenic and WT poplars after salt stress were oven-dried at 65 °C for 3 days to complete dehydration. The dry material was then ground to powder in liquid nitrogen and incubated in 0.1 M acetic acid solution for 16 h. The insoluble fraction was removed by centrifugation at 12,000 rpm for 15 min. The Na^+^ content was measured by a 4300DV/5300DV ICP-OES instrument (Optima, Perkin-Elmer).

### Electrophoretic mobility shift assay (EMSA)

The CDS of *SRMT.3* and *PtoRD26.1* were ligated into the pGEX4T-1 plasmid to express GST-SRMT.3 and GST-PtoRD26.1 recombination proteins in *Escherichia coli* strain BL21 (DE3). The transformed BL21 was cultured in a shaker at 180 rpm and 37 °C until the OD_600_ value reached 0.6. The bacteria were then cultured at 16 °C for 14 h after adding 0.5 mM isopropyl-β-D-thiogalactoside (IPTG). The proteins were purified with Glutathione-Sepharose beads (GE). Biotin-labeled fragments were synthesized with Sangon probes (Shanghai, China) while un-labeled fragments were used as competitors. The EMSA was performed with a LightShift Chemiluminescent EMSA Kit (Pierce Biotechnology, IL, USA) according to the manufacturer’s instructions.

### Chromatin immunoprecipitation (ChIP)-qPCR

The DNA-protein mixture was extracted from mature leaves of 1-month-old transgenic poplars overexpressing *SRMT.3-FLAG* and WT poplar, using Protein A Agarose beads (Abcam, London, UK) to remove most miscellaneous proteins. Anti-FLAGantibody (Thermo Fisher Scientific, Waltham, USA) was then added and Protein A Agarose beads used to pull-down the DNA fragments bound by SRMT.3-FLAG recombination proteins. Finally, the proteins were removed and the DNA extracted by the CTAB method. qRT-PCR was performed to detect the immuno-precipitated DNA fragments as previously described [83]. The specific primers used in ChIP-qPCR are listed in Additional file 13: Table S12.

## Supplementary Information

Additional file 1. Figure S1. Flow cytometry estimation of the *P.* × *tomentosa* Carr. clone 741 genome size. Figure S2. The sequence identity of CDS (A), proteins (B) and 1.5 kb promoter sequences (C) among different haplotypes. Figure S3. Heatmap indicating the expression levels of differentially expressed genes (DEGs) from the RNA-seq of the salt-stressed triploid poplars. Figure S4. The tissue expression pattern of *PtoRD26* in this triploid poplar. Figure S5. Determination of *PtoRD26* expression level in the *PtoRD26.1* overexpression and RNAi lines. Figure S6. The sequence alignments of three *PtoRD26* alleles in the triploid poplars and their ortholog in *P. alba* var. *pyramidalis*. Figure S7. The phylogenetic relationship and peptide sequence of SRMT. Figure S8. Tissue expression pattern analysis of *SRMT* in the triploid Chinese white poplars. Figure S9. Both PtoRD26.2 and PtoRD26.3 upregulated the expression of *LUC* driven by *SRMT.3pro* promoter in tobacco leaves. Figure S10. The sequence alignments of two *SRMT* alleles in the triploid poplars and their ortholog in *P. alba* var. *pyramidalis*. Figure S11. The distributions of PtoRD26 binding elements in the promoters of *SRMT* alleles in this triploid poplar and in the promoter of the ortholog in *P. alba* var. *pyramidalis*. Figure S12. Identification of morphology of *SRMT* transgenic poplars. Figure S13. Sensitivity of *SRMT.3-OE*, *SRMT-RNAi* and WT poplar cuttings to ABA treatment. Figure S14. Both SRMT.2 and SRMT.3 upregulated the expression of *LUC* driven by *PtoRD26.1* promoter in tobacco leaves. Figure S15. EMSA indicated that the binding sites of SRMT.3 in the 1.5 kb promoter region of *PtoRD26.1*. Figure S16. The distributions of SRMT binding elements in the promoters of *PtoRD26* alleles in this triploid poplar and in the promoter of the ortholog in *P. alba* var. *pyramidalis*. Figure S17. The determination of the *PtoRD26* expression level in the double transgenic poplars (*PtoRD26-RNAi/SRMT.3-OE*). Figure S18. The temporal expression pattern of *PtoRD26* in response to MeJA was independent on *SRMT*. Figure S19. Self-activation activity identification of SRMT.3 in yeast strain AH109. Figure S20. The peptide sequence of NF-YC9 from various species and the spatio-temporal expression pattern of *PtoNF-YC9.1*. Figure S21. The expression levels of *PtoNF-YC9* in the *PtoNF-YC9.1* overexpression and RNAi poplars by qPCR. Figure S22. The determination of the *SRMT* expression level in the double knocking-down poplars (*SRMT-RNAi/PtoNF-YC9-RNAi*). Figure S23. The sequence alignments of three *PtoNF-YC9* alleles in the triploid poplars and their ortholog in *P. alba* var. *pyramidalis*. Figure S24. *SRMT* and *PtoNF-YC9* alleles were synergistically upregulated the expression of *LUC* driven by *PtoRD26.1* promoter in tobacco leaves. Figure S25. The plant height and stem diameter of all transgenic lines in this study.

Additional file 2. Table S1. The information of Hifi and Hi-C sequencing.

Additional file 3. Table S2. The differential expression genes with NaCl treatment compared to control.

Additional file 4. Table S3. The information of the RNA-seq data for co-expression analysis.

Additional file 5. Table S4. The genes co-expressed with *PtoRD26*.

Additional file 6. Table S5. The genes involved in salt, water stress and ABA in the co-expression analysis.

Additional file 7. Table S6. The genes used for co-expression network construct.

Additional file 8. Table S7. The differential expression genes in *SRMT* overexpression poplars compared to WT.

Additional file 9. Table S8. The positively regulated genes by both NaCl treatment and SRMT. The order of these genes was based on their expressions in *SRMT* overexpressing lines compared with WT.

Additional file 10. Table S9. The negatively regulated genes by both NaCl treatment and SRMT.

Additional file 11. Table S10. In the *SRMT* overexpressing lines, the genes with expression level in the top 700 were related to ABA, drought and salt stress, *etc*.

Additional file 12. Table S11. The potential interacting proteins of SRMT from Y2H screening assay.

Additional file 13. Table S12. The primers in this study.

Additional file 14. Uncropped images for the blots in Figs. 3D, 5C, 6B, 6C, 6E, 6F, 8D, and supplementary Figure 15. (PPTX 9666 kb)

Additional file 15. Review history.

## Data Availability

All sequencing data generated in this study have been submitted to the National Genomics Data Center (NGDC; https://bigd.big.ac.cn/bioproject) under BioProject accession number PRJCA009569 [91].
