# Supplementary material for: PtoNF-YC9-SRMT-PtoRD26 module regulates the high saline tolerance of a triploid poplar
Source: Genome Biol. 2022 Jul 7;23:148. doi: 10.1186/s13059-022-02718-7 (PMC9264554; doi:10.1186/s13059-022-02718-7)
Supplement: Supplementary file 1 — Additional file 1. Figure S1. Flow cytometry estimation of the P. × tomentosa Carr. clone 741 genome size. Figure S2. The sequence identity of CDS (A), proteins (B) and 1.5 kb promoter sequences (C) among different haplotypes. Figure S3. Heatmap indicating the expression levels of differentially expressed genes (DEGs) from the RNA-seq of the salt-stressed triploid poplars. Figure S4. The tissue expression pattern of PtoRD26 in this triploid poplar. Figure S5. Determination of PtoRD26 expression level in the PtoRD26.1 overexpression and RNAi lines. Figure S6. The sequence alignments of three PtoRD26 alleles in the triploid poplars and their ortholog in P. alba var. pyramidalis. Figure S7. The phylogenetic relationship and peptide sequence of SRMT. Figure S8. Tissue expression pattern analysis of SRMT in the triploid Chinese white poplars. Figure S9. Both PtoRD26.2 and PtoRD26.3 upregulated the expression of LUC driven by SRMT.3pro promoter in tobacco leaves. Figure S10. The sequence alignments of two SRMT alleles in the triploid poplars and their ortholog in P. alba var. pyramidalis. Figure S11. The distributions of PtoRD26 binding elements in the promoters of SRMT alleles in this triploid poplar and in the promoter of the ortholog in P. alba var. pyramidalis. Figure S12. Identification of morphology of SRMT transgenic poplars. Figure S13. Sensitivity of SRMT.3-OE, SRMT-RNAi and WT poplar cuttings to ABA treatment. Figure S14. Both SRMT.2 and SRMT.3 upregulated the expression of LUC driven by PtoRD26.1 promoter in tobacco leaves. Figure S15. EMSA indicated that the binding sites of SRMT.3 in the 1.5 kb promoter region of PtoRD26.1. Figure S16. The distributions of SRMT binding elements in the promoters of PtoRD26 alleles in this triploid poplar and in the promoter of the ortholog in P. alba var. pyramidalis. Figure S17. The determination of the PtoRD26 expression level in the double transgenic poplars (PtoRD26-RNAi/SRMT.3-OE). Figure S18. The temporal expression patt [file 13059_2022_2718_MOESM1_ESM.docx]

**Additional file 1:**


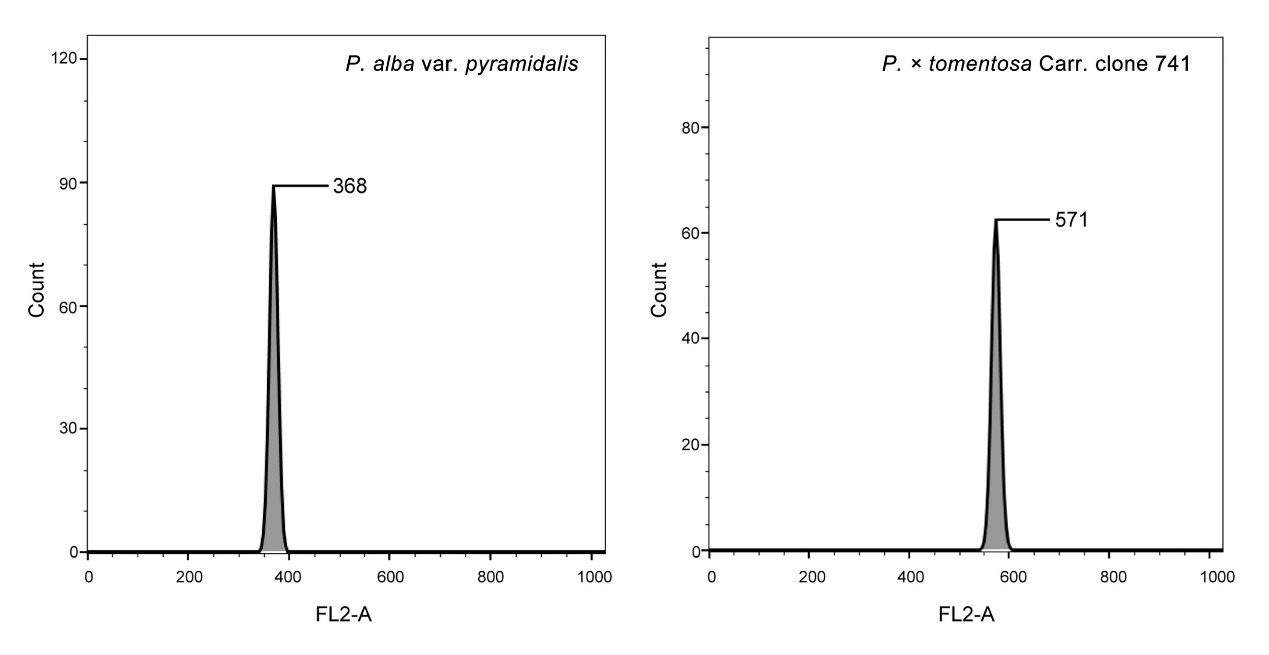


**Fig. S1.** **Flow cytometry estimation of the *P.* × *tomentosa* Carr. clone 741 genome size compared to reference standard of *P. alba* var. *pyramidalis* (508 Mb).** The *P. alba* var. *pyramidalis* (1.04 pg/2C) was used as internal reference standard [56]. Comparing with the fluorescence area signals (FL2-A) between the *P. alba* var. *pyramidalis* and the *P.* × *tomentosa* Carr. clone 741, the flow cytometry analysis provides an estimation of 1.61 pg DNA, which is equivalent to 1.58 Gb genome of *P.* × *tomentosa* Carr. clone 741 based on the formula: 1 pg = 0.978 Gb.


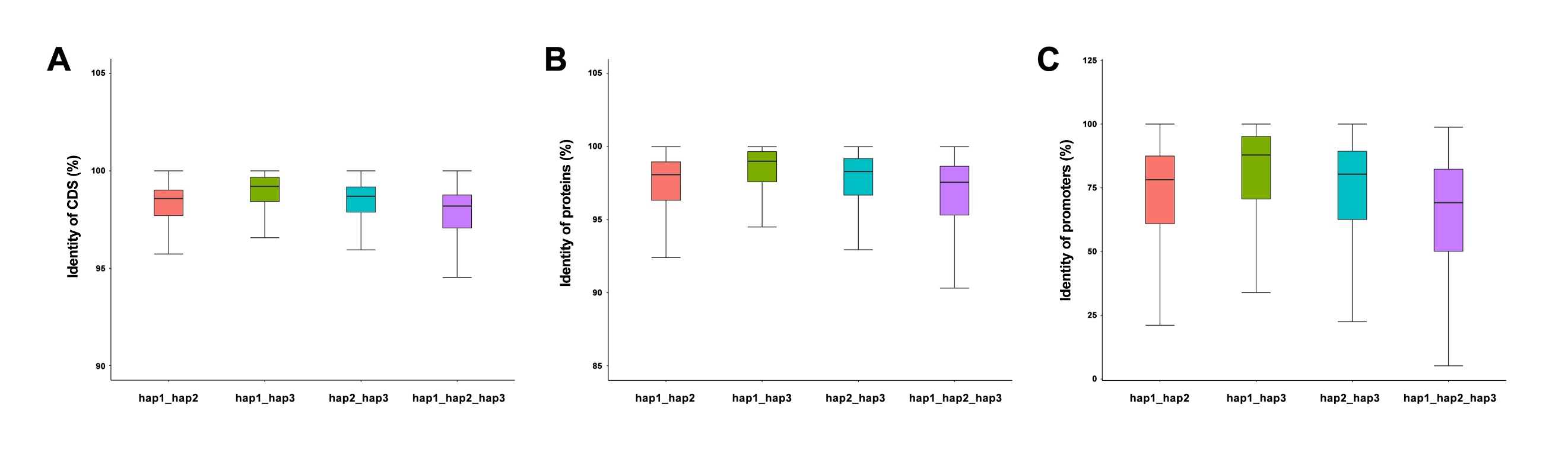


**Fig. S2.** **The sequence identity of CDS (A), proteins (B) and 1.5 kb promoter sequences (C) among different haplotypes.** For each allele group, sequences from the three haplotypes were mutually aligned by MUSCLE and sequence identity was subsequently calculated according to the alignment result. The central line for each box plot indicates the median value. The top and bottom edges of each box indicate the first and third quartiles and two whiskers extend 1.5 times of the quartile range beyond the box edges.


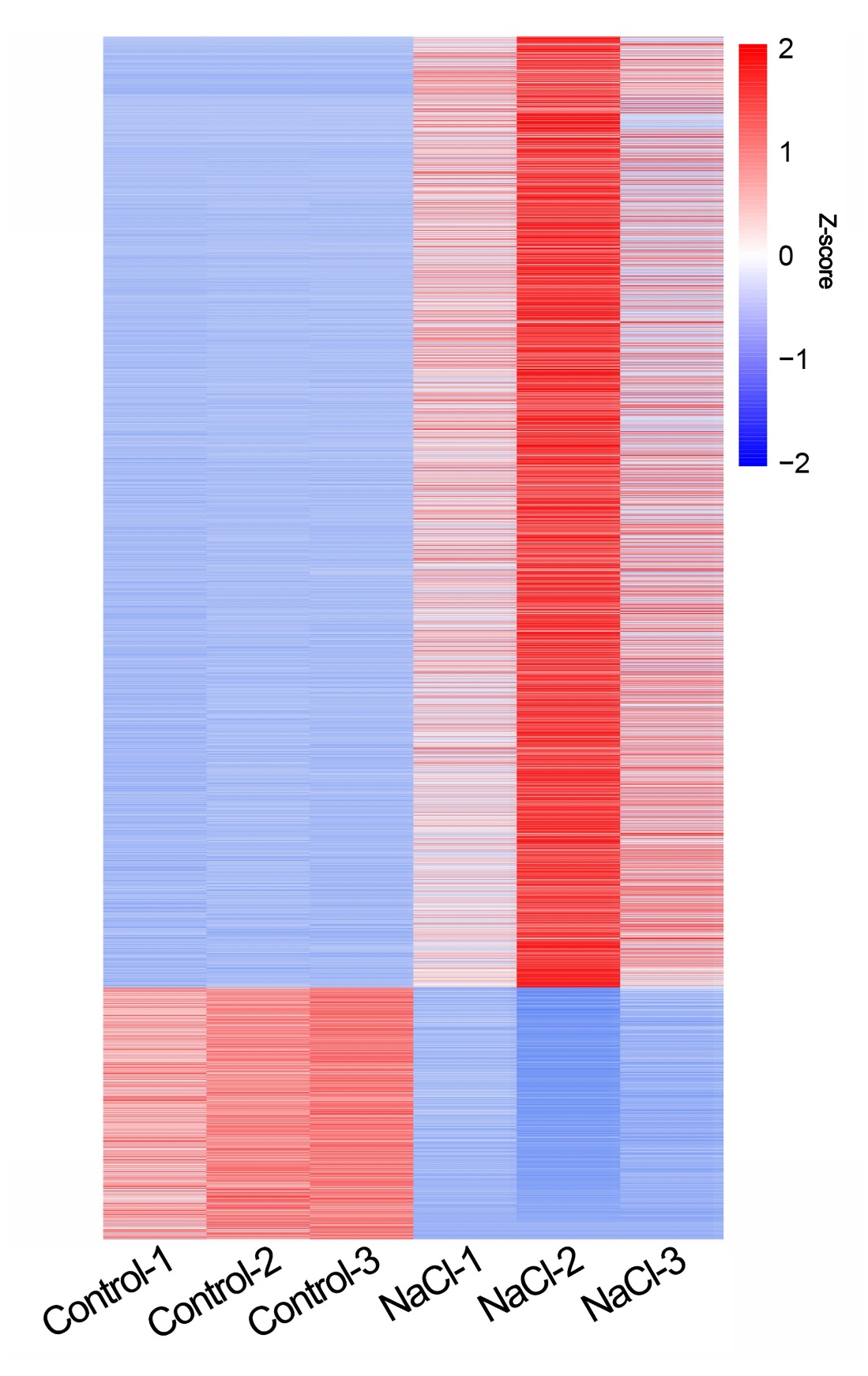


**Fig. S3. Heatmap indicating the expression levels of differentially expressed genes (DEGs) from the RNA-seq of the triploid Chinese white poplars treated with 300 mM NaCl solution, and of the controls with water.**


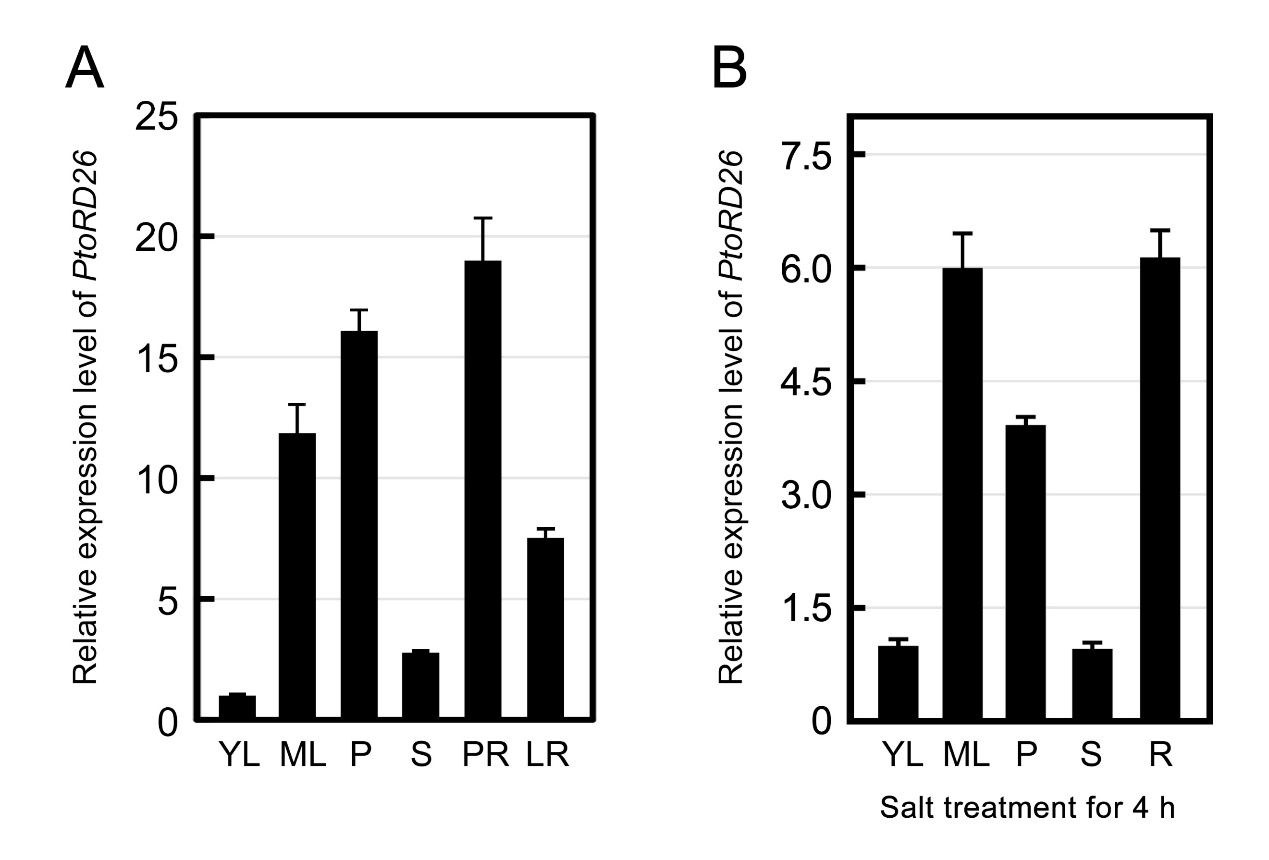


**Fig. S4. The tissue expression pattern of *PtoRD26* in the triploid Chinese white poplar under normal condition (A) and after salt treatment for 4 hours (B), respectively.** The qPCR revealed the transcriptional abundance of *PtoRD26* in various tissues, including first expansion leaf (young leaf, YL), 5^th^ node leaf (mature leaf, ML), petiole (P), stem (S), primary root (PR), lateral root (LR) and the total root (R). Of three independent experiments we present representative results from one experiment.


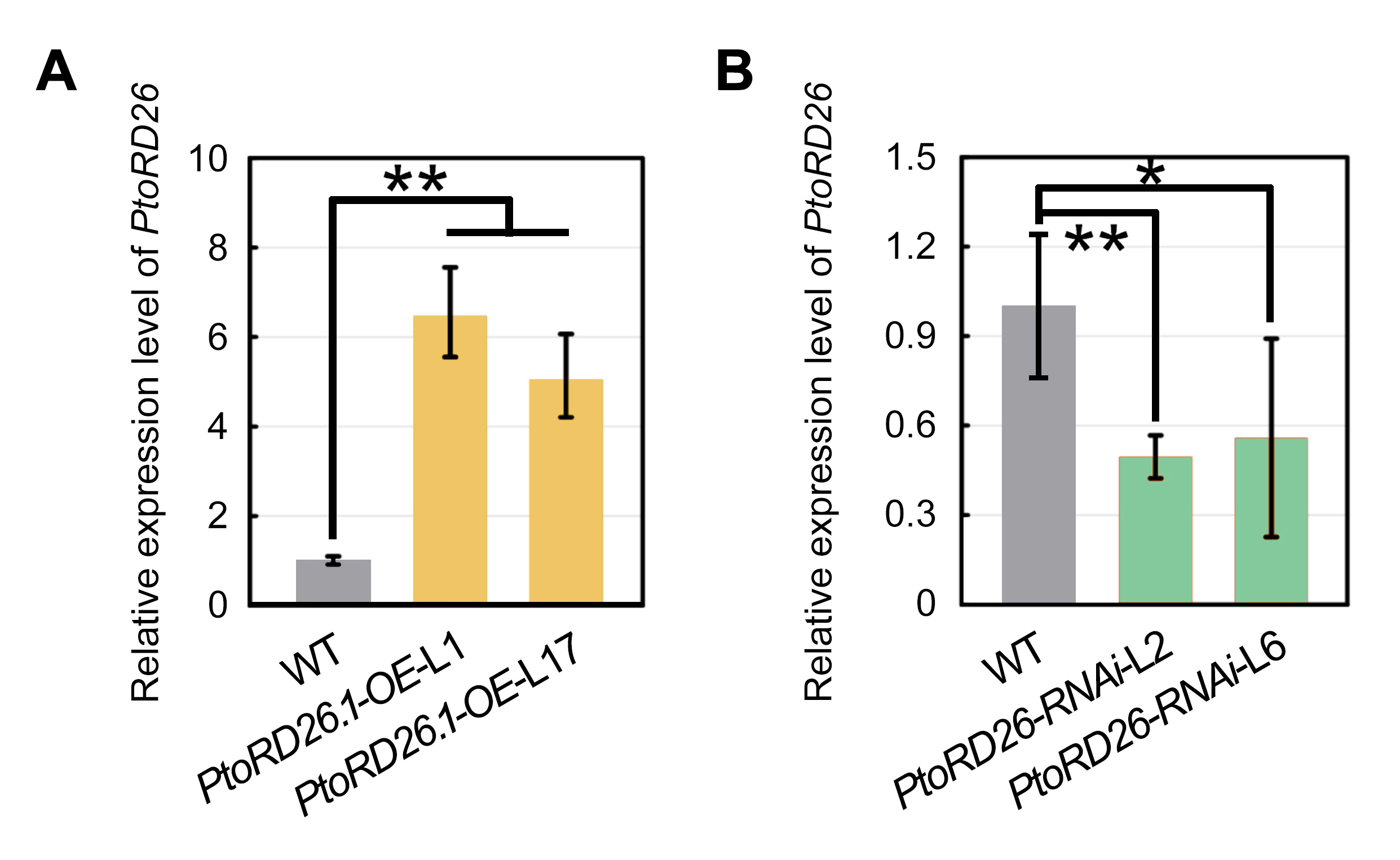


**Fig. S5. Determination of *PtoRD26* expression level in the *PtoRD26.1* overexpression and RNAi lines by qPCR. A** The expression level of *PtoRD26* in the *PtoRD26.1*-*OE*-L1 and L17 compared to WT. **B** The expression level of *PtoRD26* in *PtoRD26-RNAi*-L2 and L6 compared to WT. The means ± s.d. of all data from at least three biological replicates are shown. Asterisks indicate statistically significant differences (two-sided Student’s t-test, **P < 0.01, *P < 0.05).


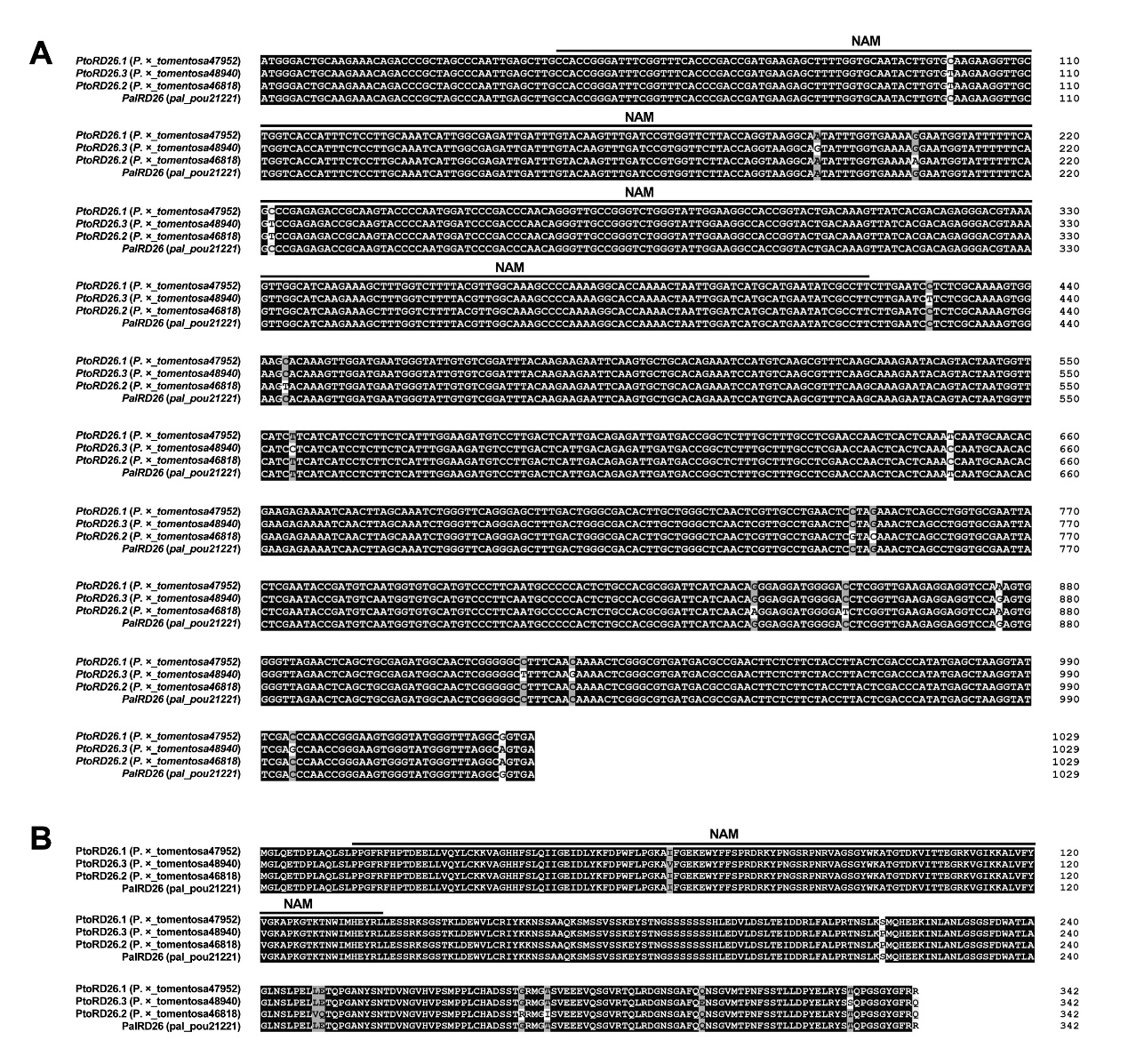


**Fig. S6.** **The sequence alignments of three *PtoRD26* alleles in the triploid poplars and their ortholog in *P. alba* var. *pyramidalis*.** **A** An alignments of coding sequences. The coding region of NAM domain is indicated. **B** A peptide sequence alignment. The NAM domain region is marked.


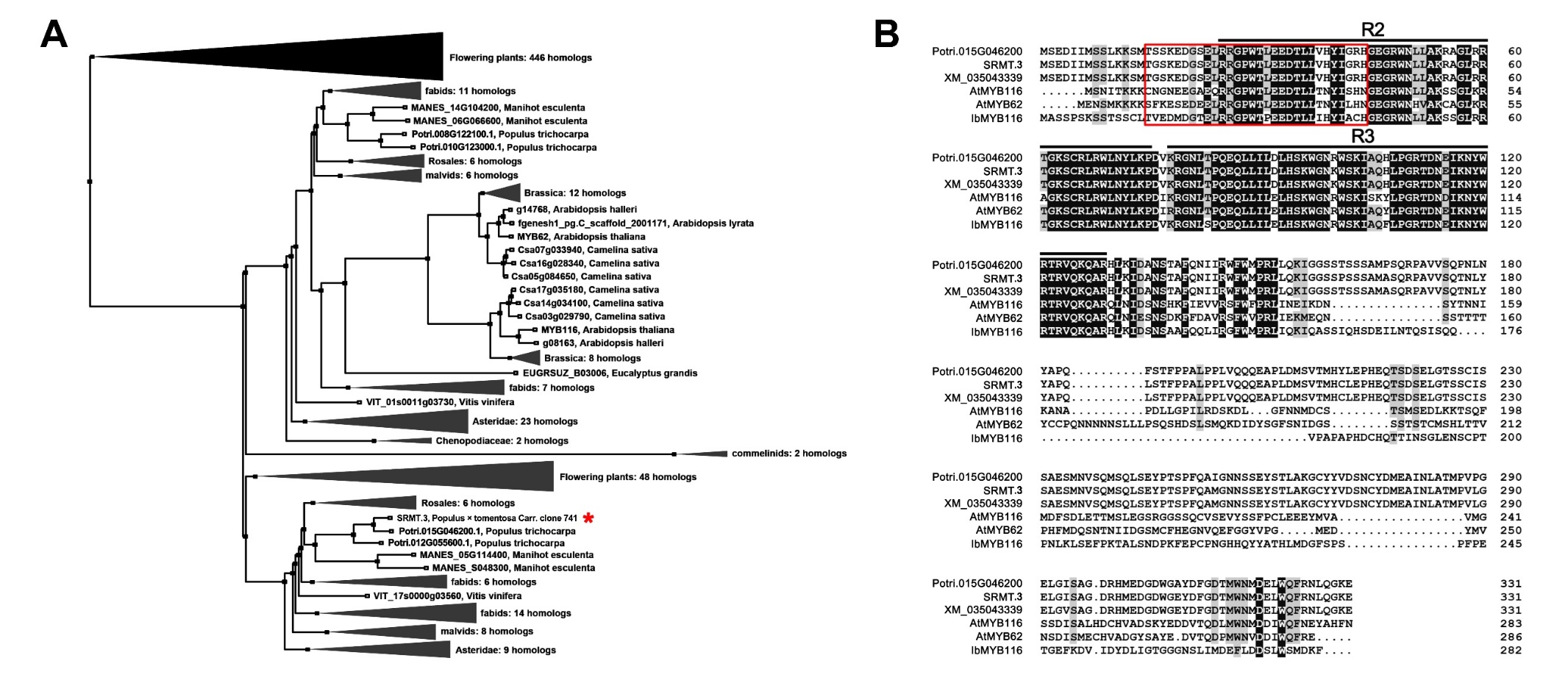


**Fig. S7. The phylogenetic relationship and peptide sequence of SRMT.** **A** A phylogenetic tree of SRMT homologs from various species. The red star indicates SRMT.3 from triploid Chinese white poplar. **B** The peptide sequence alignment of SRMT.3 and its homologs from various species.


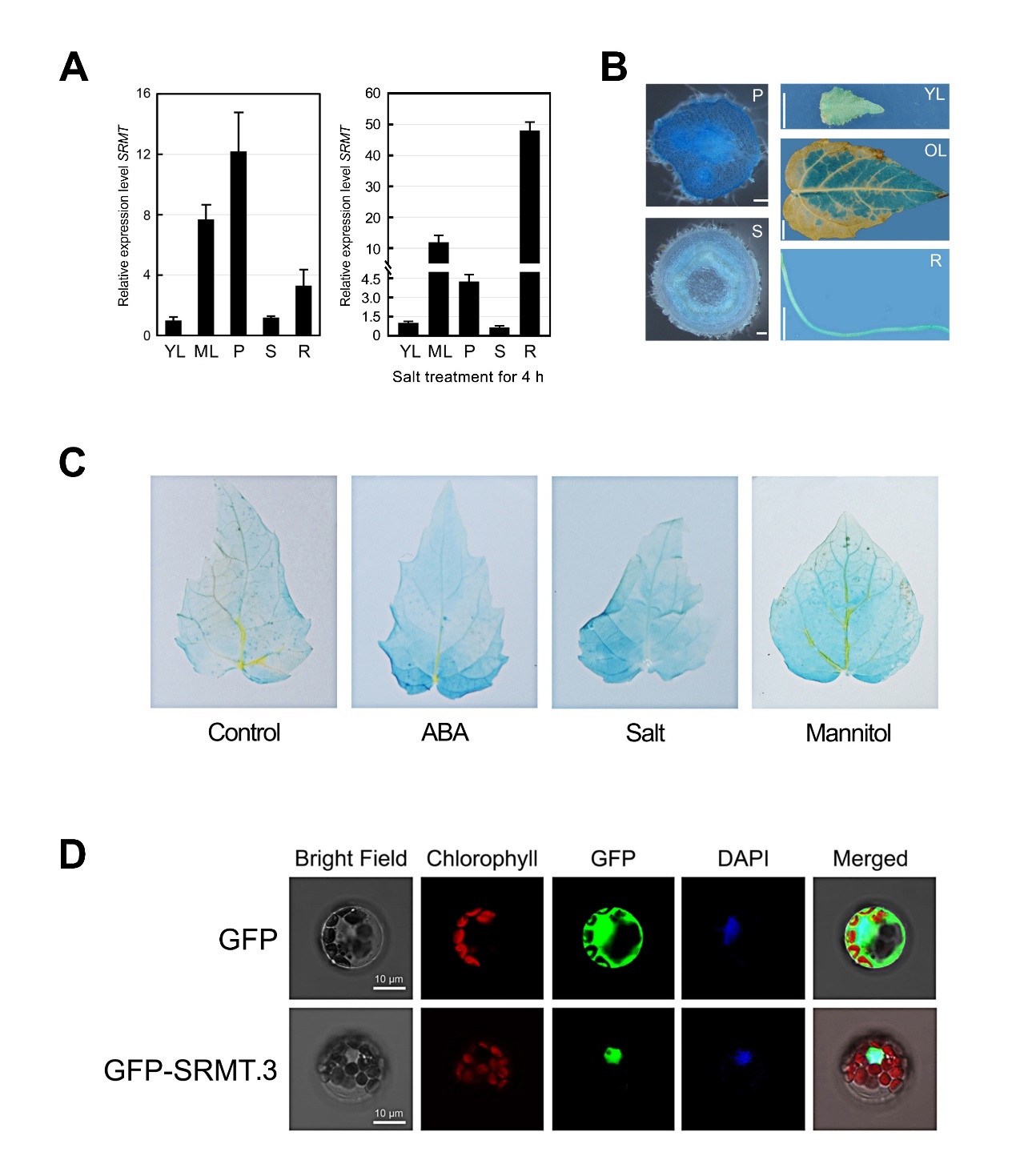


**Fig. S8. Tissue expression pattern analysis of *SRMT* in** **the triploid Chinese white poplars under normal condition and after salt treatment for 4 hours.** **A** The qPCR revealed the transcriptional abundance of *SRMT* in multiple tissues of the triploid Chinese white poplars with or without salt stress, including first expansion leaf (young leaf, YL), the 5^th^ node leaf (mature leaf, ML), petiole (P), stem (S), and total root (R). **B** The GUS staining shows the transcription level of the *β-glucuronidase* (*uidA*) driven by the promoter of *SRMT.3* in various tissues. There were three independent transgenic lines used in the GUS staining assay and the representative results from one experiment are shown. **C** The GUS staining indicates that the *uidA* reporter gene is driven by the 1.5 kb promoter region of *SRMT.3* in transgenic poplar. The leaves at the third internode from the same transgenic lines in WPM medium were detached and submerged in the WPM liquid medium containing 10 μM ABA, 150 mM NaCl and 200 mM mannitol for 6 h. The leaves submerged in the WPM medium were used as controls. **D** The GFP-SRMT.3 fused protein was localized in the nucleus, while free GFP was localized both in cytoplasm and nucleus. These protoplasts were isolated from mesophyll of *P.× tomentosa* Carr. clone 741.


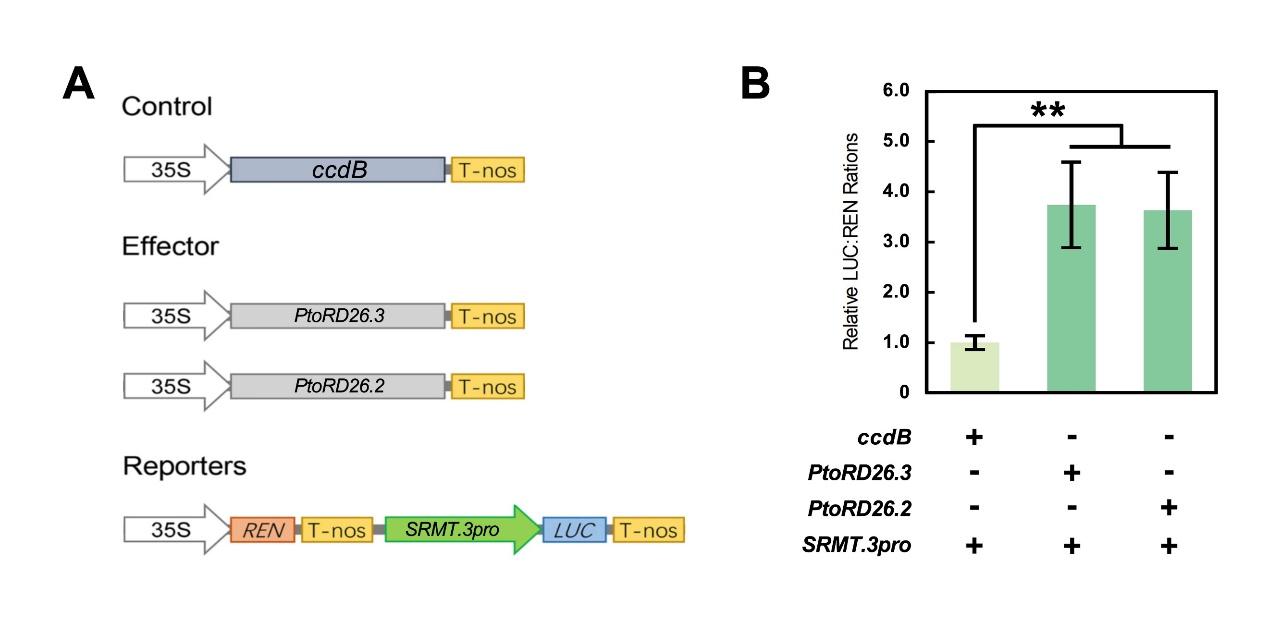


**Fig. S9. Both PtoRD26.2 and PtoRD26.3 up-regulated the expression of *LUC* driven by *SRMT.3pro* promoter in tobacco leaves.** **A** The constructs of the effector and reporter in the dual-luciferase assay. **B** Co-expressing *PtoRD26.2* or *PtoRD26.3* enhanced fluorescence intensity of LUC driven by *SRMT.3pro* promoter compared to the control, respectively. Asterisks indicate statistically significant differences (two-sided Student’s t-test, **P < 0.01).


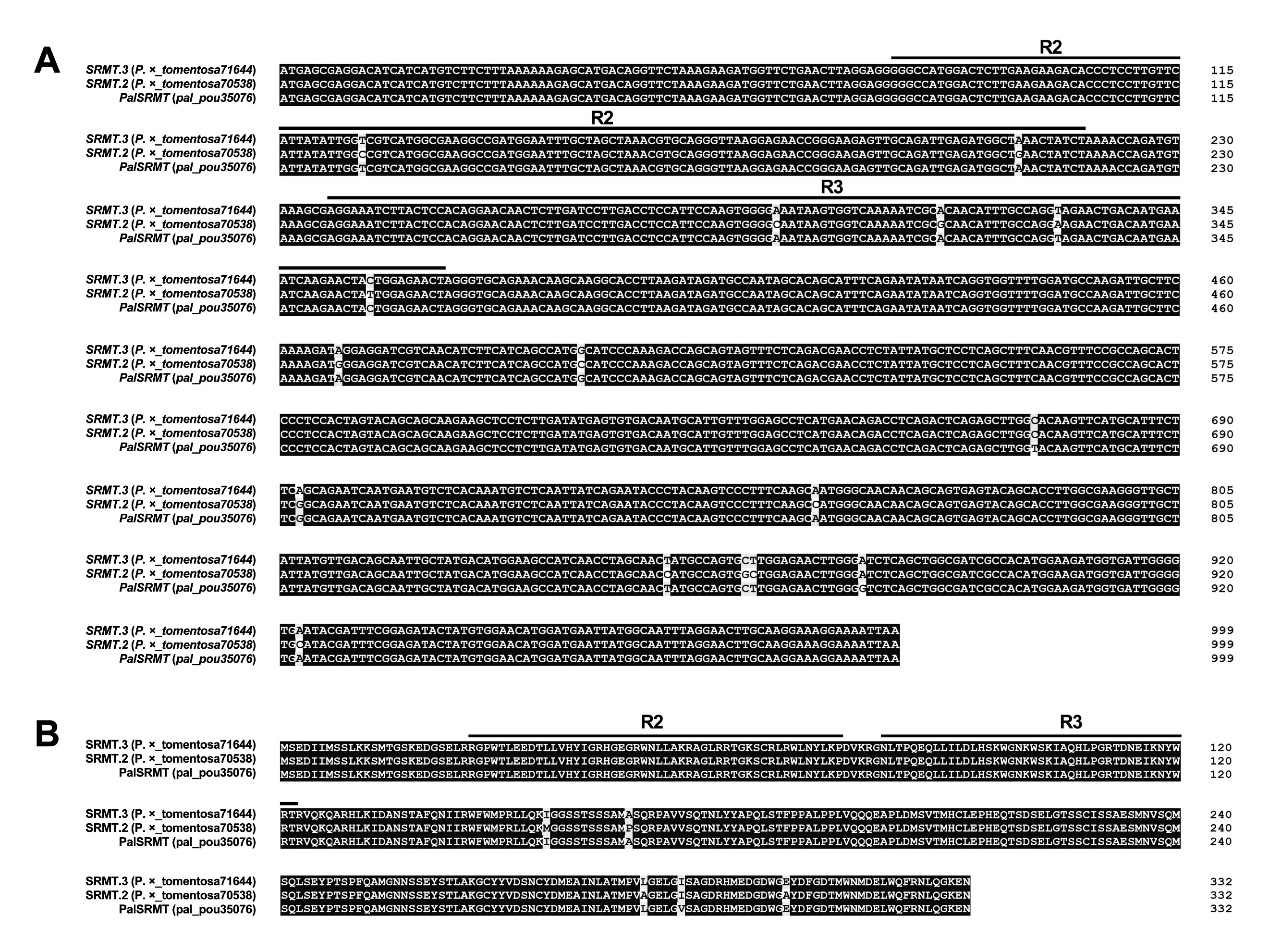


**Fig. S10. The sequence alignments of two *SRMT* alleles in the triploid poplars and their ortholog in *P. alba* var. *pyramidalis*. A** An alignments of coding sequences. The coding region of R2R3 MYB domain is indicated. **B** A peptide sequence alignment. The R2R3 MYB domain region is marked.


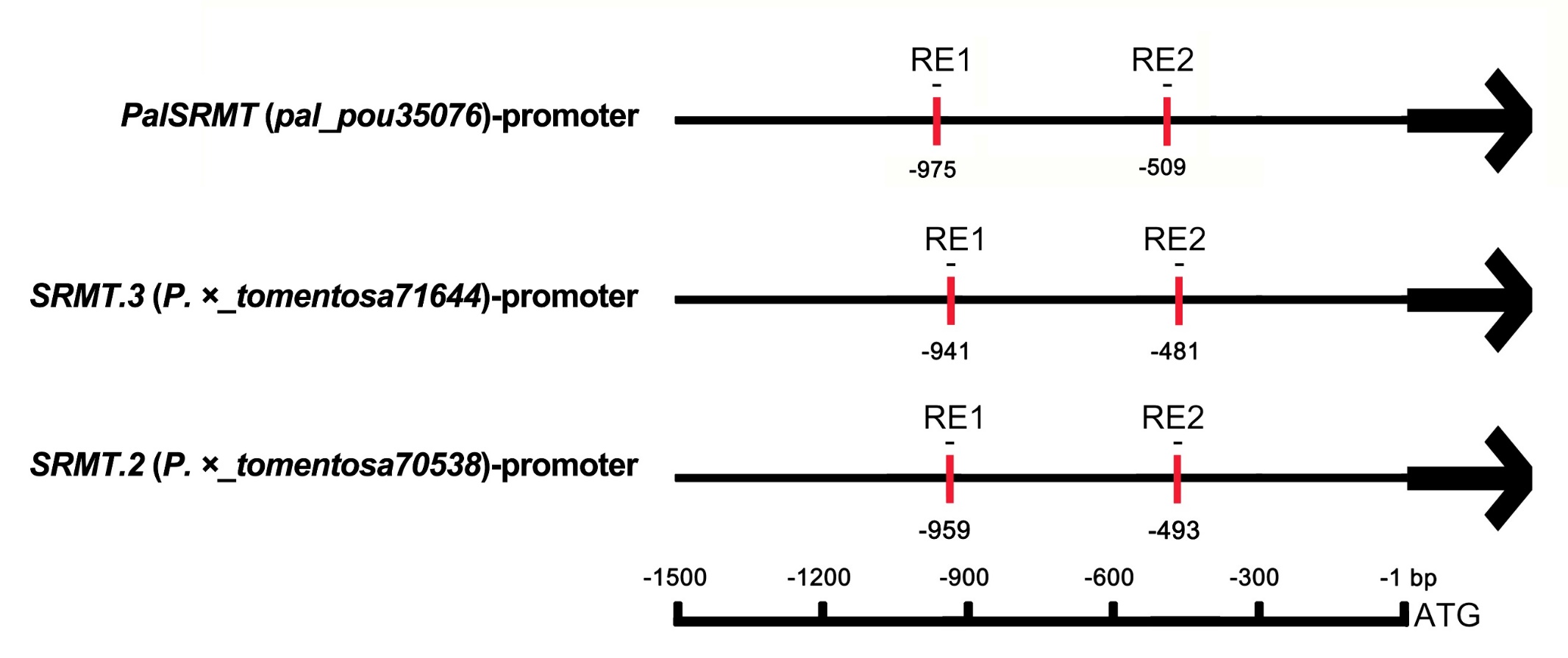


**Fig. S11. The distributions of PtoRD26 binding elements in the promoters of *SRMT* alleles in this triploid poplar and in the promoter of the ortholog in *P. alba* var. *pyramidalis*.** The potential PtoRD26 binding elements (red rectangles) and their locations are indicated. These *cis*-elements are conserved in these three promoters.


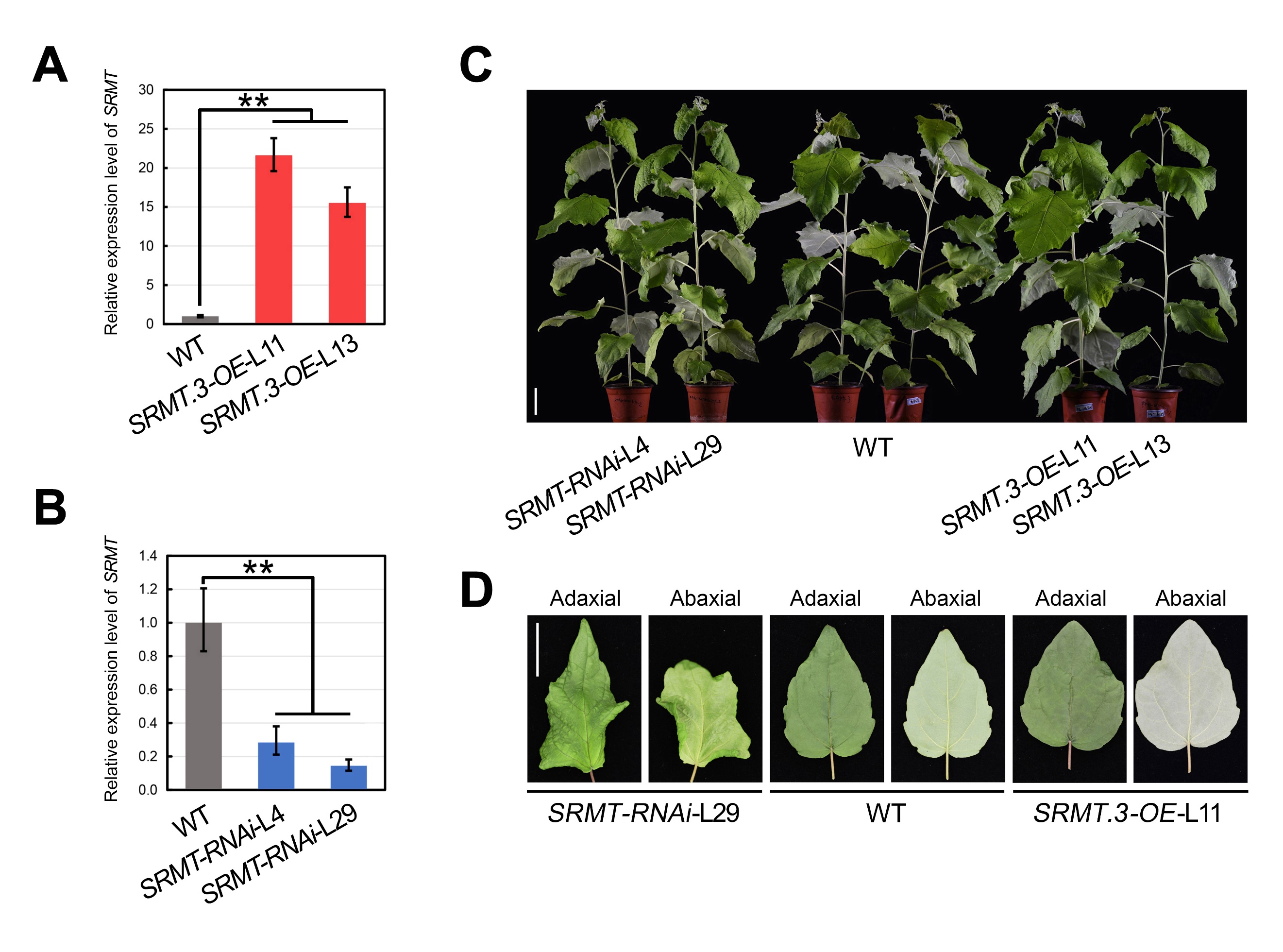


**Fig. S12. Identification of morphology of *SRMT* transgenic poplars.** **A** The expression level of *SRMT* in the *SRMT.3-OE* lines (L11 and L13) were determined by qPCR. **B** The expression levels of *SRMT* in the *SRMT-RNAi* lines (L4 and L29) were determined by qPCR. Asterisks indicate statistically significant differences (two-sided Student’s t-test, **P < 0.01). **C** The morphology of the three-month-old *SRMT.3-OE*, *SRMT-RNAi* and WT poplars grown in the greenhouse. **D** The adaxial and abaxial morphology of leaves at the 5^th^ node of *SRMT.3-OE*, *SRMT-RNAi* and WT poplars, respectively. The leaves of *SRMT-RNAi* lines were crimped, especially the mature and old leaves.


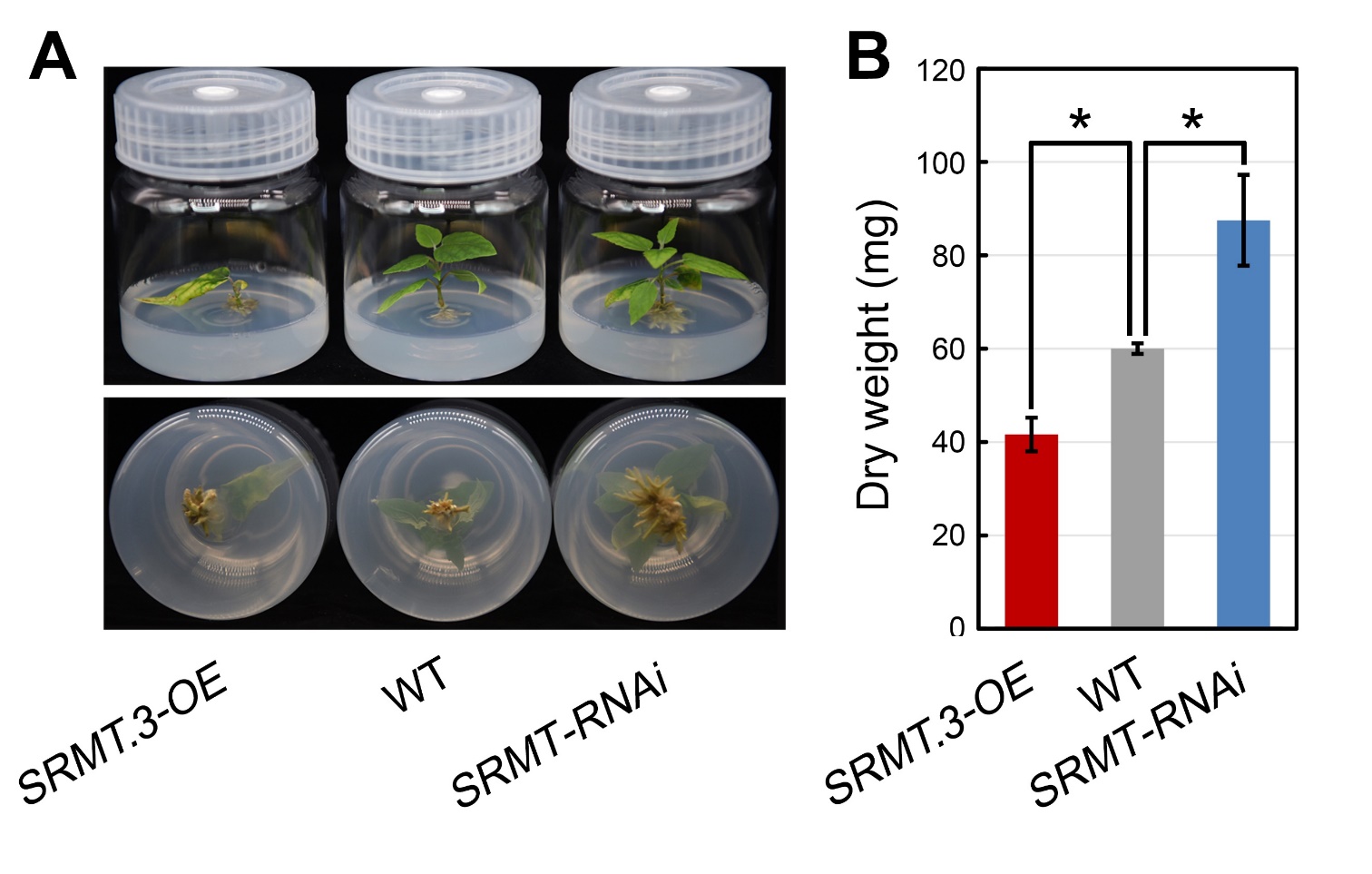


**Fig. S13. Sensitivity of *SRMT.3-OE*, *SRMT-RNAi* and WT poplar cuttings to ABA treatment.** **A** The morphology of sterile cuttings from *SRMT.3-OE*, *SRMT-RNAi* and WT poplars grown in WPM solid medium supplemented with 5 μM ABA for 40 days. **B** The dry weight of the poplar cuttings from *SRMT.3-OE*, *SRMT-RNAi* and WT after ABA treatment in (**A**). The means ± s.d. of all data from at least three biological replicates are shown. Asterisks indicate statistically significant differences (two-sided Student’s t-test, *P < 0.05).


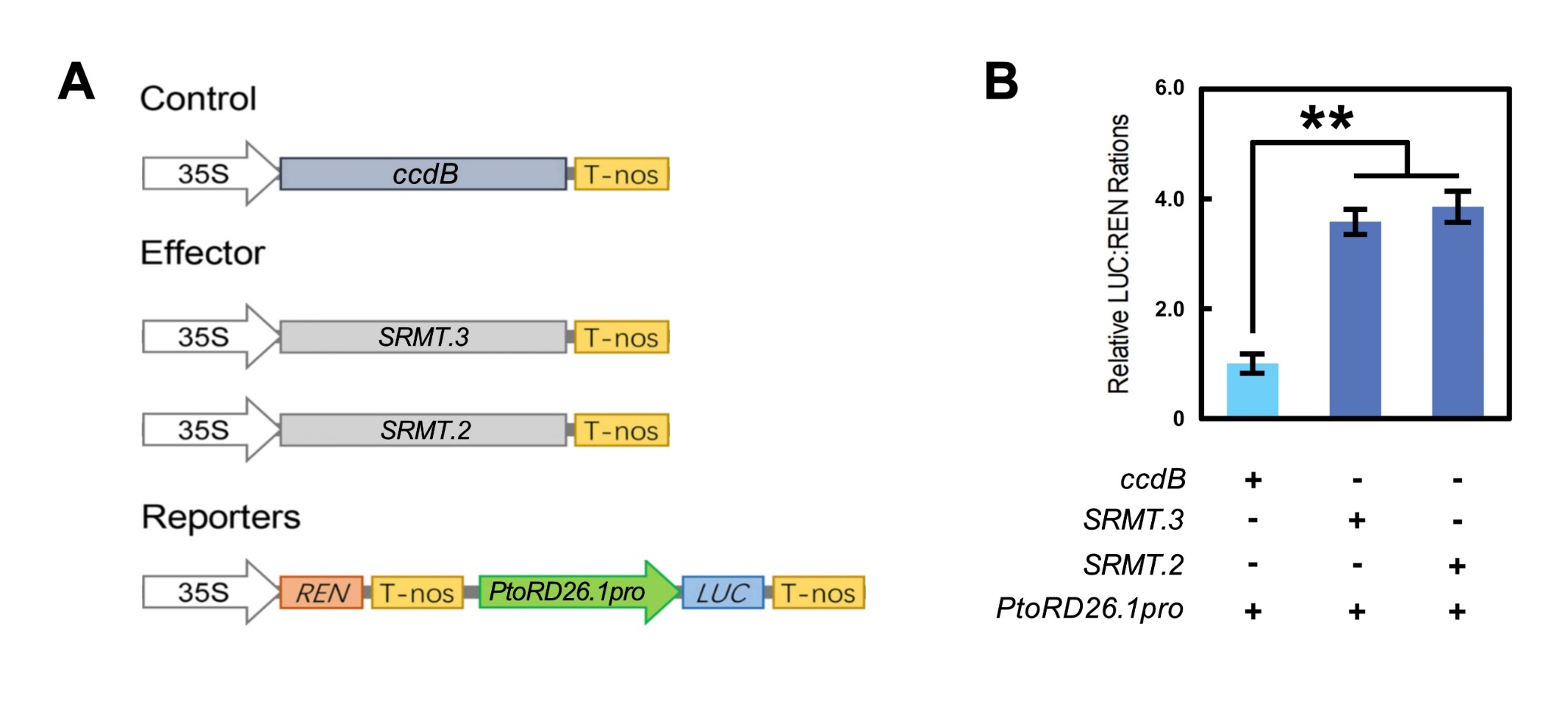


**Fig. S14. Both SRMT.2 and SRMT.3 up-regulated the expression of *LUC* driven by *PtoRD26.1* promoter in tobacco leaves.** **A** The constructs of the effector and reporter in the dual-luciferase assay. **B** Co-expressing *SRMT.2* or *SRMT.3* enhanced fluorescence intensity of *LUC* driven by *PtoRD26.1* promoter compared to the control, respectively. Asterisks indicate statistically significant differences (two-sided Student’s t-test, **P < 0.01).


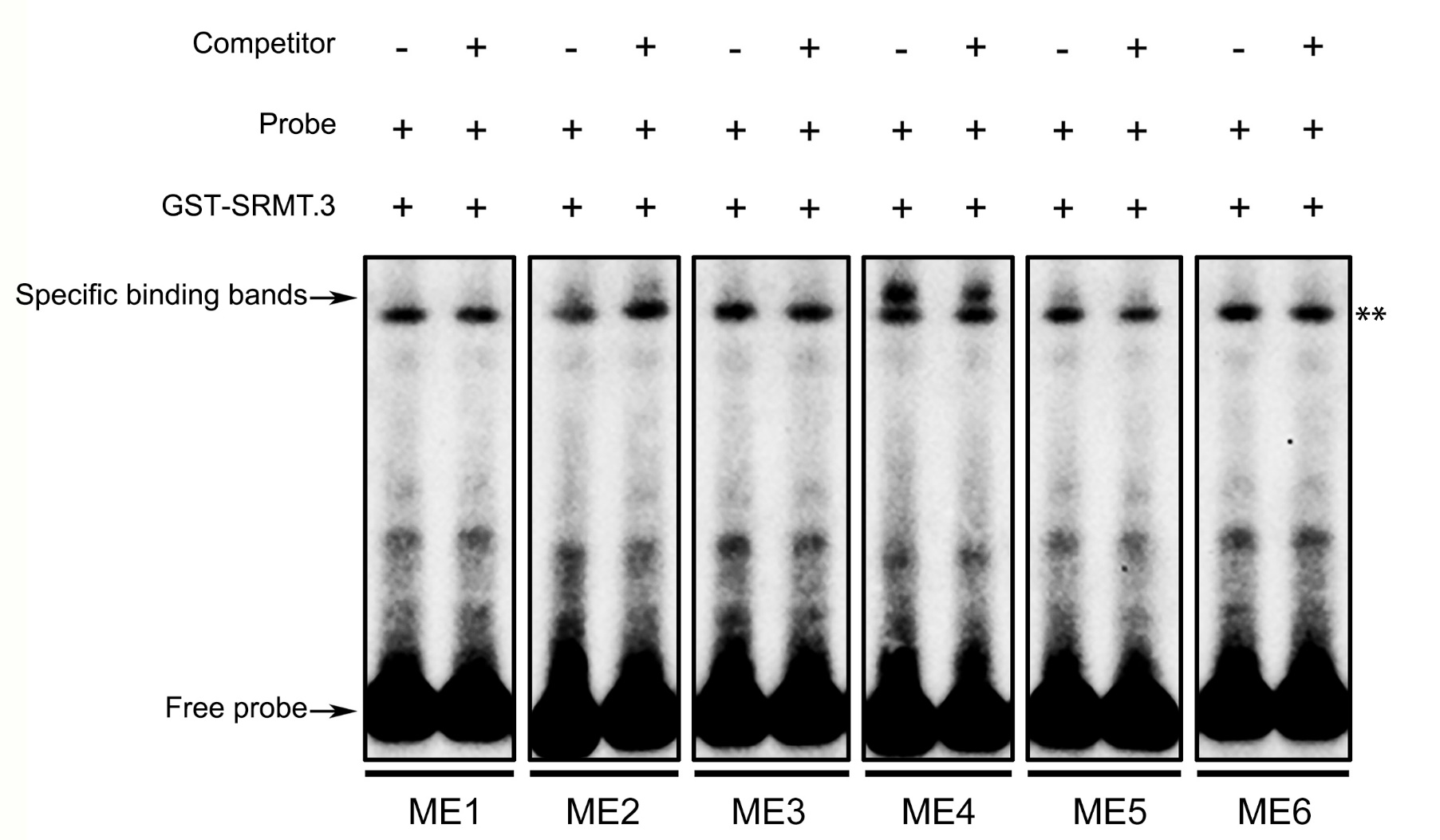


**Fig. S15. EMSA indicated that the binding sites of SRMT.3 in the** **1.5 kb promoter region of *PtoRD26.1*.** There were 6 MEs (ME1-ME6) in the 1.5 kb region promoter of *PtoRD26.1*. ME2, ME4 and ME5 can be bound by GST-SRMT.3 and competitive by cold probes. The bands of specific binding probes and the free probes are indicated by arrows, the non-specific bands are highlighted by stars.


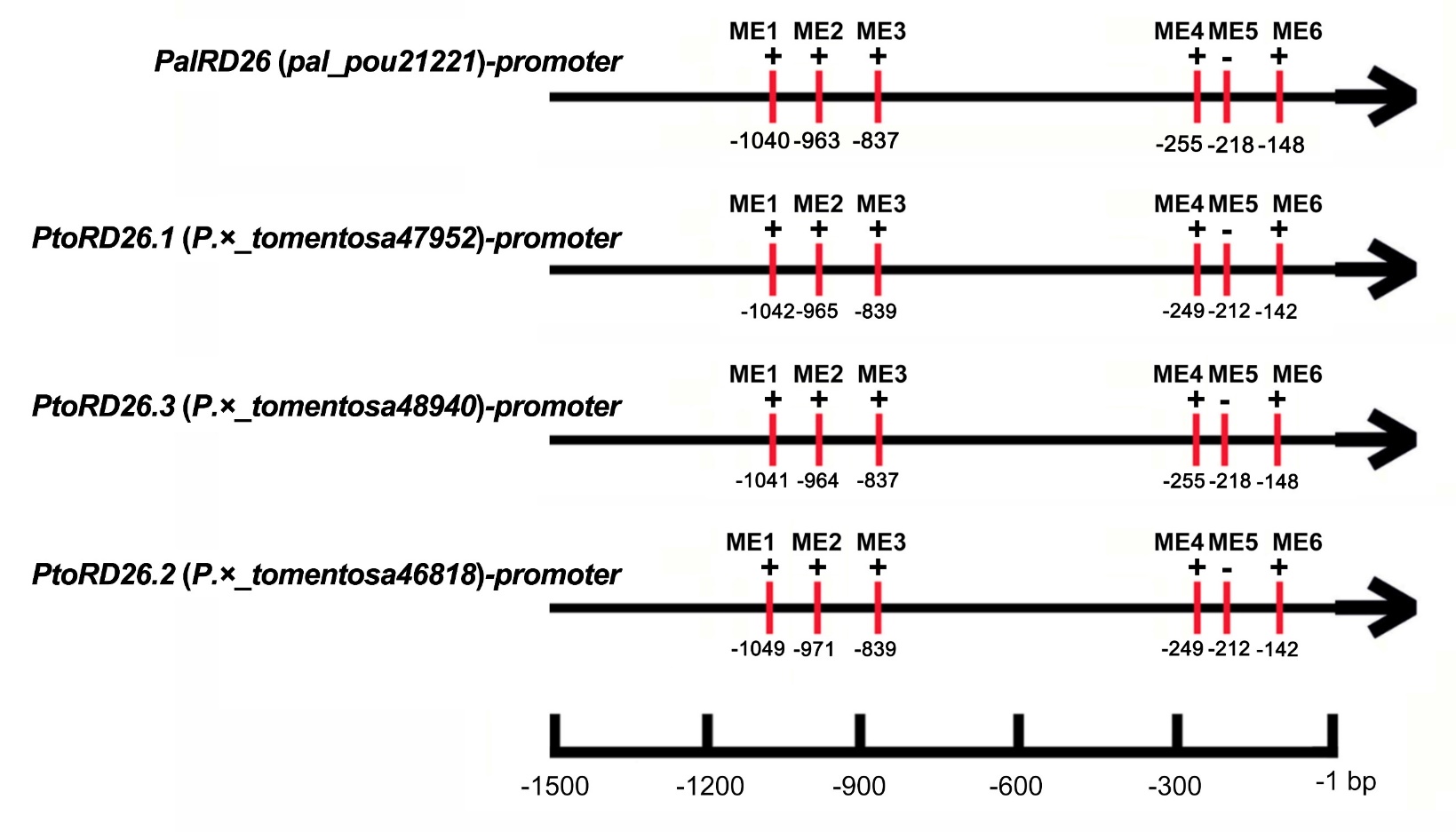


**Fig. S16. The distributions of SRMT binding elements in the promoters of *PtoRD26* alleles in this triploid poplar and in the promoter of the ortholog in *P. alba* var. *pyramidalis*.** The potential SRMT binding elements (red rectangles) and their locations are indicated. These *cis*-elements are conserved in these promoters.


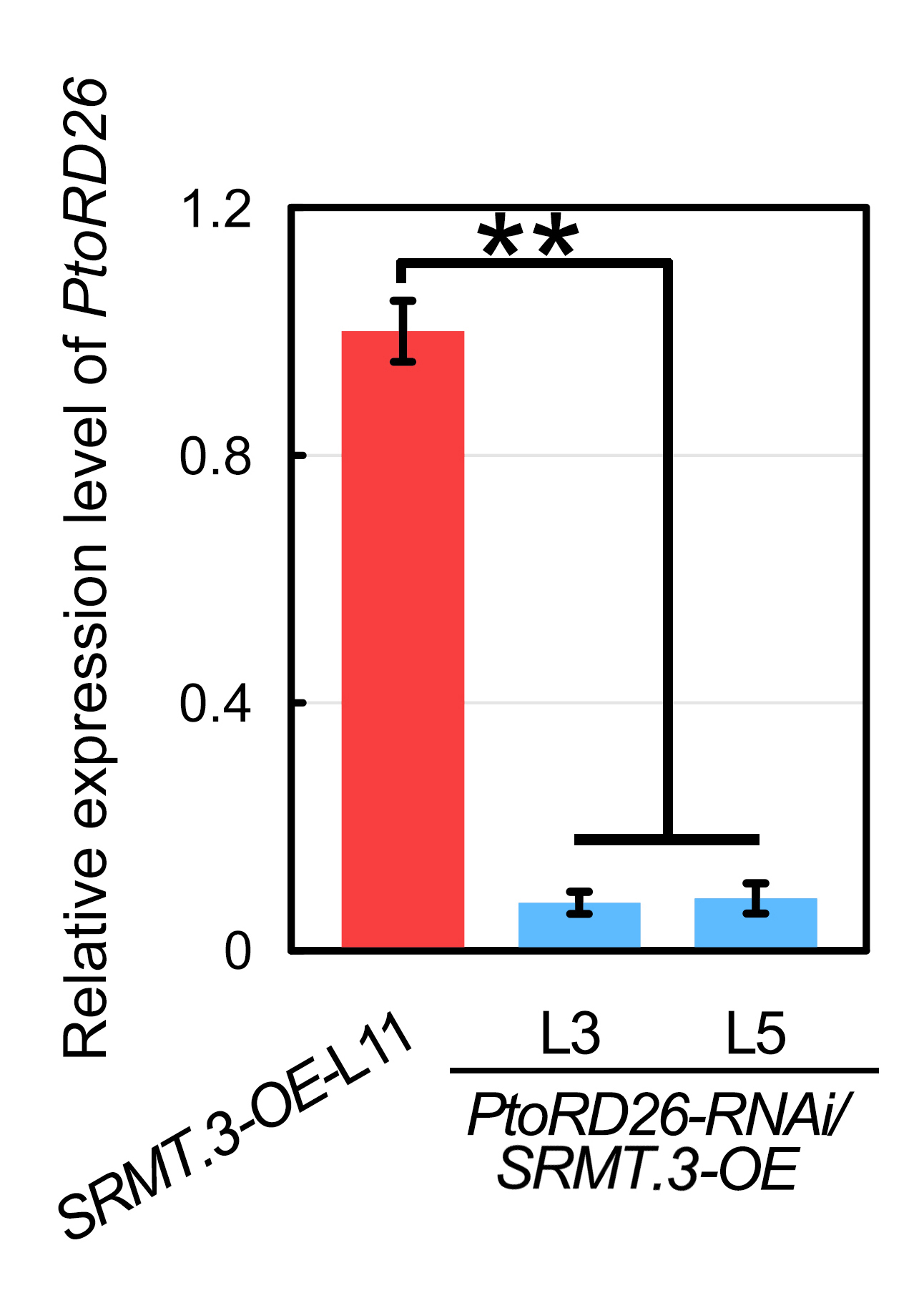


**Fig.** **S17. The determination of the *PtoRD26* expression level in the double transgenic poplars (*****PtoRD26-RNAi/SRMT.3-OE*).** The double transgenic poplar lines (L3 and L5) were generated by knocking-down *PtoRD26* with the RNAi method in the background of *SRMT.3*-*OE*-L11. The expression level of *PtoRD26* was significantly reduced in the transgenic lines L3 and L5 of *PtoRD26-RNAi/SRMT.3-OE* demonstrating that the *PtoRD26-RNAi* construct worked. Asterisks indicate statistically significant differences (two-sided Student’s t-test, **P < 0.01).


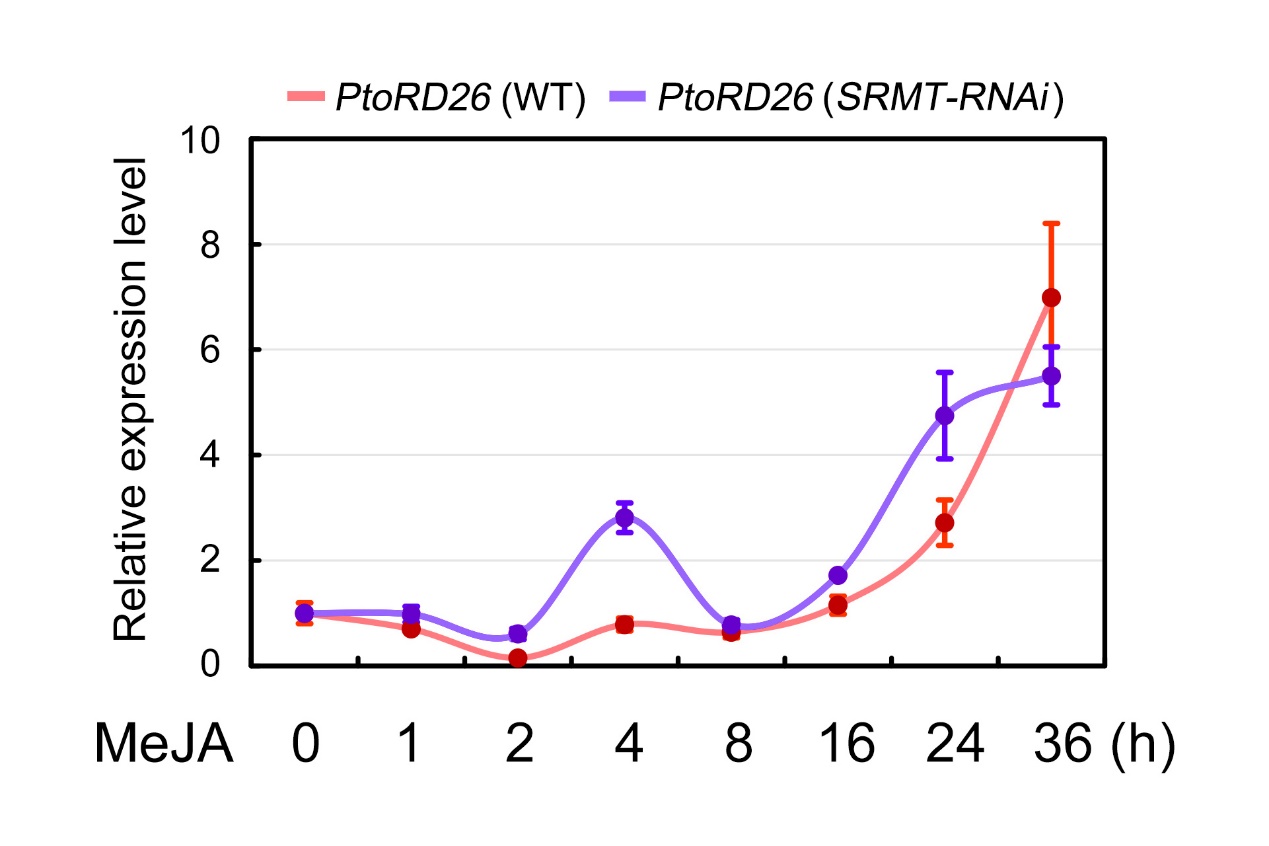


**Fig. S18. The temporal expression pattern of *PtoRD26* in response to MeJA was independent on *SRMT*.** The expression level of *PtoRD26* in WT and *SRMT-RNAi* poplar leaves at 0, 1, 2, 4, 8, 16, 24, 36 h after 30 μM MeJA treatment was determined by qPCR.


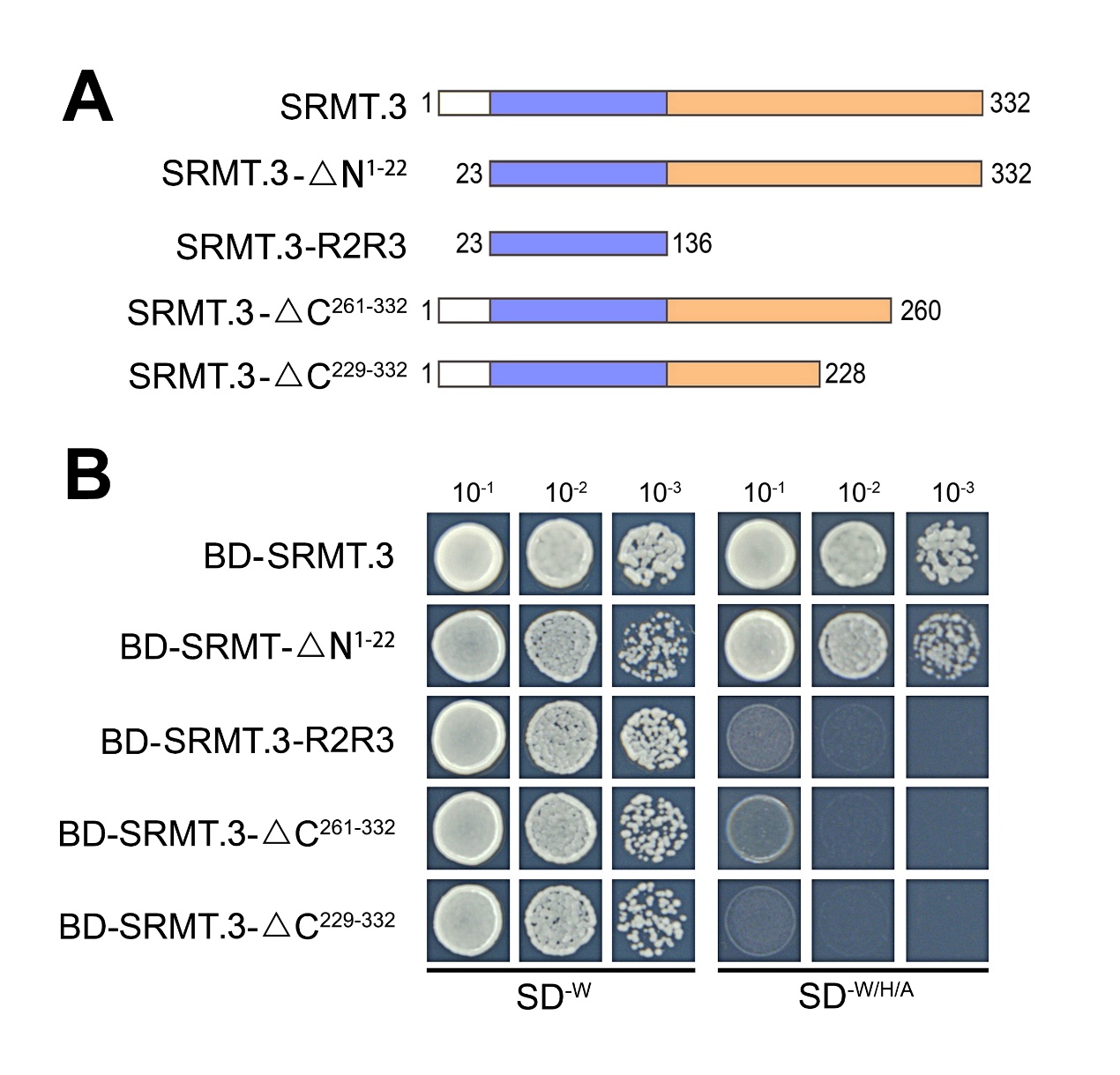


**Fig. S19. Self-activation activity identification of SRMT.3 in yeast strain AH109. A** The various truncated SRMT.3 proteins, including the full-length SRMT.3, SRMT.3-△N^1-22^, SRMT.3-R2R3 domain, SRMT.3-△C^261-332^ and SRMT.3-△C^229-332^. The coding sequences of these truncated SRMT.3 proteins were ligated in the pGBKT7 vector (BD). **B** All constructed vectors were introduced into the yeast and accessed by the self-activation activity of the positive transformants on the screening medium lacking W, H and A (SD^-W/H/A^). The positive transformants containing *BD-SRMT.3-R2R3*, *BD-SRMT.3-△C^261-332^* and *BD-SRMT.3-△C^229-332^* constructs were mortal on the SD^-W/H/A^ medium, indicating that they had no self-activation activity.


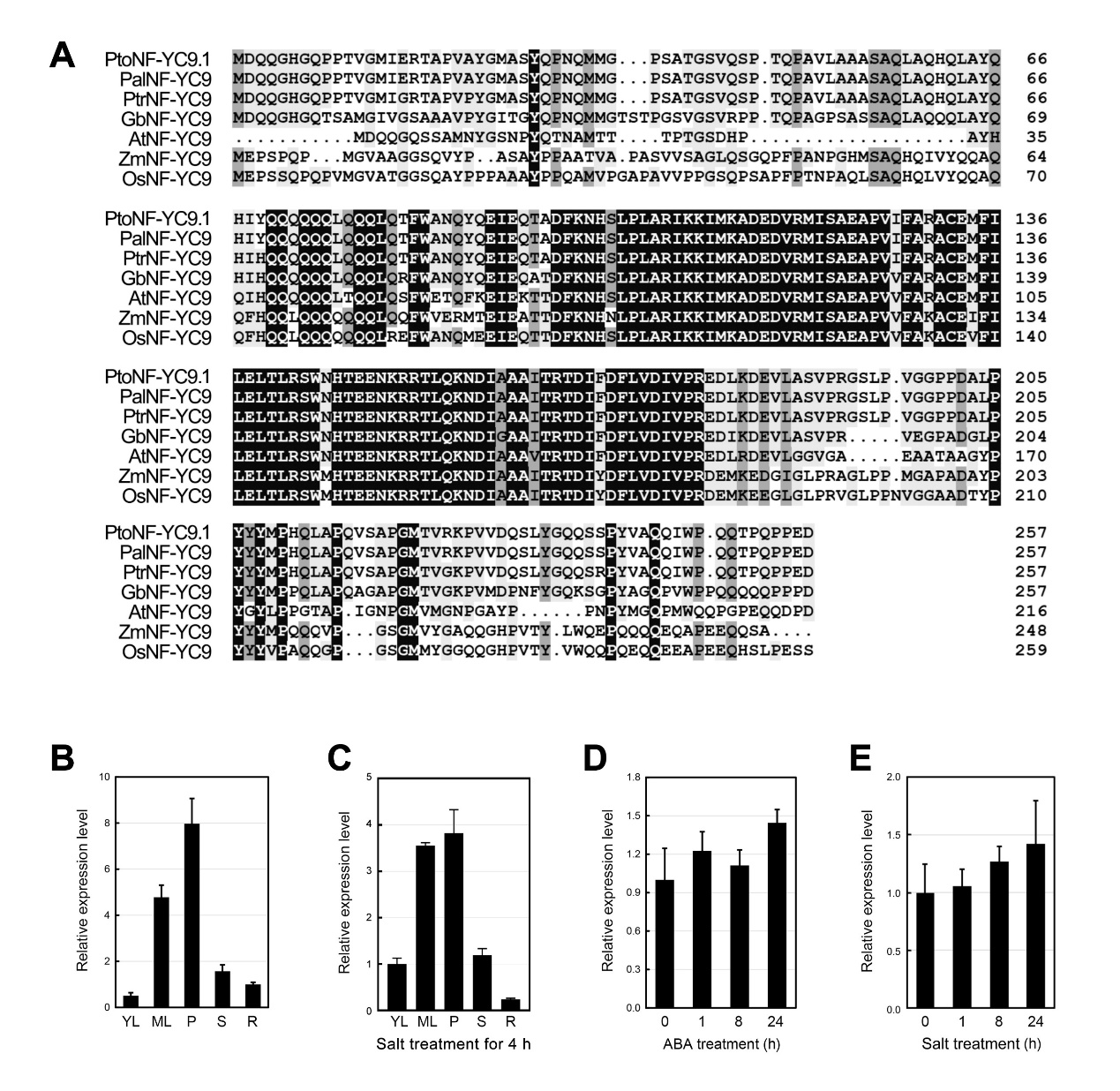


**Fig. S20. The peptide sequence of NF-YC9 from various species and the spatio-temporal expression pattern of *PtoNF-YC9.1*.** **A** The peptide sequence alignments of NF-YC9 in the triploid Chinese white poplar (P.x_tomentosa23609), *P. trichocarpa* (Potri.008G203500.1), *P. alba* (Genbank accession: XP_034889600.1), cotton (Genbank accession: KAB2092491.1), *Arabidopsis* (AT1G54830.1), corn (Genbank accession: NP_001136950.1) and rice (Genbank accession: XP_015627520.1). **B** The qPCR analysis revealed the transcriptional abundance of *PtoNF-YC9.1* in various tissues, including first expansion leaf (young leaf, YL), 5^th^ node leaf (mature leaf, ML), petiole (P), stem (S), and total root (R). **C** The expression level of *PtoNF-YC9* in various salt-stressed tissues. **D** The expression levels of *PtoNF-YC9* at 0, 1, 8 and 24 h after ABA treatment. **E** The expression levels of *PtoNF-YC9* at 0, 1, 8 and 24 h after salt treatment.


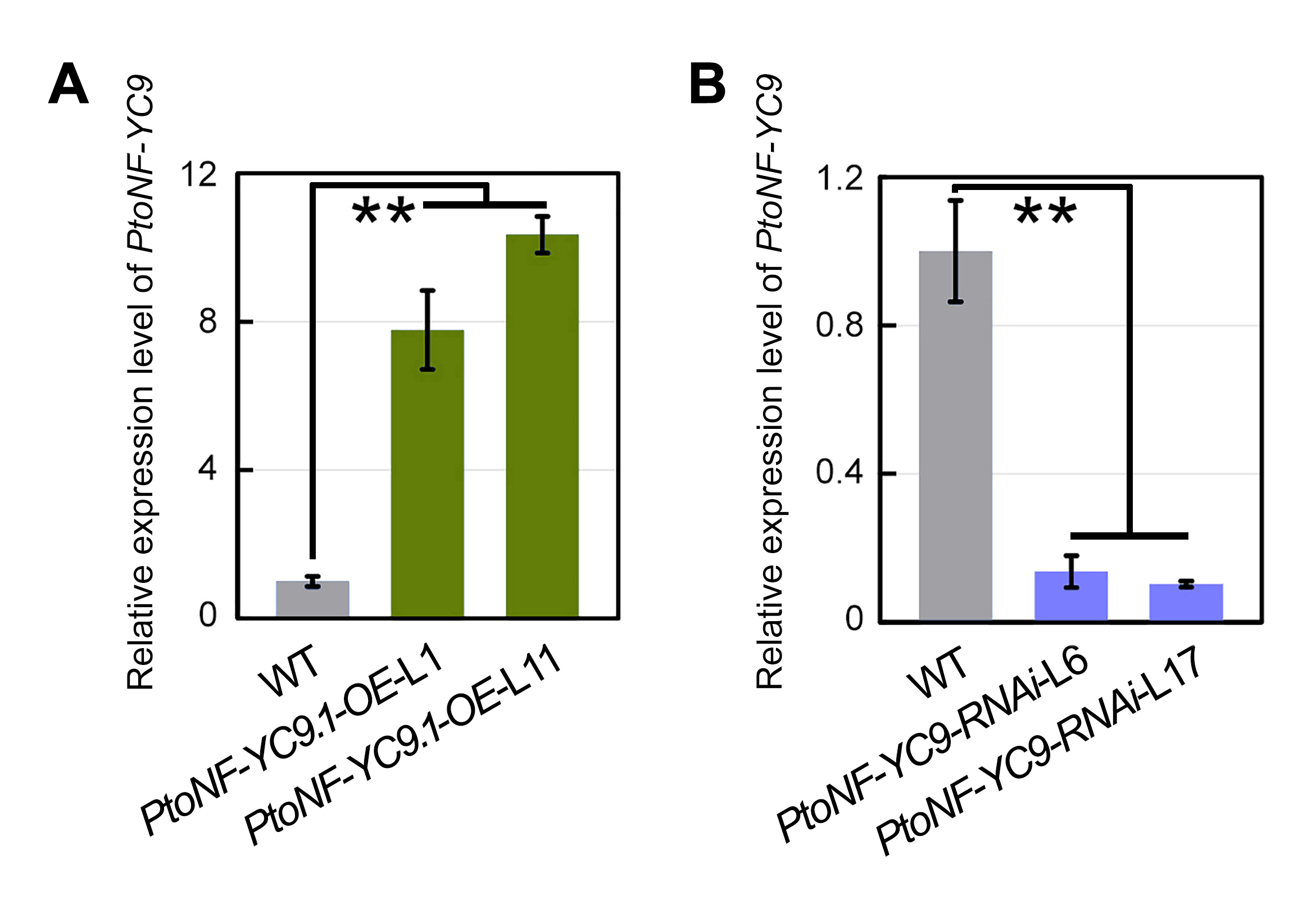


**Fig. S21. The expression levels of *PtoNF-YC9* in the *PtoNF-YC9.1* overexpression and RNAi poplars by qPCR. A** The expression level of *PtoNF-YC9* in two independent lines overexpressing *PtoNF-YC9.1* (*PtoNF-YC9.1-OE-*L1 and L11) were detected by qPCR. **B** The expression levels of *PtoNF-YC9* in two independent *PtoNF-YC9-RNAi* lines (L6 and L17) were detected by qPCR. Asterisks indicate statistically significant differences (two-sided Student’s t-test, **P < 0.01).


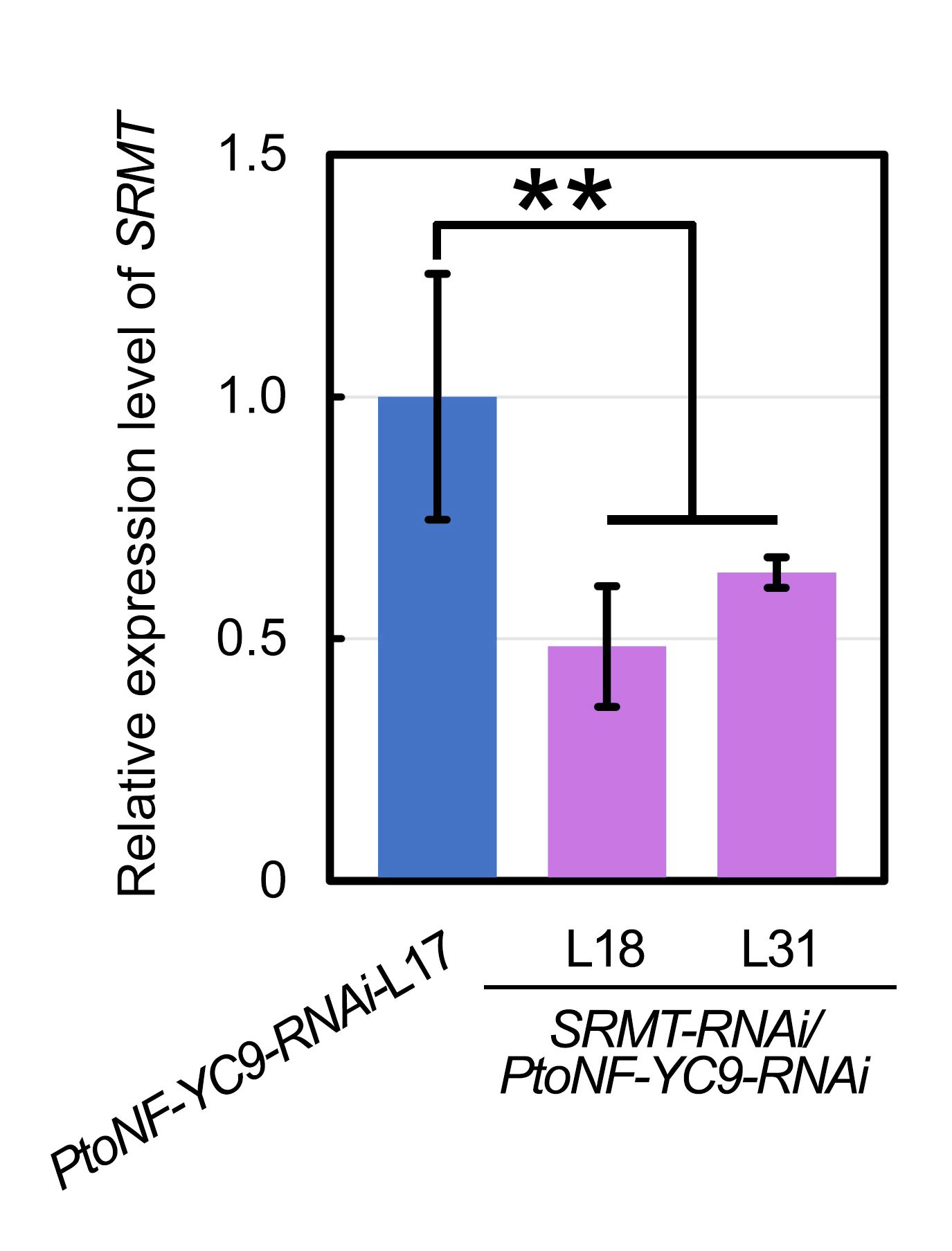


**Fig. S22. The determination of the *SRMT* expression level in the double knocking-down poplars (*SRMT-RNAi/PtoNF-YC9-RNAi*).** The double knocking-down poplars (L18 and L31) were generated through RNAi of *SRMT* in the background of *PtoNF-YC9*-*RNAi*-L17. The expression levels of *SRMT* were significantly reduced in the L18 and L31 of *SRMT-RNAi/PtoNF-YC9-RNAi*. These results were derived from two independent lines. Asterisks indicate statistically significant differences (two-sided Student’s t-test, **P < 0.01).


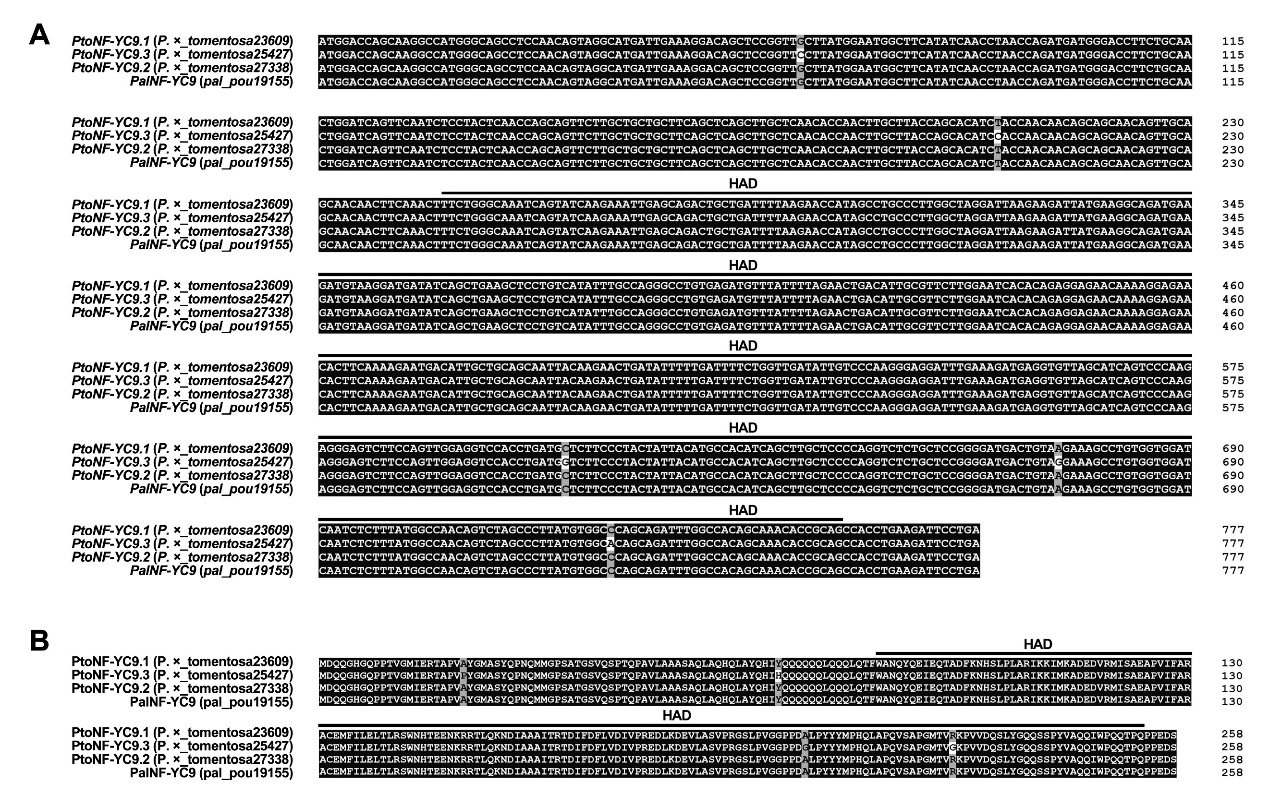


**Fig. S23. The sequence alignments of three *PtoNF-YC9* alleles in the triploid poplars and their ortholog in *P. alba* var. *pyramidalis*.** **A** An alignments of coding sequences. The coding region of HAD domain is indicated. **B** A peptide sequence alignment. The HAD domain region is marked.


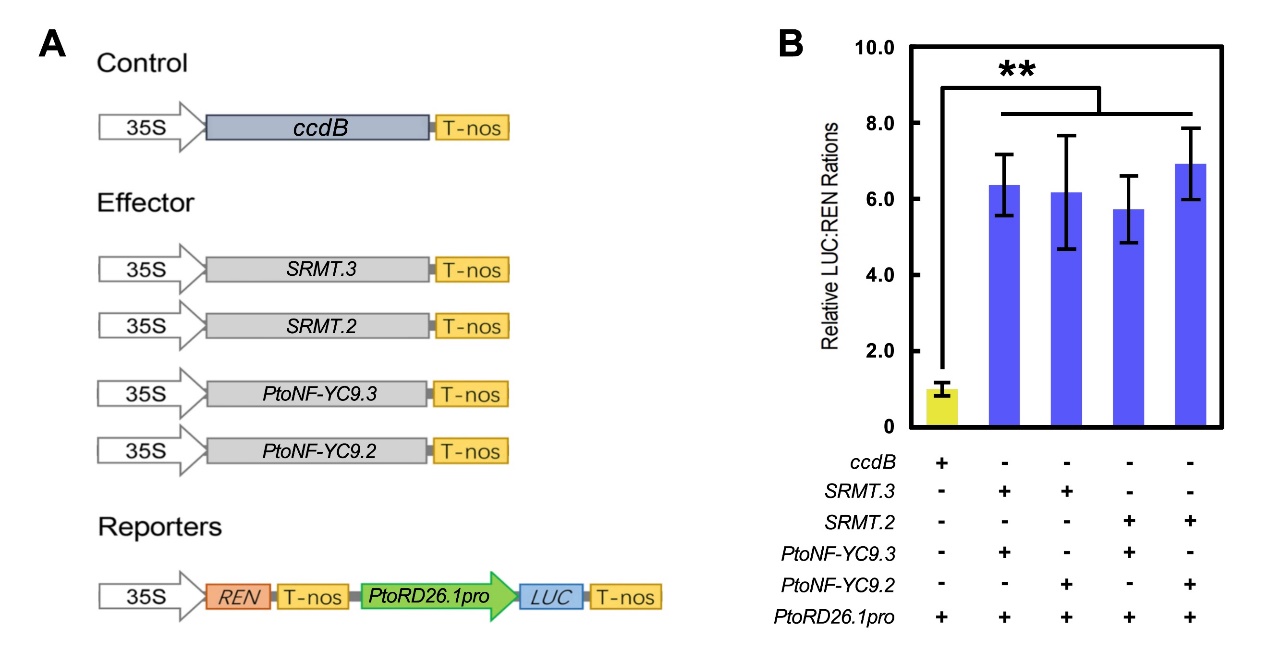


**Fig. S24. *SRMT* and *PtoNF-YC9* alleles were synergistically up-regulated the expression of *LUC* driven by *PtoRD26.1* promoter in tobacco leaves. A** The constructs of the effector and reporter in the dual-luciferase assay. **B** Co-expressing *SRMT.2* and *SRMT.3* with PtoNF-CY9.2 or PtoNF-CY9.3 enhanced fluorescence intensity of *LUC* driven by *PtoRD26.1* promoter compared to the control, respectively. Asterisks indicate statistically significant differences (two-sided Student’s t-test, **P < 0.01).


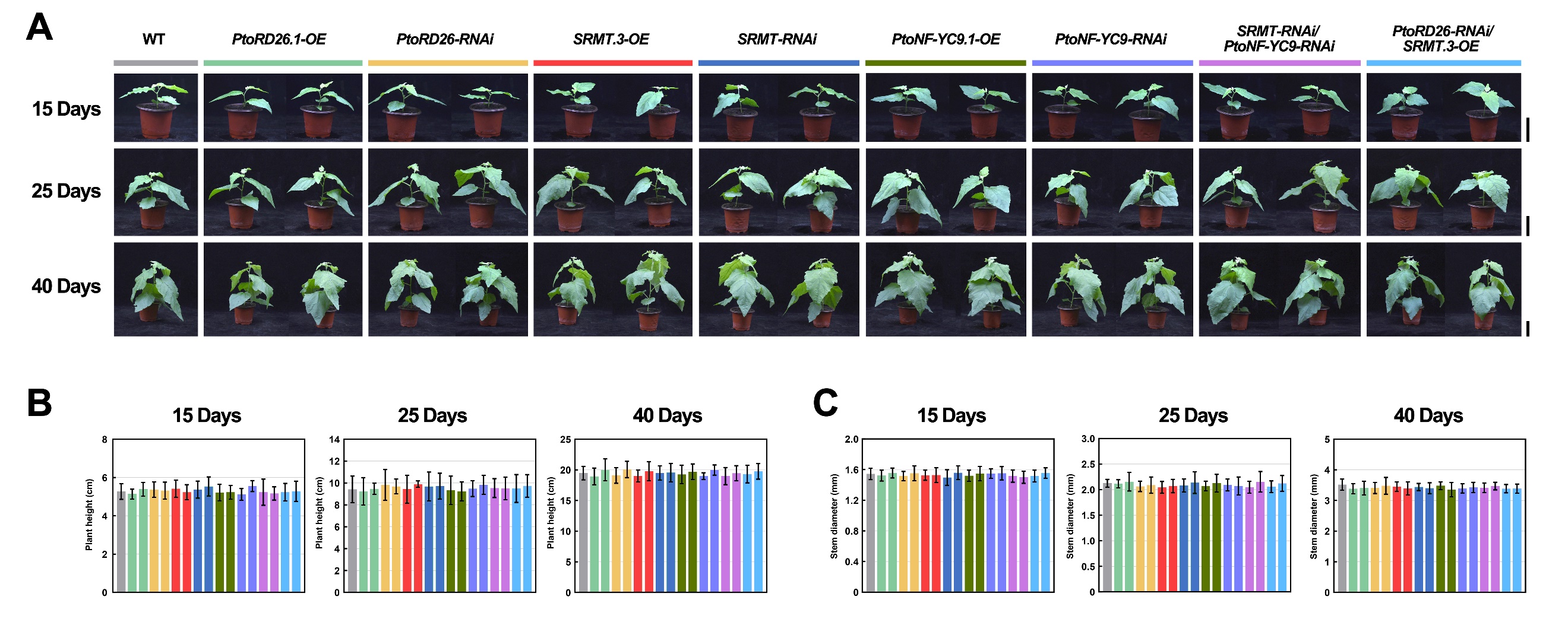


**Fig. S25. The plant height and stem diameter of all transgenic lines, including *PtoRD26.1-OE* (L1 and L17), *PtoRD26-RNAi* (L2 and L6), *SRMT.3-OE* (L11 and L13), *SRMT-RNAi* (L4 and L29), *PtoNF-YC9.1-OE* (L1 and L11), *PtoNF-YC9-RNAi* (L6 and L17), *SRMT-RNAi/PtoNF-YC9-RNAi* (L18 and L31) and *PtoRD26-RNAi/SRMT.3-OE* (L3 and L5) transgenic poplars at the 15 days, 25 days and 40 days after transplanting into soil, respectively. A** The growth status of poplars. The scales represent 5 cm. **B** The measurement of plants height. **C** The measurement of stem diameter. These data were collected from three plantlets of two independent lines of each genotype. There were no significant differences in plant height and stem diameter among different genotypical poplars.
