# Supplementary material for: PtoNF-YC9-SRMT-PtoRD26 module regulates the high saline tolerance of a triploid poplar
Source: Genome Biol. 2022 Jul 7;23:148. doi: 10.1186/s13059-022-02718-7 (PMC9264554; doi:10.1186/s13059-022-02718-7)
Supplement: Supplementary file 14 — Additional file 14. Uncropped images for the blots in Figs. 3D, 5C, 6B, 6C, 6E, 6F, 8D, and supplementary Figure 15. (PPTX 9666 kb) [file 13059_2022_2718_MOESM14_ESM.pptx]

## Slide 1
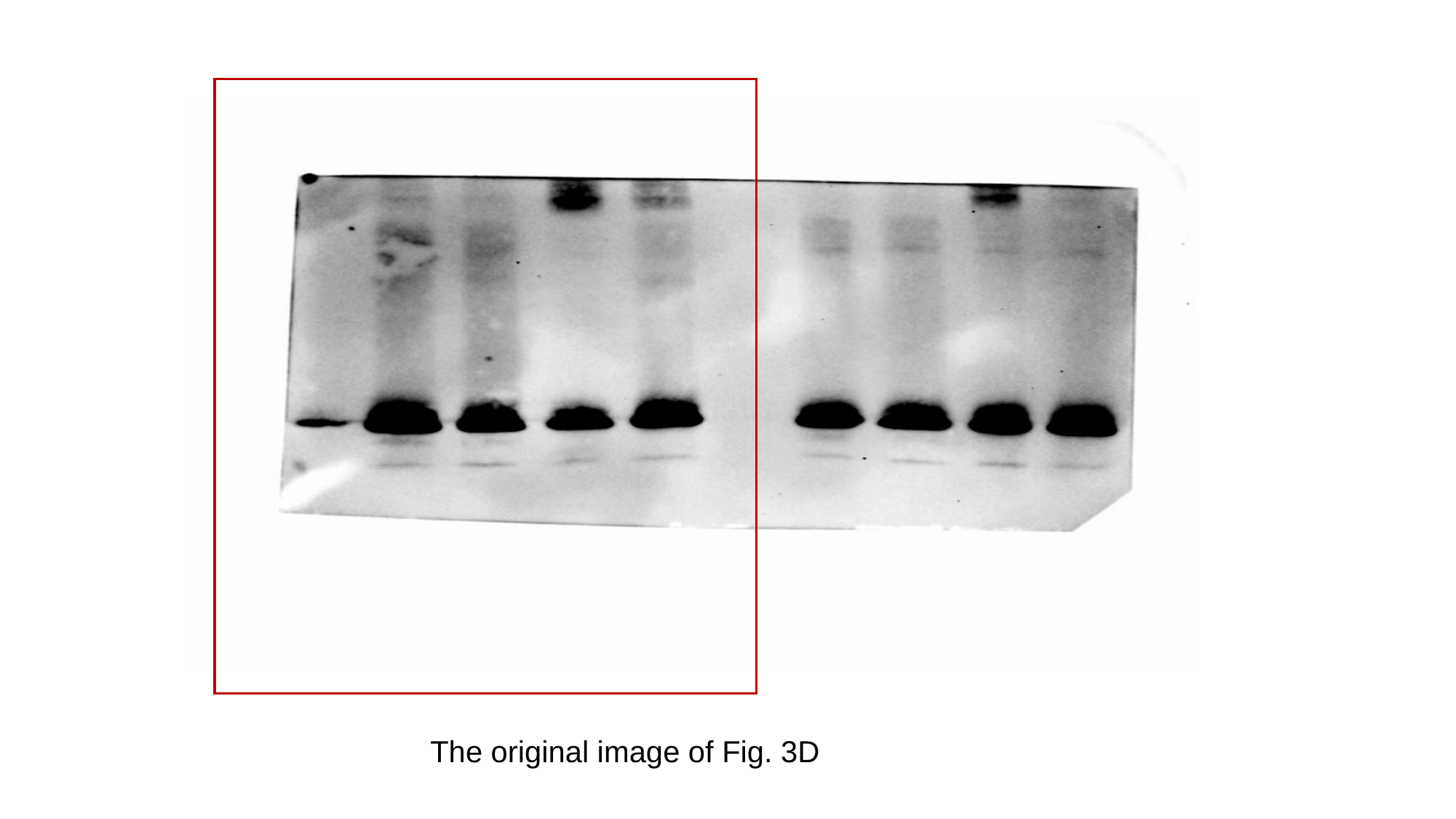

The original image of Fig. 3D

## Slide 2
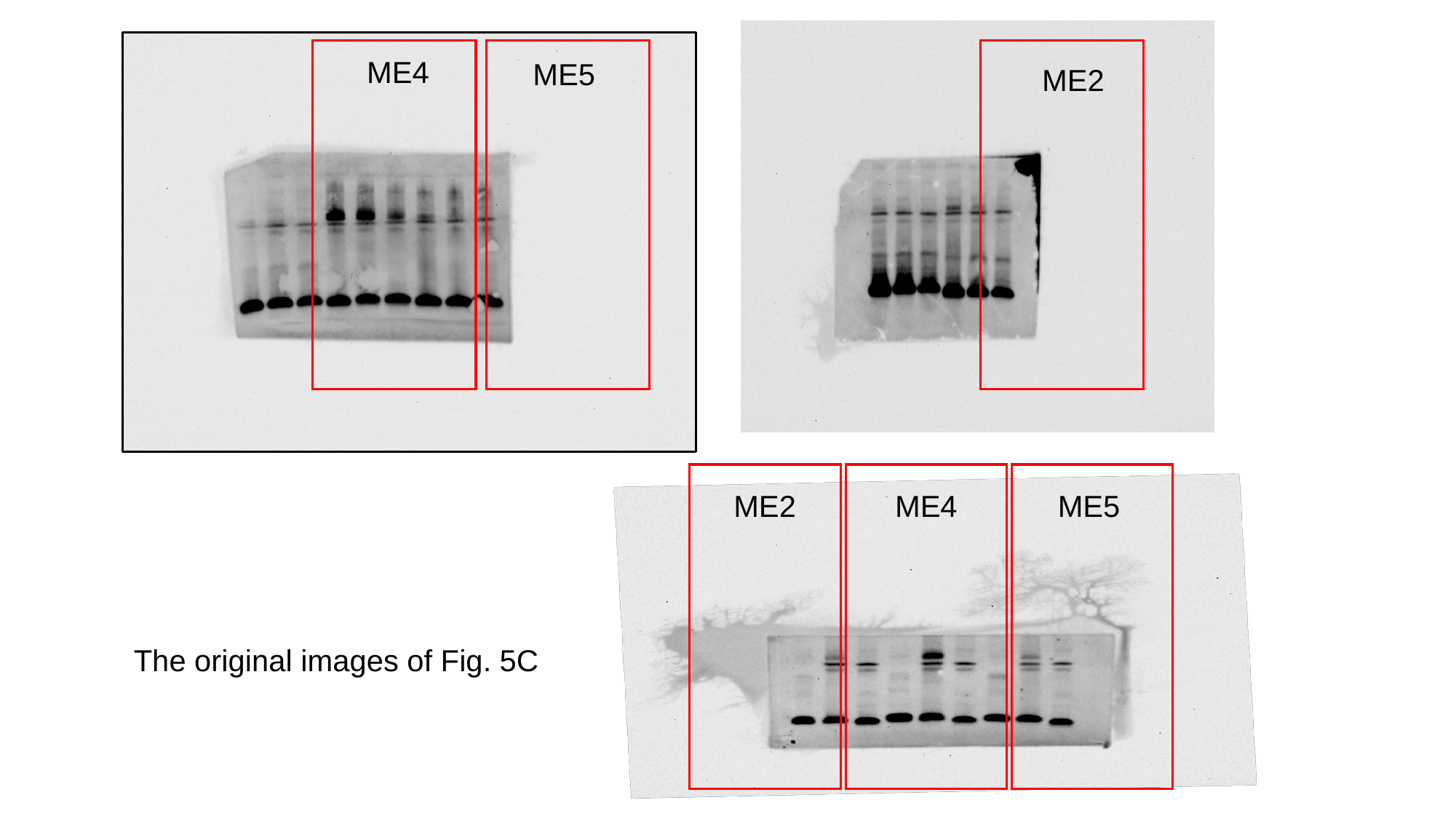

ME4
ME5
ME2
ME2
ME4
ME5
The original images of Fig. 5C

## Slide 3
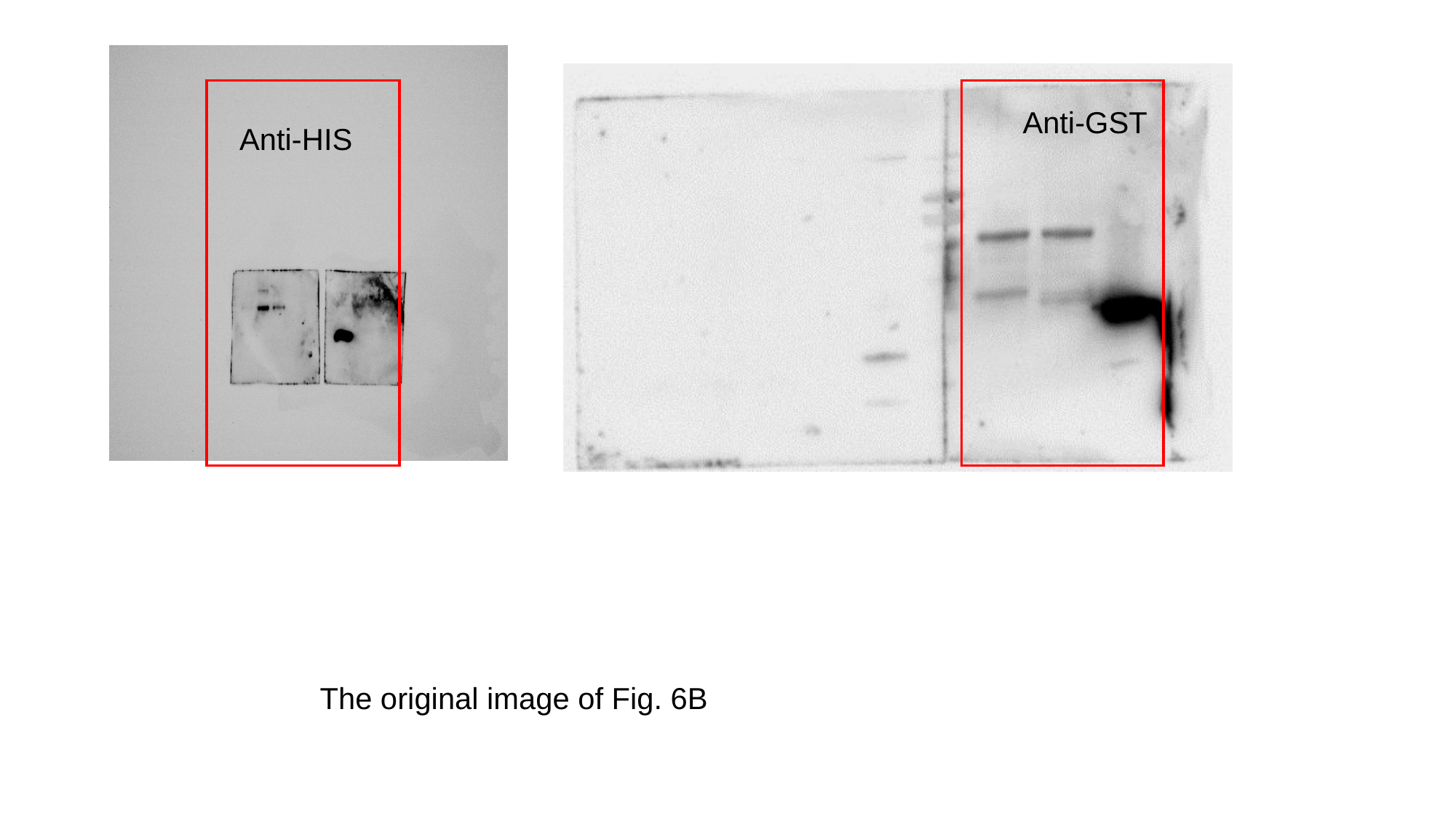

Anti-GST
Anti-HIS
The original image of Fig. 6B

## Slide 4
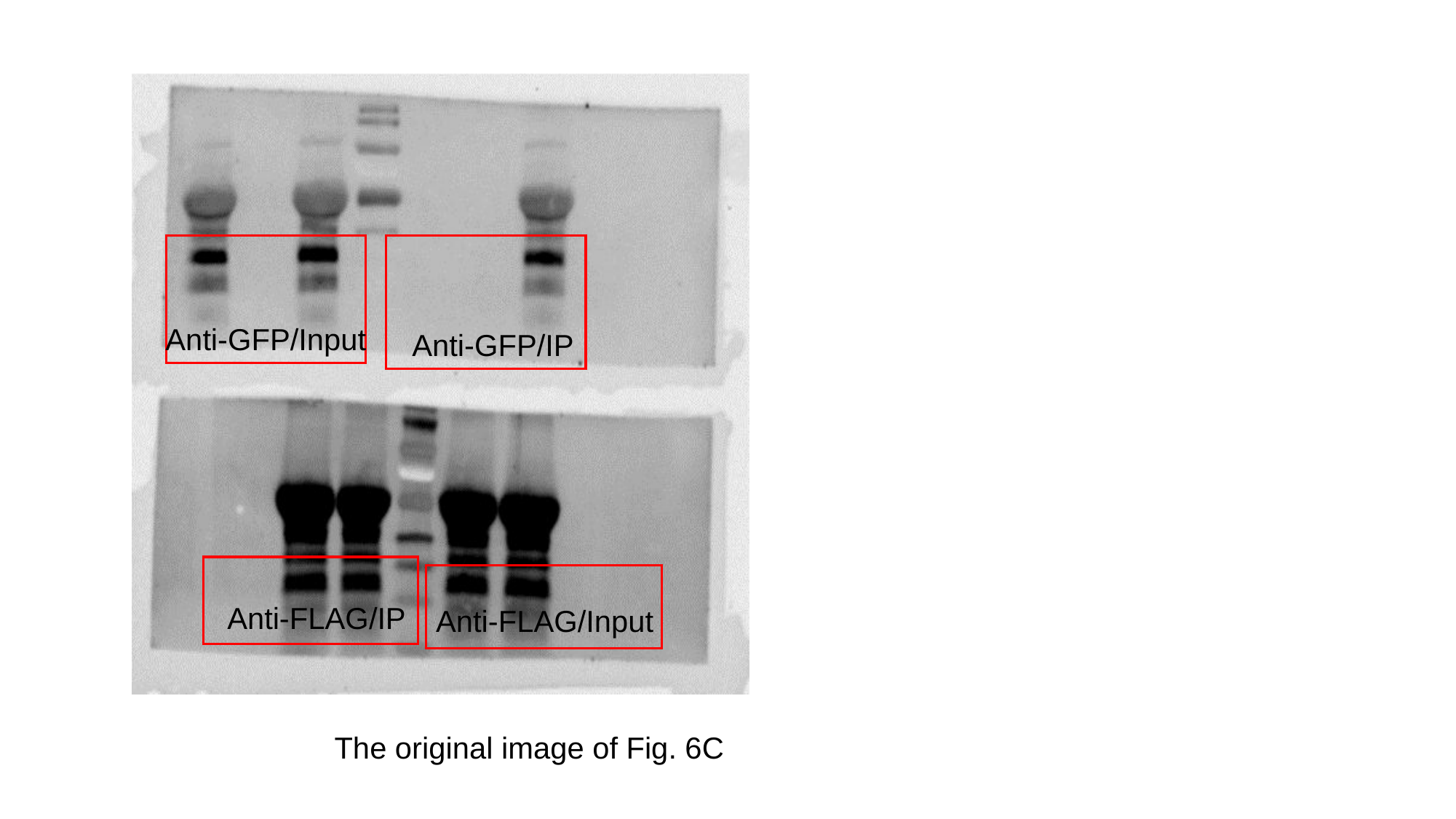

Anti-GFP/Input
Anti-GFP/IP
Anti-FLAG/IP
Anti-FLAG/Input
The original image of Fig. 6C

## Slide 5
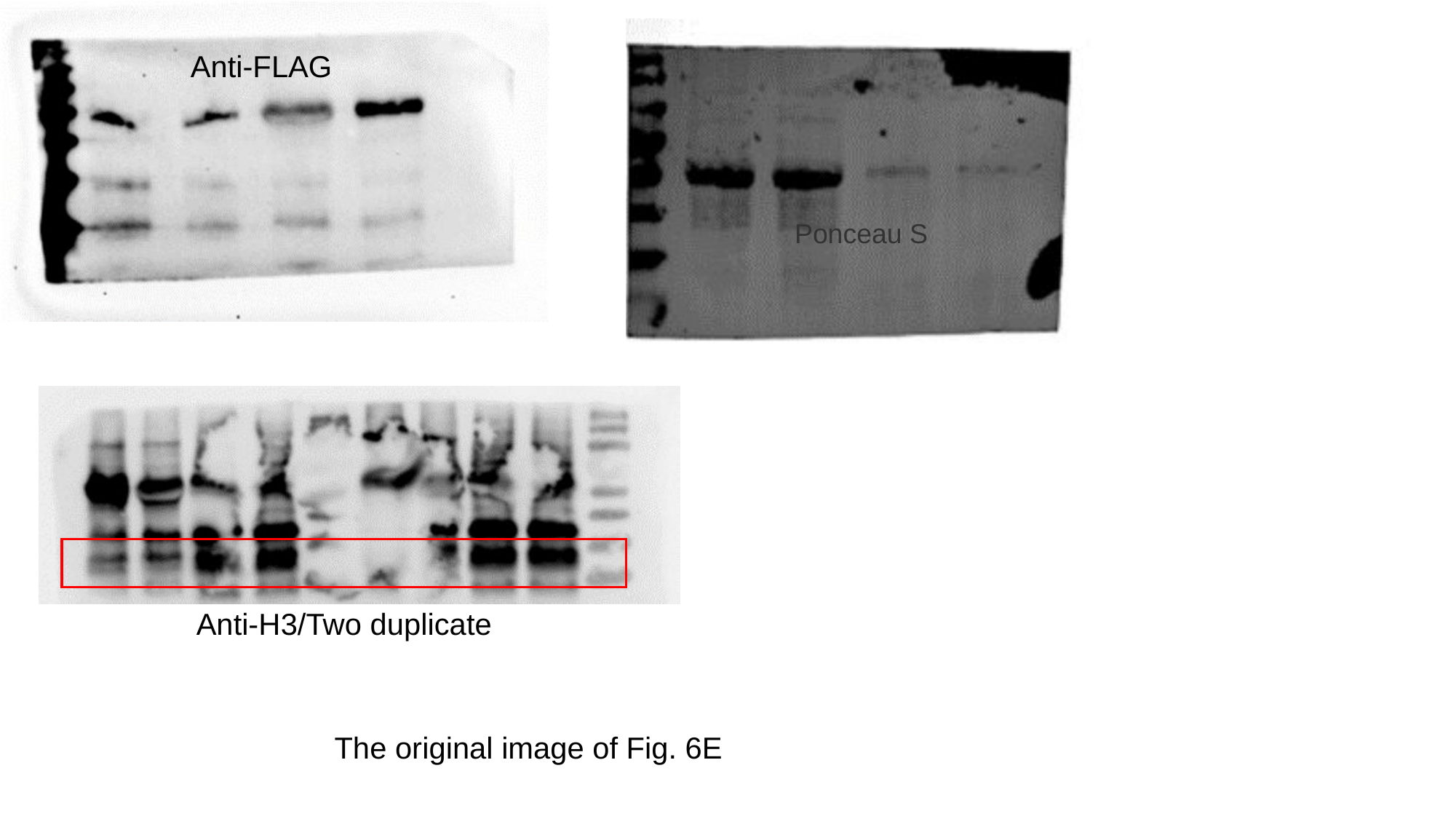

Anti-FLAG
Ponceau S
Anti-H3/Two duplicate
The original image of Fig. 6E

## Slide 6
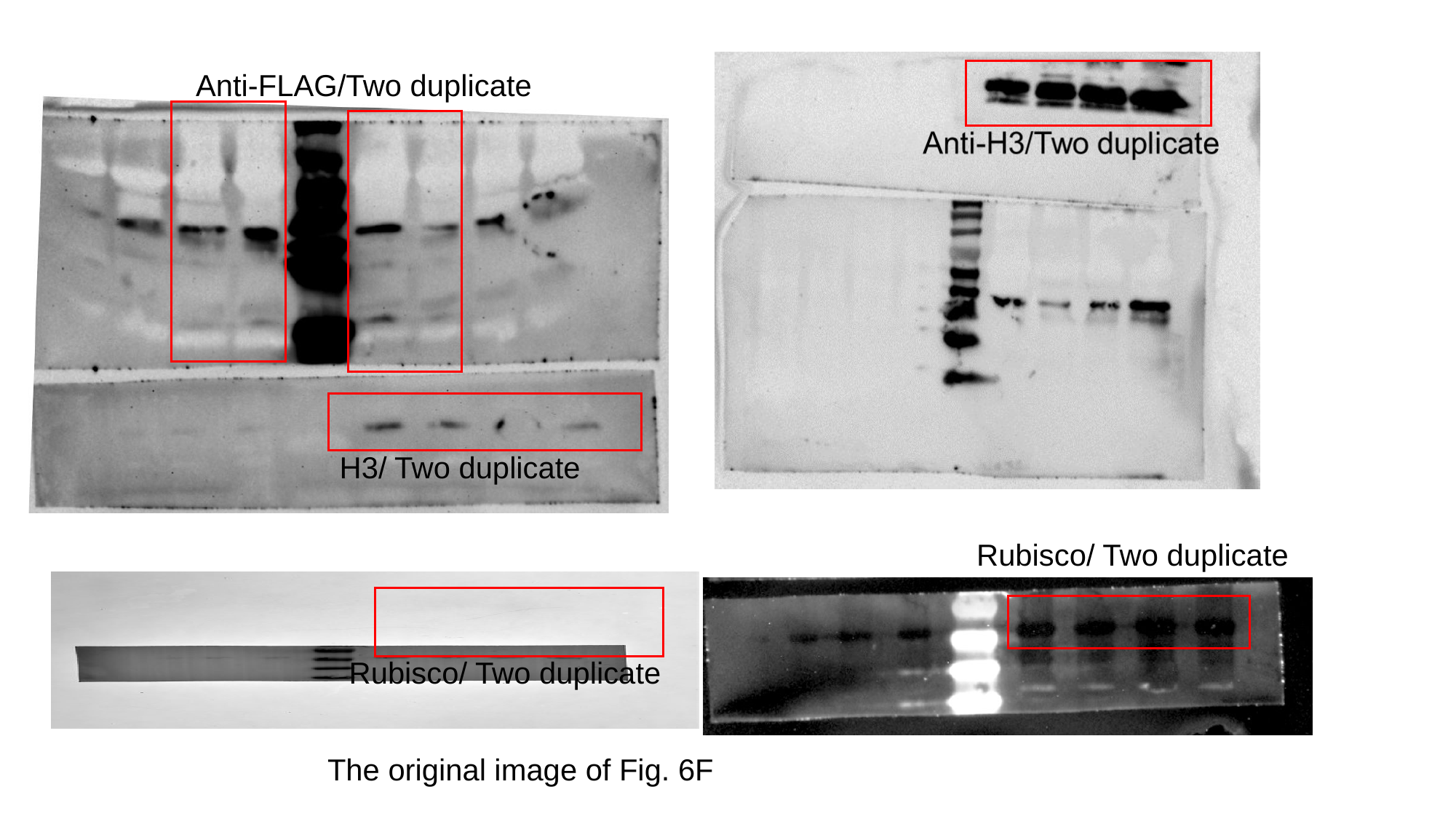

Anti-FLAG/Two duplicate
Anti-H3/Two duplicate
H3/ Two duplicate
Rubisco/ Two duplicate
Rubisco/ Two duplicate
The original image of Fig. 6F

## Slide 7
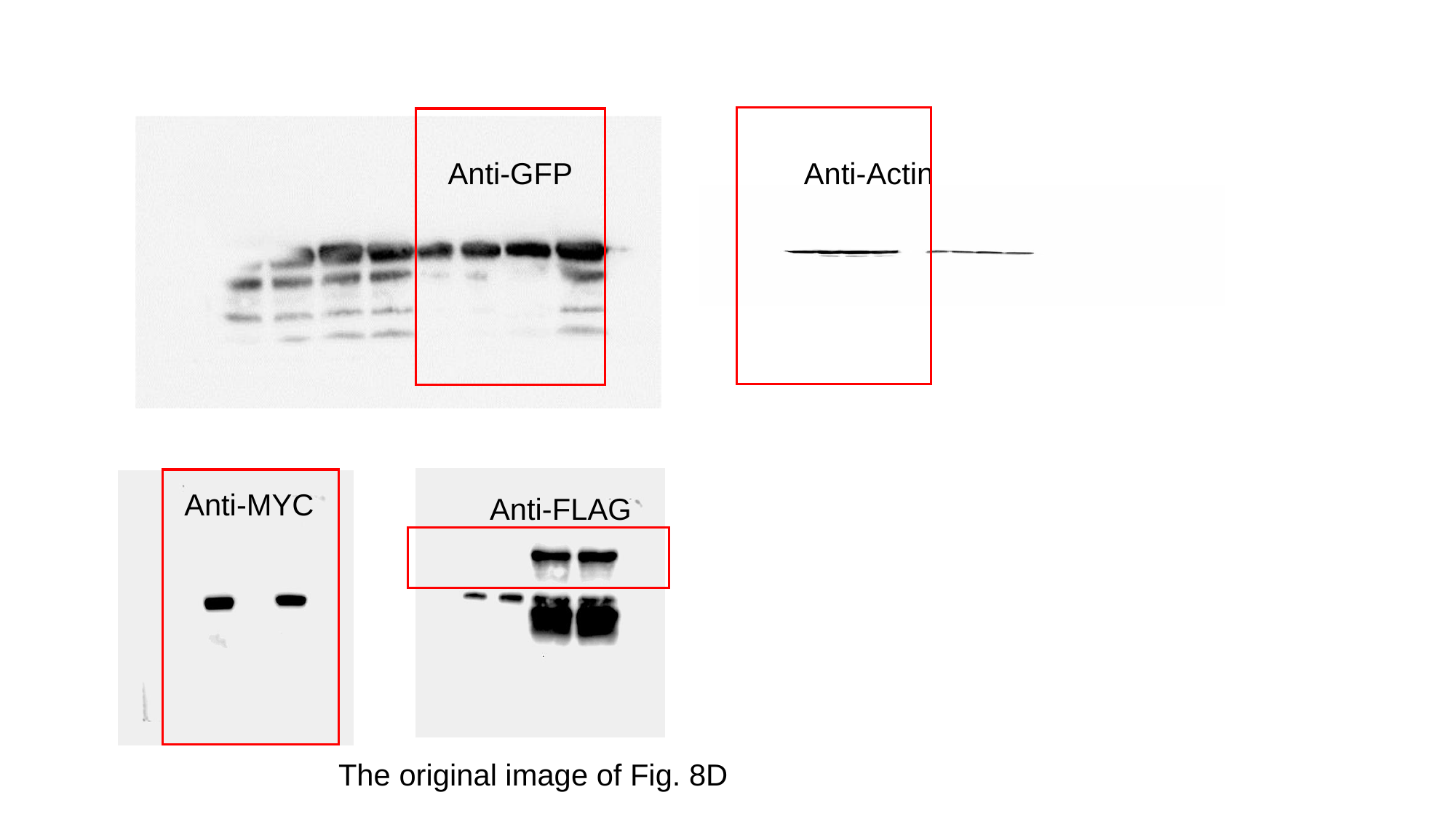

Anti-GFP
Anti-Actin
Anti-MYC
Anti-FLAG
The original image of Fig. 8D

## Slide 8
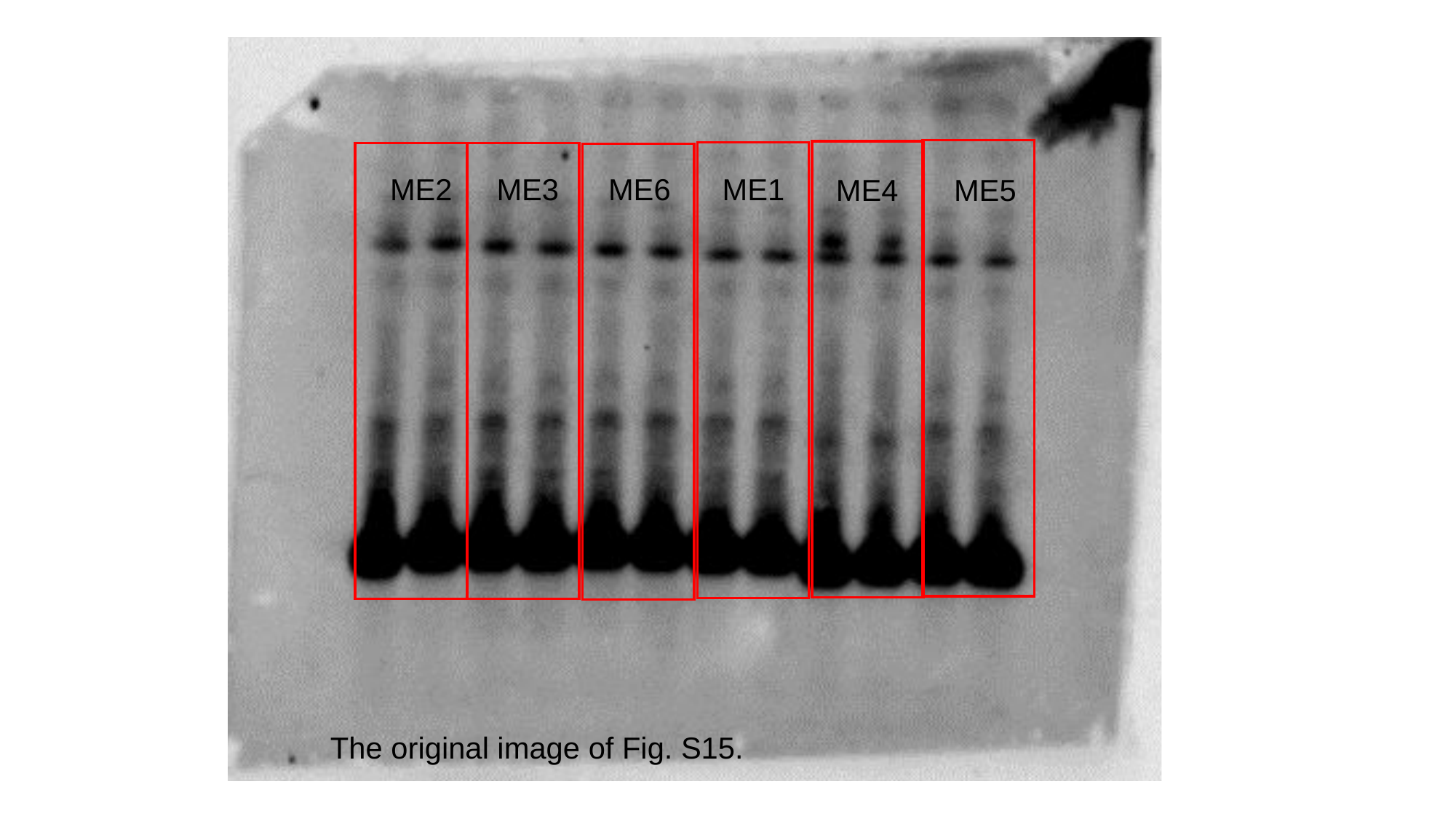

ME2
ME3
ME6
ME1
ME4
ME5
The original image of Fig. S15.
