# Supplementary material for: PtoNF-YC9-SRMT-PtoRD26 module regulates the high saline tolerance of a triploid poplar
Source: Genome Biol. 2022 Jul 7;23:148. doi: 10.1186/s13059-022-02718-7 (PMC9264554; doi:10.1186/s13059-022-02718-7)
Supplement: Supplementary file 15 — Additional file 15. Review history. [file 13059_2022_2718_MOESM15_ESM.docx]

Review History

**First round of review**

**Reviewer 1**

**Were you able to assess all statistics in the manuscript, including the appropriateness of statistical tests used?**

Yes, and I have assessed the statistics in my report.

**Comments to author:**

Study on molecular mechanisms of stress tolerance in poplar facilitates an understanding of woody plants' adaptation in various environments. The manuscript by Jiang et al. reported a haplotype-resolved genome of triploid Chinese white poplar, and revealed a PtoNF-YC9-SRMT-PtoRD26 module that is involved the regulation of tolerance to high salinity. In the proposed model, ABA triggers the shuttling of PtoNF-YC9 protein into the nucleus, and the nucleus-localized PtoNF-YC9 interacts with the MYB protein SRMT to directly activate the expression of PtoRD26, a positive regulator of high salinity response. In turn, PtoRD26 can also promote the SRMT expression to form a positive feedback loop. This is a novel and interesting study. The authors performed a plenty of experiments supporting the detailed regulations they claimed. They also generated different transgenic poplar lines for three genes, which is not easy for a woody plant. Basically, this manuscript is well-written, but there are still some ambiguous issues that should be addressed.

Major concerns:

1. The authors performed the expression analyses of transformed genes and their downstream genes in all transgenic lines (Figs. 2H, 4I, and 7D; Figs. S3, S9, S14, and S18), but they did not provide the growth details. Were these examined plants grown under normal conditions? How about the case under high salinity? If the stress-induced genes such as PtoHB7 and PtoERD7 were activated by ectopic SRMT or PtoRD26 under normal conditions, does the activation require an ABA-induced shuttling of PtoNF-YC9 into the nucleus? If not, the model in Fig. 8F might be modified.

2. As we know, ABA and stress response retard growth when confer the plant tolerance to stress conditions. Results in Fig. S10 confirmed that SRMT gene is necessary for ABA-mediated growth inhibition. However, many stress-inducible genes were activated by ectopic PtoNF-YC9, SRMT, or PtoRD26, why did not these overexpressed plants show an obvious phenotype compared with wt? Is it suggested that these inducible downstream genes are not relevant to growth inhibition?

3. The authors showed that there are variations between three allelic genes of PtoNF-YC9, SRMT, and PtoRD26 in triploid poplar. It is wondered which allelic genes were used for the overexpression construct of three genes? Any potential functional difference in these alleles?

4. In Fig. 8A, I am curious why the authors did not generate the PtoNF-YC9-OE/SRMT-RNAi line but a double RNAi one? It would provide stronger evidence to support the genetic relationship between PtoNF-YC9 and SRMT.

5. The authors showed that ABA can trigger the shuttling of PtoNF-YC9 into the nucleus. It is better to verify whether NaCl has a similar function, which would be helpful for the understanding of PtoNF-YC9-SRMT-PtoRD26 function in high salinity tolerance.

6. In Fig. 6E, to verify the fractions used in WB are from cytoplasm or the nucleus, H3 should be examined in cytoplasmic fraction and rubisco be examined in the nuclear fraction, respectively.

Minor concerns:

7. Fig. S2 was not cited in the text.

8. In Fig. 2D-F, 4D-F, and 7E-G, how long were the cuttings treated by NaCl in WPM medium? I did not find it in the text, legends, and method section.

9. In Fig. S6D, please indicate which plant protoplasts were used.

10. In Fig. 3B, please indicate what is ccdb.

11. In Fig. 3D, the shift band was cut too close to figure margin. Please remade it.

12. L216, "node）".

13. In Fig 5G and L280, the authors claimed that SRMT was induced by 8 h post treatment and reached a maximum after 24 h. Actually, there was an induced peak for SRMT at 2 h treatment.

**Reviewer 2**

**Were you able to assess all statistics in the manuscript, including the appropriateness of statistical tests used?**

Yes, and I have assessed the statistics in my report.

**Comments to author:**

The manuscript titled "PtoNF-YC9-SRMT-PtoRD26 module regulates the high saline tolerance of a triploid poplar" provides a chromosome-scale genome assembly and annotation for Chinese white poplar (P.× tomentosa Carr. clone 741), an allotriploid polar of great economic and ecological importance. Compared to diploid polars, this triploid polar is more tolerant to salinity stress. To elucidate the tolerance mechanism, the authors generated transcriptomic data from the triploid poplar with and without salt treatment, and identified PtoRD26 as an important candidate. The authors further found SRMT, a R2R3 MYB transcription factor, co-expressed with PtoRD26 in a co-expression analysis and SRMT' interacting partner PtoNF-YC9, a NUCLEAR FACTOR Y subunit C (NF-YC) transcription factor, in a yeast two-hybrid assay. To functionally characterize the functions of these identified candidates and the relationship between them, the authors generated single, double mutant poplars of these candidates. The authors demonstrated that ABA mediates PtoNF-YC9 shuttling into the nucleus, where it interacts with SRMT and induces the expression of PtoRD26, which in turn regulates SRMT. Overall, the manuscript provides informative and useful genomic resources for a triploid polar and insights into an ABA dependent salt stress signaling pathway. However, the authors should provide additional details in the use of their methods, experiments and descriptions for results which lack several critical details listed below.

Selected major comments:

1. Please provide experimental details, especially the duration and strength of treatments for all experiments, number of biological replicates for each experiment, number of independent transgenic lines, how the triploid genomes were taken into consideration when mapping RNA-seq reads, how the transcriptome data was generated for the co-expression analysis (Line 1066-1067 "Co-expression network of stress related genes was constructed from data of 62 poplar transcriptomes"), how the transcriptome data was generated for Fig. 4G, the length of the promoter used and how they promoters were mutated.

2. Since PtoRD26, SRMT, PtoNF-YC9 are proposed to function together under salt stress, have the authors checked the localization of PtoRD26, PtoNF-YC9 in vivo under salt stress to see if they are expressed in the same tissue (only the localization of SRMT is available in the current manuscript)?

3. In Fig 1B, DEGs are distributed at the two ends of the log2 fold changes spectrum for both up- and down-regulated ones, the authors should look into the data and provide an explanation for this strange pattern. In addition, the author should clarify which gene copy was labeled since they come from a triploid genome.

4. For most of the candidates characterized in this study and their promoters, the authors barely identify any variation in their sequences. Have the authors checked the sequence similarity between all paralogs (and probably their promoters) from the three sets for genomes to see if this is a universal pattern?

Selected minor comments:

1. Most the plant pictures in the main figures are barely readable, please provide clear plant pictures and closeup pictures of leaves whenever mentioned in the manuscript.

2. Please indicate what two asterisks and one asterisk represent in all main and supplement figures.

3. For all the figures where some physiological measurements (e.g. Na content) were determined, please clarify why there are sometimes two mutant lines available, and sometimes only one.

4. Please add the estimated genome size for hybrid 741 if available.

5. Line 133: Please add the justification for selecting 4 hours of treatment and 3rd node leave.

6. Line 216-218: Please clarify why "SRMT-RNAi poplars suffered dehydration after just one day".

7. Fig. 4G and H: how is Control different from WT sample?

8. Line 259: it seems that GST-SRMT strong binds to ME4, instead of ME5 based on Fig. 5C. Please make the corrections in the manuscript or justify why ME5 seems bound strongly to GST-SRMT.

**Authors Response**

**Point-by-point responses to the reviewers’ comments:**

Reviewer #1:

Study on molecular mechanisms of stress tolerance in poplar facilitates an understanding of woody plants' adaptation in various environments. The manuscript by Jiang et al. reported a haplotype-resolved genome of triploid Chinese white poplar, and revealed a PtoNF-YC9-SRMT-PtoRD26 module that is involved the regulation of tolerance to high salinity. In the proposed model, ABA triggers the shuttling of PtoNF-YC9 protein into the nucleus, and the nucleus-localized PtoNF-YC9 interacts with the MYB protein SRMT to directly activate the expression of PtoRD26, a positive regulator of high salinity response. In turn, PtoRD26 can also promote the SRMT expression to form a positive feedback loop. This is a novel and interesting study. The authors performed a plenty of experiments supporting the detailed regulations they claimed. They also generated different transgenic poplar lines for three genes, which is not easy for a woody plant. Basically, this manuscript is well-written, but there are still some ambiguous issues that should be addressed.

***Response:*** *We appreciate the reviewer’s positive comments on our work. In the revised manuscript, we have responded to each of the comments point by point, and clearly pointed out the location of the revision. We very much agree with the reviewer’s suggestion, and have performed most of experiments requested by reviewer, except the generation of PtoNF-YC9.1-OE/SRMT-RNAi line which will take several months to complete. Therefore, more related supplementary tables and figures have been added.*

Major concerns:

1.	The authors performed the expression analyses of transformed genes and their downstream genes in all transgenic lines (Figs. 2H, 4I, and 7D; Figs. S3, S9, S14, and S18), but they did not provide the growth details. Were these examined plants grown under normal conditions? How about the case under high salinity? If the stress-induced genes such as PtoHB7 and PtoERD7 were activated by ectopic SRMT or PtoRD26 under normal conditions, does the activation require an ABA-induced shuttling of PtoNF-YC9 into the nucleus? If not, the model in Fig. 8F might be modified.

***Response:*** *Thanks for your suggestion. We have measured the plant height and stem diameter of all the transgenic poplars after 15 days, 25 days and 40 days of transplanting into soil, respectively, and there were no significant differences among these plantlets. In this revised version, we have supplemented these statistical data under normal conditions (Fig. S25). However, we could not measure the plant growth under high salinity, because we treated these poplars with 300 mM NaCl solution that causing a serious stress so that the phenotypic observation had to be collected within 5-7 days after treatment, whereas the plant height and stem diameter of these plantlets did not display obviously changes in this short period. Additionally, the severe growth inhibition of SRMT overexpression poplars exposure to high concentration of ABA (Fig, S13 in the revised manuscript) indicates continuous stress may decrease growth rate but survive these poplars.*

*We examined the expression level of stress-induced genes, including PtoHB7, PtoERD7, PtoCIPK21 and PtoPUB79 in the ectopically expressed SRMT.3, PtoRD26.1 or PtoNF-YC9.1 poplars under normal conditions, and all these genes showed a significantly lower mRNA* *abundance than that after salt treatment (Fig. 2H, 4I and 7D in this revised version). This result supports that the activation of stress-related genes require an ABA- or salt-induced shuttling of PtoNF-YC9 into the nucleus. We modified some details of the model figure, though it is supported (Fig. 8F).*

2.	As we know, ABA and stress response retard growth when confer the plant tolerance to stress conditions. Results in Fig. S10 confirmed that SRMT gene is necessary for ABA-mediated growth inhibition. However, many stress-inducible genes were activated by ectopic PtoNF-YC9, SRMT, or PtoRD26, why did not these overexpressed plants show an obvious phenotype compared with WT? Is it suggested that these inducible downstream genes are not relevant to growth inhibition?

***Response:*** *As mentioned above, we have not observed the significant differences in plant height and stem diameter of these transgenic poplars under normal condition (Fig. S25). In addition, overexpressed SRMT.3 up-regulated PtoHB7 and PtoPUB79 to some extent without stresses, but their expression levels are much lower than those after salt stress (Fig. 4I), indicating that enhancement of SRMT without high ABA level or salt cannot lead to comprehensive ABA signaling. The ABA-triggered PtoNF-YC9 nuclear shuttling is important to activation of genes relevant to stress response or even growth inhibition, therefore the significant difference in growth inhibition among SRMT.3-OE, SRMT-RNAi and WT was observed only when supplementing high content of ABA (5 μM) (Fig. S13).*

3.	The authors showed that there are variations between three allelic genes of *PtoNF-YC9*, *SRMT*, and *PtoRD26* in triploid poplar. It is wondered which allelic genes were used for the overexpression construct of three genes? Any potential functional difference in these alleles?

***Response:*** *Thank you very much. We overexpressed PtoRD26.1 (P.x_tomentosa47952), SRMT.3 (P.x_tomentosa71644) and PtoNF-YC9.1 (P.x_tomentosa23609) in the poplars, respectively. To examine whether any potential functional difference in these alleles, we performed a Dual-LUC assay. The results indicated that these alleles have similar transcriptional activation activity to the target promoters (Fig. S9, S11, S24), therefore, their functions are not obviously different in this aspect. These results were also added in the revised manuscript.*

4.	In Fig. 8A, I am curious why the authors did not generate the PtoNF-YC9-OE/SRMT-RNAi line but a double RNAi one? It would provide stronger evidence to support the genetic relationship between PtoNF-YC9 and SRMT.

***Response:*** *We strongly agree with the reviewer. In fact, we had designed two types of lines, including the PtoNF-YC9-OE/SRMT-RNAi line and the double RNAi one, however, we had failed to obtain the PtoNF-YC9-OE/SRMT-RNAi poplars. If we try again, it will take at least four months to generate the PtoNF-YC9-OE/SRMT-RNAi line and another three months to propagate these poplars and evaluate their tolerance to salt stress.*

5.	The authors showed that ABA can trigger the shuttling of PtoNF-YC9 into the nucleus. It is better to verify whether NaCl has a similar function, which would be helpful for the understanding of PtoNF-YC9-SRMT-PtoRD26 function in high salinity tolerance.

***Response:*** *Thanks for your suggestion. We have attempted to observe the NaCl-stimulated translocation of PtoNF-YC9 in the protoplasts, however, these protoplasts cannot endure higher Na^+^ concentration leading to fracture. Alternatively, we examined the abundance of FLAG-tagged PtoNF-YC9 in nucleus and cytoplasm of the overexpressed poplars with or without NaCl treatment using a WB assay. The result showed that PtoNF-YC9 is accumulated more in cytoplasm than in nucleus without NaCl, but the observation is conversed when exposure to salt treatment (Fig. 6F). It is consistent with the observation under ABA supplemented (Fig. 6E).*

6.	In Fig. 6E, to verify the fractions used in WB are from cytoplasm or the nucleus, H3 should be examined in cytoplasmic fraction and rubisco be examined in the nuclear fraction, respectively.

***Response:*** *Thank for your suggestion and we agree with you. In fact, when we performed this assay, we had examined the H3 in cytoplasmic fraction and rubisco in the nuclear fraction, which was not showed in the first version. Therefore, we had shown this data in this revised manuscript (Fig. 6E and F).*

Minor concerns:

7.	Fig. S2 was not cited in the text.

***Response:*** *Thank for your reminding. We have supplemented this citation in the Line 150 of the revised manuscript (Fig. S4).*

8.	In Fig. 2D-F, 4D-F, and 7E-G, how long were the cuttings treated by NaCl in WPM medium? I did not find it in the text, legends, and method section.

***Response:*** *The cuttings treated by NaCl in WPM medium for 20 days. We have supplemented this description in the “Methods and Materials” section (Line 501).*

9.	In Fig. S6D, please indicate which plant protoplasts were used.

***Response:*** *Thank you. These protoplasts were isolated from mesophyll of P.× tomentosa Carr. clone 741. We had supplemented the descriptions in the figure legend (Line 57-58 in the additional file 1).*

10.	In Fig. 3B, please indicate what is ccdb.

***Response:*** *The ccdB in the pCXSN empty vector is a negative selection marker gene to eliminate the self-ligation background after transformation (Chen et al., 2009, Plant Physiology). The encoding product of ccdB is invalid to plant and Agrobacterium strain GV3101. When we construct the effectors, the ccdB gene is replaced. We have supplemented the explanation about the ccdB in the figure legend of Fig. 3A (Line 1126).*

11.	In Fig. 3D, the shift band was cut too close to figure margin. Please remade it.

***Response:*** *Thank for your suggestion. The original image of two repeats of EMSA was showed below (protein concentration of the left one is double to the right), and we selected another group of EMSA results (the left lanes) in the revised Fig. 3D.*

12.	L216, "node）".

***Response:*** *It has been revised.*

13.	In Fig 5G and L280, the authors claimed that SRMT was induced by 8 h post treatment and reached a maximum after 24 h. Actually, there was an induced peak for SRMT at 2 h treatment.

***Response:*** *Thank for your reminding. We have re-written this description (Line 290-291) as “SRMT was slightly up-regulated at 2 h after treatment but decreased, and it was significantly induced by 8 h post treatment and reached a maximum after 24 h.”*

Reviewer #2:

The manuscript titled "PtoNF-YC9-SRMT-PtoRD26 module regulates the high saline tolerance of a triploid poplar" provides a chromosome-scale genome assembly and annotation for Chinese white poplar (*P.× tomentosa* Carr. clone 741), an allotriploid polar of great economic and ecological importance. Compared to diploid polars, this triploid polar is more tolerant to salinity stress. To elucidate the tolerance mechanism, the authors generated transcriptomic data from the triploid poplar with and without salt treatment, and identified PtoRD26 as an important candidate. The authors further found SRMT, a R2R3 MYB transcription factor, co-expressed with PtoRD26 in a co-expression analysis and SRMT' interacting partner PtoNF-YC9, a NUCLEAR FACTOR Y subunit C (NF-YC) transcription factor, in a yeast two-hybrid assay. To functionally characterize the functions of these identified candidates and the relationship between them, the authors generated single, double mutant poplars of these candidates. The authors demonstrated that ABA mediates PtoNF-YC9 shuttling into the nucleus, where it interacts with SRMT and induces the expression of PtoRD26, which in turn regulates SRMT. Overall, the manuscript provides informative and useful genomic resources for a triploid polar and insights into an ABA dependent salt stress signaling pathway. However, the authors should provide additional details in the use of their methods, experiments and descriptions for results which lack several critical details listed below.

***Response:*** *We appreciate the reviewer’s positive comments on our work. In the revised manuscript, we have responded to each of the comments point by point, and clearly pointed out the location of the revision. We very much agree with the reviewer’s suggestion, and supplemented the experiments and analysis requested by reviewers.*

Selected major comments:

1. Please provide experimental details, especially the duration and strength of treatments for all experiments, number of biological replicates for each experiment, number of independent transgenic lines, how the triploid genomes were taken into consideration when mapping RNA-seq reads, how the transcriptome data was generated for the co-expression analysis (Line 1066-1067 "Co-expression network of stress related genes was constructed from data of 62 poplar transcriptomes"), how the transcriptome data was generated for Fig. 4G, the length of the promoter used and how they promoters were mutated.

***Response:*** *Thank for your suggestion. More detailed experimental description has been supplemented in the “Methods and Materials” section and the figure legends.*

*For RNA-seq mapping, we only kept the best alignment for each read according to the methods mentioned in the published tea plant haplotype genome (Zhang et al., 2021, Nature Genetics) (Line 540-541). For co-expression analysis,* *we generated transcriptomic data of 17 samples from triploid poplar and 9 samples from P. abla var. pyramidalis, and collected 36 other transcriptomic data of P. abla var. pyramidalis that have been released in NCBI (Line 1109-1113).* *For DEGs analysis in Fig. 4G,* *we applied the RNA-seq samples of triploid poplar except for WT-4 and NaCl-4. The detailed information of the samples was added in Table S3 and corresponding figures in the revised manuscript.*

*The promoter length of all genes in this study is 1,500 bp. We did not mutate the promoter* *directly but mutated the critical cis-acting elements for Dual-Luc assay and EMSA (Table S12).*

*EMSA-PtoRD26pro-SRMTbind-ME2-F:	TCTCGAAGTTTGACCTTCCCTTCATCAATC*

*EMSA-mutantPtoRD26pro-SRMTbind-ME2-F:	TCTCGAAGTTTAAAAAAAACTTCATCAATC*

*EMSA-PtoRD26pro-SRMTbind-ME4-F:	CAAAATGAAACACGACCAAACTGTGGACATTAG*

*EMSA-mutantPtoRD26pro-SRMTbind-ME4-F:	CAAAATGAAACACAAAAAAAATGTGGACATTAG*

*EMSA-PtoRD26pro-SRMTbind-ME5-F:	ACTTGCACATGGATAAGGTCGGCTATTTGATG*

*EMSA-mutantPtoRD26pro-SRMTbind-ME5-F:	ACTTGCACATGGAAAAAAAAGGCTATTTGATG*

2. Since PtoRD26, SRMT, PtoNF-YC9 are proposed to function together under salt stress, have the authors checked the localization of PtoRD26, PtoNF-YC9 in vivo under salt stress to see if they are expressed in the same tissue (only the localization of SRMT is available in the current manuscript)?

***Response:*** *Thank you. We have supplemented the expression patterns of PtoRD26, SRMT and PtoNF-YC9 in young leave, mature leave, petiole, stem and root of the salt-stressed triploid poplar. These results showed that both PtoRD26 and SRMT are induced mainly in mature leaves, petiole and root by salt treatment but the expression of PtoNF-YC9 displayed no significant differences before and after salt treatment (Fig. S4, S8A, S20B-C in this revised manuscript).*

3. In Fig 1B, DEGs are distributed at the two ends of the log2 fold changes spectrum for both up- and down-regulated ones, the authors should look into the data and provide an explanation for this strange pattern. In addition, the author should clarify which gene copy was labeled since they come from a triploid genome.

***Response:*** *Thank you for your comment. We have supplemented the dot plot of the distribution of gene expression level where the x and y axis corresponded to the log(FPKM) value of the control and salt stressed sample, respectively. We found that some DEGs that distributed at the two ends of the volcano plot were close to the two axis, such as PtoGSTU19.3, indicating that the expression level of these DEGs in one comparison sample was almost equal to 0 but higher in the other sample, resulting in a significant high fold change value which corresponded to the extreme position in the volcano plot in Fig. 1B. In addition, we have checked the expression pattern of the alleles labeled in the volcano plot, and found that all the three alleles were up-regulated for each gene except for PtoGSTU19 where only PtoGSTU19.3 was up-regulated. All these alleles have been labeled in the volcano plot of Fig. 1B in this revised version.*

4. For most of the candidates characterized in this study and their promoters, the authors barely identify any variation in their sequences. Have the authors checked the sequence similarity between all paralogs (and probably their promoters) from the three sets for genomes to see if this is a universal pattern?

***Response:*** *Thank you for your reminding. We have calculated the sequence similarity of the alleles between each two haplotypes, including coding sequences, protein sequences and their 1.5 kb promoter sequences. The results indicated that alleles from the three haplotypes had high similarity where the medium value of coding sequences and protein sequences were greater than 95% and the promoter similarity was greater than 70%. This result was added in Fig. S2* *of the revised manuscript.*

Selected minor comments:

1. Most the plant pictures in the main figures are barely readable, please provide clear plant pictures and closeup pictures of leaves whenever mentioned in the manuscript.

***Response:*** *A good suggestion. We have displayed the closeup pictures of leaves after salt treatment, including Fig. 2A, 4A, 5E, 7A and 8A.*

2. Please indicate what two asterisks and one asterisk represent in all main and supplement figures.

***Response:*** *Thank for your suggestion. We have indicated *P < 0.05 and **P < 0.01 in all legends of main and supplement figures in the revised manuscript.*

3. For all the figures where some physiological measurements (e.g. Na^+^ content) were determined, please clarify why there are sometimes two mutant lines available, and sometimes only one.

***Response:*** *In fact, all physiological measurements were performed based on at least two independent lines. In this revised manuscript, we displayed these data from two independent lines (Fig. 2C, 2E-F, 4C, 4E-F, 5F, 7C and 7F-G).*

4. Please add the estimated genome size for hybrid 741 if available.

***Response:*** *Thank for your suggestion. We have supplemented its estimated genome size by the flow cytometry. The P. alba var. pyramidalis (1.04 pg/2C) was used as internal reference standard (Zhang et al., 2020, Molecular Ecology Resources). Comparing with the fluorescence area signals (FL2-A) between the P. alba var. pyramidalis and the P. × tomentosa Carr. clone 741, the flow cytometry analysis provides an estimation of 1.61 pg DNA, which is equivalent to 1.58 Gb genome of P. × tomentosa Carr. clone 741 based on the formula: 1 pg = 0.978 Gb. (Fig. S1 in this revised manuscript)*

5. Line 133: Please add the justification for selecting 4 hours of treatment and 3^rd^ node leaves.

***Response:*** *Thank you.* *We want to reveal the transcriptional reprogramming of this triploid* *poplar in response to salt stress at early stage. Previously, we found that some genes involved in early response to salt stress, like ERF family genes were significantly induced in the 3^rd^ node leaves after 4 h of salt treatment. Therefore, we select the 3^rd^ node leaves stressed for 4 h to perform RNA-seq analysis, and this method was mentioned in our previous article by Chen et al. (2020, Tree Physiology) that have been cited in this revised manuscript.*

6. Line 216-218: Please clarify why "SRMT-RNAi poplars suffered dehydration after just one day".

***Response:*** *We are very sorry for this unclear description. We observed that the edge of leaves of SRMT-RNAi poplars showed some withered and dehydration, and it has been revised in the new version of manuscript (Line 226-227).*

7. Fig. 4G and H: how is Control different from WT sample?

***Response:*** *The “Control” means the WT poplars were watered by ddH_2_O meanwhile other WT plants were treated by NaCl solution. “WT” here indicates the non-transgenic poplars grew under the identical condition with the SRMT-OE poplar without any artificial treatments. We have supplemented the instruction in the legend of Fig. 4 (Line 1154-1157).*

8. Line 259: it seems that GST-SRMT strong binds to ME4, instead of ME5 based on Fig. 5C. Please make the corrections in the manuscript or justify why ME5 seems bound strongly to GST-SRMT.

***Response:*** *Thank you. It has been revised (Line 267-269).*

*Reference:*

*Chen S, Songkumarn P, Liu J, Wang GL. A versatile zero background T-vector system for gene cloning and functional genomics. Plant Physiol. 2009 Jul;150(3):1111-21.*

*Zhang Z, Chen Y, Zhang J, Ma X, Li Y, Li M, Wang D, Kang M, Wu H, Yang Y, Olson MS, DiFazio SP, Wan D, Liu J, Ma T. Improved genome assembly provides new insights into genome evolution in a desert poplar (Populus euphratica). Mol Ecol Resour. 2020 May;20(3).*

*Chen N, Tong S, Tang H, Zhang Z, Liu B, Lou S, Liu J, Liu H, Ma T, Jiang Y. The PalERF109 transcription factor positively regulates salt tolerance via PalHKT1;2 in Populus alba var. pyramidalis. Tree Physiol. 2020 May 30;40(6):717-730*

*Zhang X, Chen S, Shi L, Gong D, Zhang S, Zhao Q, Zhan D, Vasseur L, Wang Y, Yu J, Liao Z, Xu X, Qi R, Wang W, Ma Y, Wang P, Ye N, Ma D, Shi Y, Wang H, Ma X, Kong X, Lin J, Wei L, Ma Y, Li R, Hu G, He H, Zhang L, Ming R, Wang G, Tang H, You M. Haplotype-resolved genome assembly provides insights into evolutionary history of the tea plant Camellia sinensis. Nat Genet. 2021 Aug;53(8):1250-1259.*

**Second round of review**

**Reviewer 1**

The authors have substantially addressed my concerns. I have no questions for this updated version.

**Reviewer 2**

The authors have made substantial revisions and adequately addressed my comments by adding requested clarifications and experimental validations. I largely agree with the revisions.

A few minor comments:

1. Please make sure significant test results are shown in figures whenever applicable, e.g. in Fig 2H, 4I, 7D.

2. Please make sure all the figures are readable, especially the axis labels, including the supplementary ones, e.g Fig. S2.

**Authors Response**

**Point-by-point responses to the reviewers’ comments:**

Reviewer #2:

The authors have made substantial revisions and adequately addressed my comments by adding requested clarifications and experimental validations. I largely agree with the revisions.

A few minor comments:

1. Please make sure significant test results are shown in figures whenever applicable, e.g. in Fig 2H, 4I, 7D.

2. Please make sure all the figures are readable, especially the axis labels, including the supplementary ones, e.g Fig. S2.

***Response:*** *In this version, we have added the statistical significance results in Fig 2H, 4I, 7D, and make all the figures more readable.*
